# Gene Flow Results in High Genetic Similarity between *Sibiraea* (Rosaceae) Species in the Qinghai-Tibetan Plateau

**DOI:** 10.3389/fpls.2016.01596

**Published:** 2016-10-25

**Authors:** Peng-Cheng Fu, Qing-Bo Gao, Fa-Qi Zhang, Rui Xing, Jiu-Li Wang, Hai-Rui Liu, Shi-Long Chen

**Affiliations:** ^1^Key Laboratory of Adaptation and Evolution of Plateau Biota, Northwest Institute of Plateau Biology, Chinese Academy of SciencesXining, China; ^2^College of Life Science, Luoyang Normal UniversityLuoyang, China; ^3^College of Life Science, University of Chinese Academy of SciencesBeijing, China

**Keywords:** gene flow, genetic divergence, microsatellite, Qinghai-Tibetan Plateau, *Sibiraea*

## Abstract

Studying closely related species and divergent populations provides insight into the process of speciation. Previous studies showed that the *Sibiraea* complex's evolutionary history on the Qinghai-Tibetan Plateau (QTP) was confusing and could not be distinguishable on the molecular level. In this study, the genetic structure and gene flow of *Sibiraea laevigata* and *Sibiraea angustata* on the QTP was examined across 45 populations using 8 microsatellite loci. Microsatellites revealed high genetic diversity in *Sibiraea* populations. Most of the variance was detected within populations (87.45%) rather than between species (4.39%). We found no significant correlations between genetic and geographical distances among populations. Bayesian cluster analysis grouped all individuals in the sympatric area of *Sibiraea* into one cluster and other individuals of *S. angustata* into another. Divergence history analysis based on the approximate Bayesian computation method indicated that the populations of *S. angustata* at the sympatric area derived from the admixture of the 2 species. The assignment test assigned all individuals to populations of their own species rather than its congeneric species. Consistently, intraspecies were detected rather than interspecies first-generation migrants. The bidirectional gene flow in long-term patterns between the 2 species was asymmetric, with more from *S. angustata* to *S. laevigata*. In conclusion, the *Sibiraea* complex was distinguishable on the molecular level using microsatellite loci. We found that the high genetic similarity of the complex resulted from huge bidirectional gene flow, especially on the sympatric area where population admixtures occurred. This study sheds light on speciation with gene flow in the QTP.

## Introduction

Understanding the effect of gene flow in genetic evolution could shed light on speciation (Slatkin, 1987; Smadja and Butlin, 2011). During population divergence, gene flow is absent in the “allopatric speciation” model (Hoskin et al., 2005) but evident in the “sympatric speciation” model (Smadja and Butlin, 2011). Since gene flow constrains population differentiation and is thereby often regarded as a constraining force in evolution, speciation in the face of gene flow is generally considered difficult (Slatkin, 1987; Coyne and Orr, 2004). Nonetheless, multiple examples of speciation with gene flow now indicates that speciation with gene flow may be common (Nosil, 2008; Fitzpatrick et al., 2009; Duncan et al., 2016). Additionally, gene flow between subpopulations can maintain high levels of diversity within each subpopulation (Epps et al., 2005).

Gene flow among populations can be estimated using population genetic analyses (Pearse and Crandall, 2004; Hey, 2006) through patterns of shared genetic variation detected by molecular techniques (Cowen and Sponaugle, 2009). Genetic differentiation between distinct lineages can be quickly and easily determined by DNA fragment sequences (e.g., DNA barcodes) for multiple individuals (Mallet, 1995). Although, DNA fragment sequences revealed numerous cryptic species lacking morphological differentiation in both animals and plants (Myers et al., 2013; Su et al., 2015), they sometimes fail when groups are closely related species, let alone evolutionary complex. At present, simple sequence repeats (SSR) are widely used to characterize divergence among closely related species (e.g., Surget-Groba and Kay, 2013; Yan et al., 2015; Duncan et al., 2016). Here we present the first evidence of gene flow in a plant species complex in the Qinghai-Tibetan Plateau (QTP) based on SSR loci.

As the largest and highest plateau, with an average elevation of >4000 m, the QTP has received a great deal of attention from botanists. The QTP uplift significantly changed the climate and environment of Asia (Zhang et al., 2000; Zheng et al., 2002). Additionally, Pleistocene climate change played an important role in shaping geographical patterns of intraspecific genetic diversity (Hewitt, 2000, 2004; Qiu et al., 2011; Liu et al., 2012). The QTP uplift also affected glacial environments; therefore, species in the QTP were probably more affected by Quaternary glaciations than those in other regions of the world at similar latitude (Zhang et al., 2000). During Pleistocene glaciations, the spread of large ice sheets affected species at high to mid-latitudes (Willis and Niklas, 2004) and fragmented geographical distributions of many species. This fragmentation promoted conditions favorable for allopatric divergence among isolated populations and, potentially, speciation (Wen et al., 2014). Mountains in the QTP with the potential to act as barriers to movement can result in genetic structures ranging from panmixia (Lessios et al., 2003) to complete separation (Baums et al., 2012). Therefore, species on the QTP likely experienced an evolution history different from other areas of the world (Liu et al., 2014; Wen et al., 2014).

*Sibiraea angustata* (Rehder) Hand.-Mazz. and *Sibiraea laevigata* (L.) Maxim. (Rosaceae) are the 2 common *Sibiraea* species in the QTP (Gu and Alexander, 2003). Preliminary analyses of chromosome numbers confirmed that they are diploid species (2n = 18; Fu et al., 2016). Although morphologically similar, they can be distinguished by the pubescent peduncles and pedicels of *S. angustata* and the glabrous peduncles and pedicels in *S. laevigata*. Furthermore, *S. laevigata* has a much smaller distribution area and is less abundant in the QTP than *S. angustata*, with which it is always sympatric in the QTP. Field work showed that both species are outcrossing, exhibit identical flowering times, and have a number of light seeds with wings (Fu et al., 2016). This phylogeographic study based on chloroplast (cp) DNA and internal transcribed spacer (ITS) sequences also indicated the high genetic similarity of the 2 species, and classified complex individuals into 3 clades: the Northern, the Central, and the Southern. However, these clades failed to correspond to the different species. *Sibiraea laevigata* shared all its haplotypes with *S. angustata*, and phylogenetic analysis failed to distinguish them. Therefore, these previous studies raised 2 important questions concerning their genetic similarity: (i) Whether the complex could be genetically distinguished using microsatellite loci and (ii) What the reason was for the high genetic similarity and whether gene flow contributed to it. This study addresses these questions through genetic variation analysis based on 8 unlinked microsatellite loci.

## Materials and methods

### Study area and sampling

**Figure 1:**
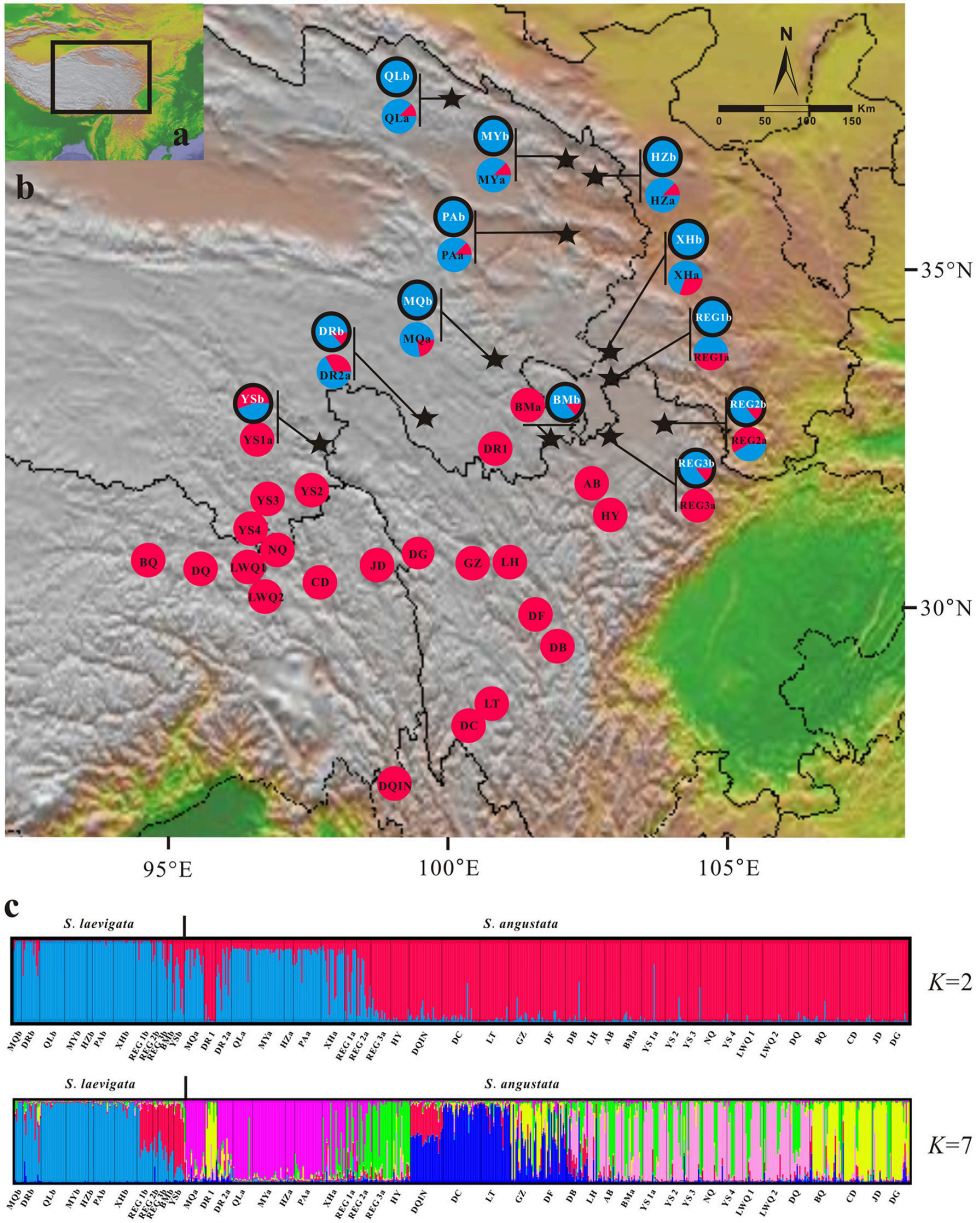
**Genetic structure of ***Sibiraea*** populations in the Qinghai-Tibetan Plateau. (A)** Sampling area; **(B)** Genetic structure of *Sibiraea* populations based on *K* = 2 genetic clusters; **(C)** Proportional membership of 55 *Sibiraea* populations to *K* = 2 and 7 genetic clusters. Individuals are represented by a single vertical column divided into 2 or 7 (= *K*) colors. The relative length of the colored segment corresponds to the individual's estimated proportion of membership in that cluster.

We used 33 and 12 populations of *S. angustata* and *S. laevigata*, respectively, with a total of 681 individuals from 33 sites throughout the QTP (Fu et al., 2016; Figure 1). Voucher individuals were deposited in the Qinghai-Tibetan Plateau Museum of Biology, Northwest Institute of Plateau Biology, Chinese Academy of Sciences.

### Amplification and microsatellite genotyping

For this study, we used the 8 microsatellite loci (SS3, SS11, SS16, SS37, SS40, SS43, SS53, and SS54) described by Arranz et al. (2013). PCR reactions were performed in a 15 μl reaction volume containing 10–100 ng of template DNA, 1 × PCR Buffer, 2 mM of MgCl_2_, 0.5 μM of each dNTPs, 0.2 μM of each primer, and 1 U of Taq DNA polymerase (Takara, China). The reaction profile included an initial denaturation at 95°C for 5 min, followed by 36 cycles of initial denaturation at 95°C for 50 s, an annealing temperature of 53°C for 50 s, extension at 72°C for 30 s, and a final extension step at 72°C for 7 min. We analyzed amplified fragments using the QIAxcel DNA High Resolution cartridge and method OM800 on the QIAxcel Advanced System (QIAGEN, Germany). Allele sizing was performed using QIAxcel ScreenGel software version 1.0.2.0 (QIAGEN, Germany) by comparing alleles with a molecular alignment marker (10 and 600 bp, QIAGEN) and size marker (25–500 bp, QIAGEN).

### Genetic variation, Hardy–Weinberg, and linkage equilibrium

The presence of null alleles, scoring errors, and large allele dropout were checked using MICROCHECKER version 2.2.3 (Van Oosterhout et al., 2004). Tests of departure from Hardy-Weinberg equilibrium (HWE) and genotypic linkage disequilibrium (LD) were carried out using Genepop version 4.0.10 (Rousset, 2008). We calculated gene diversity according to Nei (1987), and observed heterozygosity (*H*_*O*_), expected heterozygosity (*H*_*E*_), and pairwise *F*_*ST*_ using ARLEQUIN version 3.5 (Excoffier and Lischer, 2010). Through these methods, we determined the private alleles, i.e., the number of unique alleles in a population relative to the overall number of alleles in that species. Lastly, the fixation index *F*_*IS*_ was determined using FSTAT version 2.9.3.2 (Goudet, 2001) within and across populations.

### Population genetic structure

The genetic structure among populations and individuals was investigated using the Bayesian clustering algorithm implemented in STRUCTURE version 2.3.1 (Pritchard et al., 2000). We ran 10 independent replicates testing for 1–12 clusters (K) using both species' samples. Each run started with a burn-in of 100,000 followed by 1 million iterations. The most likely true value of K was determined using the method proposed by Pritchard et al. (2000) and using the second rate of change in the likelihood distribution (ΔK; Evanno et al., 2005). To develop a consensus value for K, independent runs of all data sets were averaged in CLUMPP v.1.1.2 (Jakobsson and Rosenberg, 2007) using the Greedy algorithm with 10,000 repeats. Graphical representation was performed using DISTRUCT version 1.1 (Rosenberg, 2004). Genetic isolation by distance was determined using IBDWS version 3.23 (Isolation by Distance Web Service, Jensen et al., 2005). Rousset's distance *F*_*ST*_/(1-*F*_*ST*_) was regressed against log-geographical distance and tested for significance using the Mantel test with 10,000 permutations.

### Demographic history

**Figure 2:**
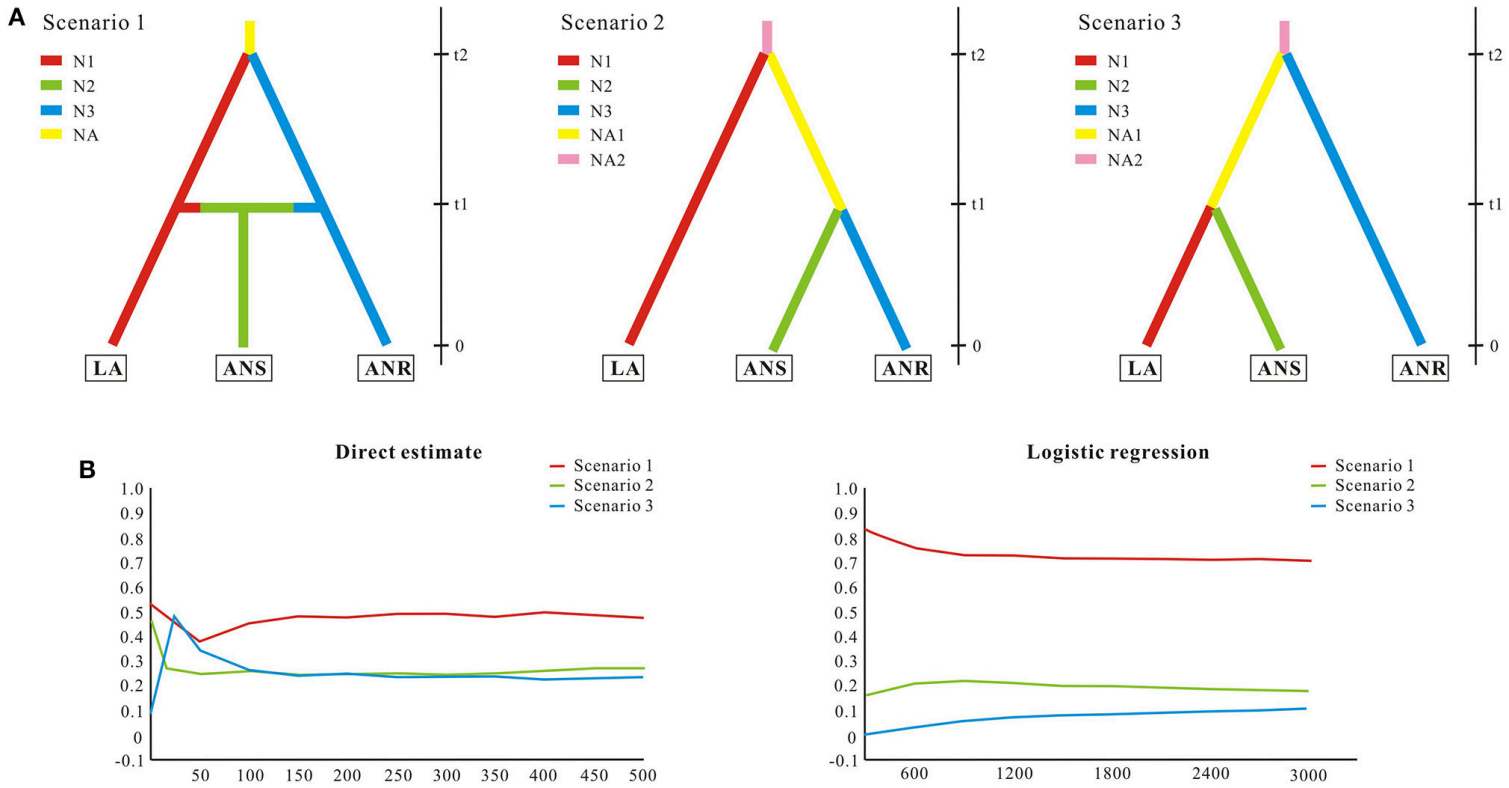
**Possible scenarios of ***Sibiraea*** population history in the Qinghai-Tibetan Plateau**. Approximate Bayesian computation was conducted using DIYABC 2.0.4 to estimate the relative likelihood of scenarios for *Sibiraea*'s evolutionary history. **(A)** Graphs in the upper panel illustrate the proposed scenarios proposed. The *Sibiraea* populations were assumed to be 3 subpopulations. LA contained *S. laevigata* populations; ANS contained *S. angustata* populations that were sympatric with *S. laevigata*; ANR contained the remaining *S. angustata* populations. Colors indicate different (but unknown) population sizes. Zero means sampling time, and t1-t2 refers to relative times of past events that are suggestive of population splitting. **(B)** Graphs in the lower panel indicate the relative likelihoods of the 3 scenarios compared by direct estimate and logistic regression.

To test alternative hypotheses that could explain the genetic similarity between *S. laevigata* and *S. angustata* samples, we conducted an approximate Bayesian computation (ABC) analysis (Beaumont, 2010). The ABC analyses were conducted using DIYABC 2.0 (Cornuet et al., 2014). We assumed populations of *Sibiraea* as 3 subpopulations. The first one (named LA) contained *S. laevigata* populations, the second (named ANS) contained *S. angustata* populations that were sympatric with *S. laevigata*, and the third (named ANR) contained the remaining *S. angustata* populations. A total of 3 evolutionary scenarios were compared (Figure 2). Scenario 1 assumed that an ancestral population of size NA split t2 generations ago into 2 daughter populations, LA and ANR, of effective sizes N1 and N3, respectively; ANS was constituted at t1 generations ago by migrants from LA and ANR at rates r and 1–r, respectively (Figure 2A). In contrast, Scenario 2 assumed that an ancestral population split t2 generations ago into 2 daughter populations, where one was LA and the other split t1 generations ago into ANS and ANR (Figure 2A). Lastly, Scenario 3 is similar to Scenario 2, wherein ANR was first split from the ancestral population t2 generations ago (Figure 2A). The prior distributions were uniform for all parameters, and the same range was used for common parameters between models. For all scenarios, we assumed that microsatellites evolved under a stepwise-mutation model. Under each model, we ran 100,000 coalescent simulations of our data set of 8 microsatellites. The posterior probabilities of the 3 scenarios were subsequently estimated based on (i) a direct estimate, in which the 500 data sets with summary statistics closest to the target values were extracted, and (ii) a polychotomous logistic approach that recovers the 1% closest to the simulated data sets. For the scenario with the highest support, posterior distributions for parameters were estimated using a local linear regression on the closest 1% of the simulated data set. Recent reductions to effective population sizes were tested with the Wilcoxon signed-rank test implemented in BOTTLENECK version 1.2.02 (Cornuet and Luikart, 1996). Tests were conducted using the recommended model in BOTTLENECK, namely the two-phase mutational model (TPM) with 10,000 iterations.

### Migration patterns

We used GENECLASS version 2.0 (Piry et al., 2004) to perform the assignment test with the Bayesian method (Rannala and Mountain, 1997) and detect first-generation migrants among populations using the frequency-based method (Paetkau et al., 1995). The assignment test assigned the possible origins of individuals in reference populations, with 1 million simulations and an alpha level of 0.01. Using Monte Carlo resampling of 1000 individuals and a threshold of 0.01, first-generation migrants among populations were detected to identify immigrant individuals that were not born in the population. Since the migrant rate may not accurately reflect long-term gene flow patterns (Slatkin, 1987), the private allele method was used to estimate the effective number of migrants per generation. This was conducted using Genepop version 4.0.10 (Rousset, 2008). To examine gene flow levels among defined regional groups, we used MIGRATE version 3.6 to estimate theta and M using MCMC searches (Beerli and Felsenstein, 2001; Beerli and Palczewski, 2010). Theta was defined as 4N_e_μ for diploid organisms, where N_e_ is the effective population size and μ is the mutation rate. Conversely, M was expressed as the number of migrants per generation between populations and equal to 4N_e_m, where m is the proportion of the population composed of migrants. We used the stepping-stone model of population structure (Kimura and Weiss, 1964) in our analyses for reducing the number of parameters and degrees of freedom. Therefore, gene flow was only estimated between adjoining regional groups. We performed 20 short chains with 500 sampled genealogies each, and 3 long chains with 5000 sampled genealogies each. Heating was set as active, with four temperatures (1.0, 1.5, 3.0, and 1000.0). This process was repeated 4 times with different random seed numbers, but under the same conditions.

## Results

### Hardy–Weinberg equilibrium, linkage disequilibrium, and genetic diversity

All 681 samples were amplified at all 8 microsatellite loci. Mean expected heterozygosities (*H*_*E*_) were uniformly high, and ranged from 0.564 to 0.895 (Table 1). The mean number of alleles/locations ranged from 4.38 to 15.13. MICROCHECKER detected the presence of null alleles at all loci. Pairwise comparisons among the 8 microsatellite loci and for all 45 populations revealed no linkage disequilibrium (*P* < 0.05). In contrast to *S. laevigata*, more *S. angustata* locations showed significant departure from Hardy-Weinberg equilibrium with a significant multilocus heterozygosity deficiency (Table 1). Furthermore, more private alleles were detected in *S. laevigata* than in *S. angustata*. A private allele was counted following the standard that it appeared only in a single population. The 8 microsatellite markers used in this study were assessed and summarized in Table S1.

**Table 1:** **Summary statistics for ***Sibiraea*** populations in the Qinghai-Tibetan Plateau: sample size (N), average observed heterozygosity over loci (***H***_***O***_), average heterozygosity over loci (***H***_***E***_), number of loci deviating from Hardy-Weinberg equilibrium (HW), number of private alleles (PA), fixation index (***F***_***IS***_)**.

**Population**	**Locality**	**Latitude**	**Longitude**	**N**	**Na**	**Gene diversity**	***H_O_***	***H_E_***	**HW**	**PA**	***F_IS_***
***SIBIRAEA LAEVIGATA***											
MQb	Maqin,QH	34°35′	100°33′	6	6.250	0.820	0.842	0.821	0	1	−0.027
DRb	Dari,QH	33°51′	99°11′	14	11.125	0.885	0.920	0.886	0	2	−0.039
QLb	Qilian,QH	38°15′	99°58′	19	9.750	0.763	0.882	0.766	4	1	−0.155
MYb	Menyuan,QH	37°25′	101°57′	17	11.125	0.814	0.904	0.817	2	2	−0.110
HZb	Huzhu,QH	37°00′	102°10′	4	5.125	0.818	0.875	0.826	0	1	−0.070
PAb	Pingan,QH	36°17′	101°59′	16	10.875	0.822	0.930	0.825	1	0	−0.131
XHb	Xiahe,GS	34°45′	102°35′	17	11.625	0.829	0.919	0.832	2	3	−0.109
REG1b	Ruoergai,SC	34°07′	102°39′	12	10.625	0.844	0.885	0.846	0	1	−0.049
REG2b	Ruoergai,SC	33°41′	103°28′	4	4.750	0.828	0.844	0.830	0	1	−0.019
REG3b	Ruoergai,SC	33°25′	102°33′	8	8.750	0.868	0.906	0.871	0	0	−0.044
BMb	Banma,QH	33°03′	100°34′	4	4.750	0.807	0.938	0.826	0	0	−0.161
YSb	Yushu,QH	33°12′	97°28′	9	8.625	0.855	0.875	0.856	0	1	−0.023
	Subtotal				8.615	0.829	0.893	0.834	0.75	1.08	−0.078
***SIBIRAEA ANGUSTATA***											
MQa	Maqin,QH	34°35′	100°33′	15	9.750	0.825	0.867	0.826	2	2	−0.051
DR1	Dari,QH	33°19′	100°28′	9	6.875	0.797	0.847	0.800	0	0	−0.063
DR2a	Dari,QH	33°51′	99°11′	12	9.625	0.853	0.813	0.851	0	0	0.047
QLa	Qilian,QH	38°15′	99°58′	15	9.250	0.735	0.742	0.735	2	1	−0.009
MYa	Menyuan,QH	37°25′	101°57′	25	11.125	0.790	0.785	0.790	4	0	0.007
HZa	Huzhu,QH	37°00′	102°09′	7	6.625	0.753	0.821	0.758	0	0	−0.091
PAa	Pingan,QH	36°17′	101°59′	21	12.000	0.824	0.798	0.823	3	0	0.031
XHa	Xiahe,GS	34°45′	102°35′	18	12.125	0.817	0.813	0.816	1	0	0.005
REG1a	Ruoergai,SC	34°07′	102°39′	9	8.250	0.820	0.847	0.821	0	1	−0.034
REG2a	Ruoergai,SC	33°41′	103°28′	11	11.125	0.864	0.886	0.865	0	1	−0.026
REG3a	Ruoergai,SC	33°25′	102°33′	15	11.875	0.842	0.758	0.839	2	0	0.100
HY	Hongyuan,SC	32°21′	102°27′	14	11.625	0.847	0.741	0.843	1	1	0.125
DQIN	Deqin,YN	28°23′	98°59′	25	11.000	0.756	0.680	0.754	5	3	0.100
DC	Daocheng,SC	29°08′	100°02′	29	13.125	0.852	0.764	0.850	5	1	0.103
LT	Litang,SC	29°32′	100°17′	22	12.250	0.851	0.818	0.850	2	1	0.038
GZ	Ganzi,SC	31°36′	100°10′	24	12.125	0.834	0.839	0.834	4	0	−0.005
DF	Daofu,SC	30°52′	101°15′	19	10.875	0.835	0.740	0.833	3	1	0.114
DB	Danba,SC	30°32′	101°35′	16	11.750	0.868	0.711	0.863	2	1	0.181
LH	Luohu,SC	31°37′	100°43′	14	10.125	0.843	0.723	0.838	3	0	0.142
AB	Aba,SC	32°43′	102°10′	12	8.625	0.824	0.656	0.817	2	0	0.203
BMa	Jiuzhi,QH	33°20′	101°30′	16	10.500	0.830	0.757	0.827	2	0	0.087
YS1a	Yushu,QH	33°12′	97°28′	18	11.625	0.840	0.729	0.837	3	0	0.132
YS2	Yushu,QH	32°46′	97°12′	17	11.000	0.858	0.710	0.854	3	0	0.173
YS3	Yushu,QH	32°33′	96°29′	10	8.125	0.833	0.675	0.824	4	0	0.189
NQ	Nangqian,QH	31°58′	96°32′	19	11.500	0.831	0.757	0.829	3	1	0.089
YS4	Yushu,QH	32°00′	96°20′	12	9.750	0.867	0.792	0.864	3	0	0.087
LWQ1	Leiwuqi,T	31°32′	96°22′	16	10.750	0.830	0.789	0.829	4	0	0.049
LWQ2	Leiwuqi,T	31°06′	96°20′	20	12.000	0.841	0.825	0.841	1	0	0.019
DQ	Dingqing,T	31°32′	95°19′	15	10.875	0.843	0.692	0.837	2	0	0.179
BQ	Baqing,T	31°47′	94°30′	24	15.125	0.895	0.823	0.893	5	1	0.080
CD	Changdu,T	31°18′	97°30′	24	14.750	0.868	0.823	0.867	5	0	0.052
JD	Jiangda,T	31°38′	98°26′	14	11.750	0.897	0.839	0.895	3	1	0.065
DG	Dege,SC	31°53′	99°01′	14	11.500	0.852	0.893	0.854	1	0	−0.048
	Subtotal				10.890	0.834	0.780	0.832	2.42	0.48	0.063

*Abbreviations after localities indicate provinces as follows: QH, Qinghai; SC, Sichuan; T, Tibet; YN, Yunnan; GS, Gansu*.

Recent inbreeding was apparent for all *S. laevigata* populations but only for a few *S. angustata* populations (8 out of 33). Populations in the sympatric area of the complex were more inbred than those from the non-sympatric area. Pairwise *F*_*ST*_ values (Table S2) clearly indicate significant genetic differentiation between populations. Levels of pairwise population differentiation were variable, ranging from 0.001 to 0.283. The pairwise *F*_*ST*_ values between *S. laevigata* and *S. angustata* was 0.049 (*P* = 0.000). AMOVA analysis showed that 4.39% of the variance was found between species (Va = 0.166), 8.16% among populations within species (Vb = 0.309), and 87.45% within populations (Vc = 3.315) (Table 2).

**Table 2:** **Analyses of molecular variance (AMOVA) in ***Sibiraea*** based on 8 SSR loci**.

**Source of variation**	**d.f**.	**SS**	**VC**	**PV**
Among species	1	82.297	0.166 Va	4.39
Among populations in species	43	544.107	0.309 Vb	8.16
Within populations	1317	4365.803	3.315 Vc	87.45

*d.f., degrees of freedom; SS, Sum of squares; VC, Variance components; PV, Percentage of variation*.

### Population structure

In Bayesian clustering analysis, the Ln-likelihood values for the number of clusters (*K*) increased with each *K*-value and did not plateau when the complex was analyzed together (Figure S1). Using the method proposed by Evanno et al. (2005) the suggested number of clusters were 2 and 7 (Δ*K* = 2 and 7, Figure S1). When examining bar-plot outputs during analyses for the *K*-values, results were biologically meaningful. We therefore consider *K* = 2 and *K* = 7 to be the most likely number of clusters in the 2 *Sibiraea* species. At *K* = 2, all *S. laevigata* individuals were primarily located in 1 cluster, while *S. angustata* individuals were in 2 clusters (Figure 1C). The individual's estimated proportion of membership in each cluster is presented in Table S3. The first cluster contained all populations at the sympatric area of the complex. The genetic structure of *Sibiraea* populations based on *K* = 2 genetic clusters was presented in Figure 1B. At higher *K*-values, individuals within the *S. angustata* subpopulation were assigned to additional clusters. At *K* = 7, *S. laevigata* individuals were assigned to 2 clusters, while *S. angustata* individuals were assigned to 6 clusters, one of which was shared with *S. laevigata* (Figure 1C).

### Demographic history

Since all populations at the sympatric area were grouped into a single cluster, we conducted ABC analysis to reveal the divergence history of populations at this area. The ABC simulations showed high support for Scenario 1 (subpopulation ANS was constituted by migrants from subpopulations LA and ANR) and favored this as the most probable scenario (Figure 2). The median estimates of migrant rate r, split time t1 and t2, as estimated from posterior distributions, were around 0.61, 4600, and 34,200, respectively (Table S4). Despite some of the estimated parameters showing low robustness (Factor 2 statistic, Table S5), ABC analyses conclusively support a migrant in the *Sibiraea* species populations. Posteriors of the effective population sizes, rate of admixture, and time of population splits are given in Table S4. Due to an excess of observed heterozygosity, bottleneck tests under TPM models showed deviations from mutation equilibrium for populations P9, P11, and S32. Two other populations, S13 and S22, deviated from mutation equilibrium due to deficiency of observed heterozygosity and experienced genetic bottleneck (Table S6).

### Gene flow and migration

There were no significant correlations between genetic and geographical distances among the populations analyzed in this study (for *S. laevigata, r* = 0.457, *P* = 0.997; for *S. angustata, r* = 0.236, *P* = 1.000). The assignment test assigned all individuals to populations of their own species rather than their congeneric species. GENEGLASS detected no first-generation migrant between the 2 species. Ten first-generation migrants were detected within *S. laevigata* populations and 41 first-generation migrants were detected within *S. angustata* populations (Table S7). Based on Genepop's private allele method, the number of migrants (after correction for size) in *S. laevigata, S. angustata*, and the *Sibiraea* complex were 2.33, 4.88, and 2.75, respectively. Results from MIGRATE showed that recent immigration rate from *S. laevigata* to *S. angustata* (m_12_ = 0.0059) was much lower than the recent immigration rate from *S. angustata* to *S. laevigata* (m_21_ = 0.0234) (Table 3). This suggests that the bidirectional gene flow between the species was asymmetrical. Focusing on the sympatric area, recent immigration rate from *S. laevigata* to *S. angustata* was only slightly lower (m_12_ = 0.0106) than that from *S. angustata* to *S. laevigata* (m_21_ = 0.0187).

**Table 3:** **Effective population sizes (***N***_***e***_) and effective number of migrants between species (4***N***_***e***_m) in ***S. angustata*** and ***S. laevigata***, estimated by maximum likelihood**.

**Species**	**θ**	**M_21_**	**M_12_**	**m_21_**	**m_12_**	***N_e_***	**4*N_e_*m_21_**	**4*N_e_*m_12_**
**WHOLE AREA**								
*S. laevigata*	0.6365	–	5.95	–	5.95 × 10^−3^	159.13	–	3.79
*S. angustata*	2.3218	22.36	–	2.34 × 10^−2^	–	580.45	51.92	–
**SYMPATRIC AREA**								
*S. laevigata*	0.699	–	10.642	–	1.06 × 10^−2^	174.8	–	7.44
*S. angustata*	1.7668	18.733	–	1.87 × 10^−2^	–	441.7	33.1	–

θ, 4N_e_μ; N_e_, effective population size; μ, mutation rate (μ = 10^−3^ per gamete per year); M, m/μ; 4N_e_m, 4^*^(θ/4μ)^*^Mμ = θM, the effective number of migrants; 4N_e_m_12_, the effective number of migrants from S. laevigata to S. angustata per generation (5 years); 4N_e_m_21_, the effective number of migrants from S. angustata to S. laevigata per generation (5 years).

## Discussion

The *Sibiraea* complex, *S. angustata*, and *S. laevigata*, is a common shrub in the QTP. Previous study based on cpDNA and ITS indicated that the 2 species had high genetic similarity and could not be genetically distinguished (Fu et al., 2016). However, by including data from microsatellite loci, a more detailed genetic structure can be established, and this approach has been successfully applied to a range of species (Kalia et al., 2011).

### Population genetic structure

The challenge in molecular taxonomy is to distinguish species that have low levels of genetic divergence either due to recent speciation or continuous gene exchange (Petit and Excoffier, 2009). Although, the *Sibiraea* complex could not be distinguished by cpDNA and nrITS datasets, it may be distinguished using microsatellite loci. Firstly, *S. laevigata* and *S. angustata* had significant differentiation (*F*_*ST*_ = 0.049, *P* = 0.000). Then, STRUCTURE provided a comprehensive understanding of the *Sibiraea* populations under study. The *K* = 2 model seems to support the interpretation that each cluster represents a single geographical region; one is the sympatric region, while the other is the left region. The *K* = 7 model in STRUCTURE clearly separated *S. laevigata* populations from *S. angustata* populations. Furthermore, STRUCTURE grouped all *S. laevigata* populations into a unique cluster (the blue one), and *S. angustata* populations under another clusters (Figure 1C).

Even though the complex was genetically distinguished, they shared some genetic variability. There was evident structuring regarding 5 populations (P8–P12), where approximately 60% of genetic variability was attributed to the blue cluster, and 40% to the red cluster (Figure 1C), which also contained 1 *S. angustata* population (S13). However, population S13 was not geographically adjacent to the 5 populations. Since a significant bottleneck effect was detected in population S13, the geographical disjunction between population S13 and other populations of the red cluster might be due to a genetic remnant following the genetic bottleneck.

Hartl et al. (1997) proposed *F*_*ST*_ < 0.05 as indicator of little genetic differentiation. The *F*_*ST*_ between the *Sibiraea* complex is low (0.049), meaning that there is not much genetic differentiation between them. At the same time, shared genetic variation was observed and most genetic variance was detected within populations rather than between species. However, the complex has been divergent in stable morphological characteristics, such as pubescent or peduncles and pedicels (Gu and Alexander, 2003). Therefore, the *Sibiraea* complex may be on an incomplete process of speciation. Ongoing speciation has been reported in several taxa such as the *Gymnocypris* complex in the QTP (Zhang et al., 2013) and the *Anastrepha fraterculus* complex in the Americas (Devescovi et al., 2014).

### Gene flow in *Sibiraea* complex

High genetic diversity was detected in both species (Table 1). On one hand, the high genetic diversity owns to high polymorphism of SSR loci used in this study because SSR loci have higher mutation rates than chloroplasts and nuclear genes. On the other hand, our results indicated that both species had high genetic diversity. Geographical isolation could increase opportunities for genetic divergence. Vicariance on the QTP, and fragmentation during repeated glaciations in the late Pleistocene, contributed to plant evolution and differentiation (Liu et al., 2012, 2014; Wen et al., 2014). Therefore, geographical isolation due to vicariance and fragmentation may partly contribute to this high genetic diversity.

While genetic diversity increases, geographical isolation simultaneously intensifies genetic differentiation. Our results showed no significant correlations between genetic and geographical distances among the populations, therefore indicating that *Sibiraea* was not isolated by distance. Gene flow between populations maintains genetic diversity within a species (Slatkin, 1994), and frequent gene flow increases genetic diversity. However, as genetic diversity increases, gene flow decreases genetic differentiation. Therefore, logically, if gene flow increased genetic diversity, a decrease in genetic differentiation should be observed. In our study, a low differentiation was confirmed by low *F*_*ST*_ (0.049) between the *Sibiraea* complex. Coincidentally, AMOVA analysis also indicated that the majority of diversity was within populations (87.45%) rather than among populations (12.55%). Moreover, MCMC searches indicated high gene flow between *S. laevigata* and *S. angustata*, especially on the sympatric area (Table 3). Although, the QTP is mountainous, frequent gene flow is possible because both species are wind-pollinated, exhibit the same flowering times, and have a number of light seeds with wings (Fu et al., 2016). Genetic differentiation despite high gene flow was also detected in the wind-pollinated keystone rainforest tree *Nothofagus cunninghamii* (Duncan et al., 2016). Our study agrees with the statement that speciation with gene flow may be common (Nosil, 2008).

### Evolutionary history

In addition to distribution in the QTP, *S. laevigata* also presently distributes in the Mediterranean (Gu and Alexander, 2003; Ballian et al., 2006), and may have distributed around the Tethys Ocean before the QTP uplift. On the other hand, *S. angustata* was endemic to the QTP. Although no genetic differentiation was observed, the Bayesian estimation of the divergence time in the *Sibiraea* complex in the QTP was around 3.8 Ma (Fu et al., 2016), when the QTP quickly uplifted (Shi et al., 1998). Our ABC model based on microsatellites indicated that populations of the 2 species split around 0.17 Ma, when glaciers occurred in the QTP (Zheng et al., 2002). According to these results, *S. angustata* possibly originated from the remnants of *S. laevigata* in the QTP, which diverged with the uplift and glacier formation. Having hair in its stems and leaves, *S. angustata* adapted to the cold environment of the QTP and had wider distributions than *S. laevigata*.

Studying closely related species and divergent populations gives insight into how genetic differences in speciation proceed (Surget-Groba and Kay, 2013). The Bayesian clustering analysis tended to cluster the populations of the complex at the sympatric area into 1 group. Our ABC model supported that the populations of *S. angustata* at the sympatric area was an admixture between the *S. laevigata* populations and southern *S. angustata* populations. *Sibiraea laevigata* (61%) contributed to the admixture more than the southern *S. angustata* (39%). Significant bidirectional gene flow between them, especially on the sympatric area, contributed to their high genetic similarity. When generation time of *Sibiraea* was set to 5 years, according to our field observations, the admixture occurred about 23,000 years ago, i.e., when the QTP was in the Last Glacial period. Phylogeographic study indicated that the *Sibiraea* complex had 3 independent refugia in the QTP, 2 of which were high altitude (Fu et al., 2016). Population glacial contractions and postglacial recolonization offered opportunities for population admixture (Hewitt, 2000). The ABC model also indicated that *S. laevigata* populations and southern *S. angustata* populations split around 0.17 Ma. During this period (the penultimate glacial), the area covered by glaciers was larger than that of former glaciations in the southern part of Himalaya and Southeastern Tibet (Zheng et al., 2002). This larger glacier possibly triggered *Sibiraea* divergence and decreased population size. The population size decrease was observed in our previous study using the Bayesian skyline plot, which showed that *Sibiraea* populations in the QTP decreased 23-fold during the last 0.12 Ma (Fu et al., 2016).

In conclusion, this study examined the population genetic structure of 45 populations of *Sibiraea* from the QTP. The *Sibiraea* complex, *S. angustata* and *S. laevigata*, which could not be distinguished by DNA sequence data in previous research, were genetically distinguished based on the microsatellite markers applied in this study. We found that the high genetic similarity of these 2 species is the result of huge bidirectional gene flow, especially on the sympatric area where population admixtures occurred. The *Sibiraea* complex may be on an incomplete process of speciation with gene flow. Our study suggests that microsatellite loci are helpful for species population genetics, especially for closely related species. This study greatly enhanced our knowledge on the genetic diversity and evolutionary history of *Sibiraea* in the QTP.

## Author contributions

PF conceived and designed the experiments, performed the experiments, analyzed the data, wrote the paper, prepared figures and/or tables. QG reviewed drafts of the paper, revise the manuscript. FZ analyzed the data, prepared figures and/or tables, reviewed drafts of the paper, revise the manuscript. RX, JW, and HL performed the experiments, analyzed the data. SC conceived and designed the experiments, reviewed drafts of the paper. PF, FZ have contributed equally to this work.

### Conflict of interest statement

The authors declare that the research was conducted in the absence of any commercial or financial relationships that could be construed as a potential conflict of interest.
