# Supplementary material for: Gene Flow Results in High Genetic Similarity between Sibiraea (Rosaceae) Species in the Qinghai-Tibetan Plateau
Source: Front Plant Sci. 2016 Oct 25;7:1596. doi: 10.3389/fpls.2016.01596 (PMC5078775; doi:10.3389/fpls.2016.01596)
Supplement: Supplementary file 1 [file Table1.DOCX]

Table S1. Summary statistics for eight microsatellite markers based on *Sibiraea* populations on the Qinghai-Tibetan Plateau: total number of observed allele (Nt), average observed heterozygosity (*H_O_*), average heterozygosity (*H_E_*), number of population deviating from Hardy-Weinberg equilibrium (HW), number of private alleles (PA), fixation index (*F_IS_*).

|  | Locus | Size | Nt | Gene | *H_O_* | *H_E_* | HW | PA | *F_IS_* |
| --- | --- | --- | --- | --- | --- | --- | --- | --- | --- |
| *S.laevigata* | SS3 | 76-106 | 9 | 0.622 | 0.515 | 0.640 | 1 | 2 | 0.094 |
|  | SS11 | 128-180 | 27 | 0.860 | 0.946 | 0.939 | 1 | 2 | 0.051 |
|  | SS16 | 123-197 | 30 | 0.924 | 0.969 | 0.947 | 0 | 6 | 0.029 |
|  | SS37 | 121-173 | 22 | 0.902 | 0.853 | 0.924 | 1 | 5 | 0.032 |
|  | SS40 | 108-184 | 30 | 0.936 | 0.992 | 0.955 | 0 | 6 | 0.016 |
|  | SS43 | 177-245 | 30 | 0.902 | 0.938 | 0.931 | 2 | 11 | 0.027 |
|  | SS53 | 127-191 | 23 | 0.733 | 0.985 | 0.866 | 4 | 7 | 0.203 |
|  | SS54 | 124-160 | 18 | 0.756 | 1.000 | 0.851 | 0 | 3 | 0.125 |
|  | Mean |  | 23.63 | 0.829 | 0.900 | 0.882 | 1.13 | 5.25 | 0.072 |
| *S.angustata* | SS3 | 74-110 | 15 | 0.602 | 0.615 | 0.769 | 7 | 3 | 0.224 |
|  | SS11 | 106-172 | 30 | 0.877 | 0.773 | 0.935 | 10 | 4 | 0.066 |
|  | SS16 | 123-189 | 32 | 0.912 | 0.924 | 0.936 | 5 | 4 | 0.027 |
|  | SS37 | 111-493 | 37 | 0.845 | 0.599 | 0.916 | 20 | 7 | 0.077 |
|  | SS40 | 100-178 | 39 | 0.934 | 0.783 | 0.961 | 16 | 4 | 0.028 |
|  | SS43 | 167-269 | 47 | 0.902 | 0.873 | 0.954 | 7 | 4 | 0.056 |
|  | SS53 | 113-197 | 35 | 0.833 | 0.746 | 0.935 | 9 | 3 | 0.110 |
|  | SS54 | 110-158 | 25 | 0.765 | 0.920 | 0.894 | 6 | 1 | 0.142 |
|  | Mean |  | 32.5 | 0.834 | 0.779 | 0.913 | 10.00 | 3.75 | 0.091 |
