# Supplementary material for: Gene Flow Results in High Genetic Similarity between Sibiraea (Rosaceae) Species in the Qinghai-Tibetan Plateau
Source: Front Plant Sci. 2016 Oct 25;7:1596. doi: 10.3389/fpls.2016.01596 (PMC5078775; doi:10.3389/fpls.2016.01596)
Supplement: Supplementary file 2 [file Table2.DOCX]

Table S2. Genetic differentiation between *Sibiraea* populations on the Qinghai-Tibetan Plateau. Pairwise *F_ST_* is presented above the diagonal with corresponding Euclidean geographical distances (km) between sites presented below the diagonal. Significant P-values are indicated in bold (P < 0.05).

| Site | MQ | DR1 | DR2 | QL | MY | HZ | PA | XH | REG1a | REG2a | REG3a | HY | DQINa | DC | LT | GZ | DF | DB | LH | AB | JZ | YS1 | YS2 | YS3 | NQ | YS4 | LWQ1a | LWQ2a | DQ | BQ | CD | JD | DG | ZD | MZGKa | GBJDa | WY | JZG | CZS | XJ | RT | BM |
| --- | --- | --- | --- | --- | --- | --- | --- | --- | --- | --- | --- | --- | --- | --- | --- | --- | --- | --- | --- | --- | --- | --- | --- | --- | --- | --- | --- | --- | --- | --- | --- | --- | --- | --- | --- | --- | --- | --- | --- | --- | --- | --- |
| MQ |  | **0.082** | **0.102** | **0.086** | **0.073** | **0.077** | **0.104** | **0.111** | **0.065** | **0.072** | **0.047** | **0.066** | **0.173** | **0.243** | **0.279** | **0.191** | **0.088** | **0.091** | **0.122** | **0.093** | **0.182** | **0.148** | **0.173** | **0.140** | **0.146** | **0.132** | **0.112** | **0.084** | **0.079** | **0.191** | **0.138** | **0.075** | **0.046** | **0.095** | **0.071** | **0.095** | **0.076** | **0.149** | **0.134** | **0.124** | **0.094** | **0.165** |
| DR1 | 26 |  | 0.003 | 0.010 | -0.001 | 0.010 | **0.030** | **0.051** | **0.054** | **0.083** | **0.041** | **0.095** | **0.143** | **0.222** | **0.243** | **0.146** | **0.085** | **0.091** | **0.105** | **0.085** | **0.137** | **0.116** | **0.124** | **0.107** | **0.108** | **0.085** | **0.076** | **0.055** | **0.032** | **0.174** | **0.135** | **0.047** | **0.082** | **0.084** | **0.034** | **0.023** | **0.017** | **0.137** | **0.109** | **0.118** | **0.045** | **0.148** |
| DR2 | 137 | 136 |  | 0.020 | -0.002 | 0.013 | **0.028** | **0.038** | **0.064** | **0.095** | **0.047** | **0.100** | **0.161** | **0.247** | **0.276** | **0.168** | **0.090** | **0.097** | **0.124** | **0.101** | **0.162** | **0.145** | **0.155** | **0.128** | **0.136** | **0.120** | **0.104** | **0.087** | **0.048** | **0.197** | **0.148** | **0.076** | **0.096** | **0.103** | **0.049** | **0.040** | **0.046** | **0.156** | **0.127** | **0.133** | **0.044** | **0.171** |
| QL | 112 | 130 | 98 |  | 0.004 | **0.016** | **0.018** | **0.051** | **0.065** | **0.092** | **0.045** | **0.106** | **0.162** | **0.234** | **0.241** | **0.152** | **0.097** | **0.096** | **0.121** | **0.096** | **0.151** | **0.130** | **0.136** | **0.123** | **0.125** | **0.097** | **0.101** | **0.086** | **0.033** | **0.196** | **0.147** | **0.075** | **0.094** | **0.088** | **0.046** | **0.031** | **0.027** | **0.154** | **0.125** | **0.128** | **0.048** | **0.153** |
| MY | 151 | 164 | 284 | 223 |  | 0.009 | **0.021** | **0.038** | **0.044** | **0.076** | **0.034** | **0.088** | **0.146** | **0.226** | **0.246** | **0.144** | **0.075** | **0.081** | **0.099** | **0.077** | **0.141** | **0.124** | **0.128** | **0.104** | **0.111** | **0.087** | **0.077** | **0.064** | **0.022** | **0.173** | **0.124** | **0.053** | **0.075** | **0.086** | **0.025** | **0.022** | **0.027** | **0.137** | **0.113** | **0.113** | **0.038** | **0.143** |
| HZ | 204 | 214 | 339 | 282 | 58 |  | **0.042** | **0.055** | **0.061** | **0.097** | **0.059** | **0.105** | **0.157** | **0.223** | **0.255** | **0.163** | **0.093** | **0.106** | **0.131** | **0.115** | **0.167** | **0.141** | **0.147** | **0.134** | **0.127** | **0.109** | **0.112** | **0.087** | **0.021** | **0.182** | **0.141** | **0.071** | **0.095** | **0.093** | **0.050** | **0.047** | **0.033** | **0.151** | **0.126** | **0.130** | **0.055** | **0.160** |
| PA | 145 | 154 | 280 | 227 | 24 | 60 |  | **0.029** | **0.061** | **0.093** | **0.052** | **0.108** | **0.152** | **0.234** | **0.247** | **0.124** | **0.096** | **0.090** | **0.103** | **0.073** | **0.121** | **0.117** | **0.119** | **0.100** | **0.105** | **0.100** | **0.089** | **0.089** | **0.061** | **0.188** | **0.144** | **0.083** | **0.104** | **0.100** | **0.068** | **0.051** | **0.053** | **0.146** | **0.121** | **0.124** | **0.051** | **0.152** |
| XH | 224 | 226 | 360 | 318 | 107 | 66 | 93 |  | **0.057** | **0.101** | **0.066** | **0.109** | **0.151** | **0.233** | **0.263** | **0.143** | **0.097** | **0.077** | **0.108** | **0.084** | **0.138** | **0.126** | **0.131** | **0.103** | **0.108** | **0.089** | **0.088** | **0.083** | **0.084** | **0.189** | **0.141** | **0.081** | **0.099** | **0.092** | **0.085** | **0.076** | **0.074** | **0.140** | **0.128** | **0.132** | **0.065** | **0.165** |
| REG1a | 229 | 229 | 364 | 324 | 116 | 76 | 102 | 10 |  | **0.025** | **0.014** | **0.028** | **0.097** | **0.188** | **0.208** | **0.131** | **0.069** | **0.066** | **0.095** | **0.069** | **0.143** | **0.117** | **0.129** | **0.102** | **0.112** | **0.098** | **0.082** | **0.079** | **0.065** | **0.130** | **0.087** | **0.072** | **0.062** | **0.059** | **0.054** | **0.073** | **0.073** | **0.083** | **0.073** | **0.074** | **0.053** | **0.115** |
| REG2a | 328 | 327 | 463 | 425 | 211 | 158 | 200 | 107 | 100 |  | 0.006 | 0.002 | **0.125** | **0.206** | **0.229** | **0.149** | **0.073** | **0.081** | **0.107** | **0.085** | **0.162** | **0.129** | **0.144** | **0.117** | **0.127** | **0.123** | **0.093** | **0.091** | **0.088** | **0.144** | **0.110** | **0.079** | **0.058** | **0.073** | **0.076** | **0.085** | **0.083** | **0.115** | **0.095** | **0.099** | **0.076** | **0.142** |
| REG3a | 224 | 222 | 358 | 324 | 125 | 92 | 107 | 29 | 21 | 105 |  | 0.011 | **0.113** | **0.196** | **0.221** | **0.132** | **0.061** | **0.059** | **0.091** | **0.062** | **0.142** | **0.111** | **0.121** | **0.102** | **0.112** | **0.094** | **0.074** | **0.065** | **0.057** | **0.141** | **0.094** | **0.057** | **0.039** | **0.057** | **0.047** | **0.052** | **0.056** | **0.107** | **0.083** | **0.084** | **0.042** | **0.122** |
| HY | 220 | 215 | 352 | 324 | 139 | 114 | 118 | 54 | 46 | 116 | 26 |  | **0.135** | **0.212** | **0.235** | **0.163** | **0.086** | **0.084** | **0.116** | **0.090** | **0.178** | **0.142** | **0.152** | **0.126** | **0.144** | **0.134** | **0.104** | **0.101** | **0.095** | **0.164** | **0.107** | **0.088** | **0.060** | **0.087** | **0.073** | **0.110** | **0.098** | **0.120** | **0.099** | **0.103** | **0.089** | **0.138** |
| DQINa | 223 | 213 | 107 | 205 | 374 | 426 | 366 | 435 | 438 | 532 | 428 | 416 |  | **0.181** | **0.217** | **0.205** | **0.145** | **0.133** | **0.154** | **0.133** | **0.206** | **0.168** | **0.188** | **0.161** | **0.161** | **0.167** | **0.140** | **0.152** | **0.163** | **0.139** | **0.062** | **0.153** | **0.127** | **0.136** | **0.163** | **0.172** | **0.162** | **0.060** | 0.023 | **0.061** | **0.141** | **0.083** |
| DC | 109 | 85 | 130 | 179 | 242 | 285 | 228 | 283 | 284 | 375 | 272 | 259 | 159 |  | **0.057** | **0.244** | **0.207** | **0.207** | **0.241** | **0.222** | **0.274** | **0.228** | **0.274** | **0.236** | **0.247** | **0.266** | **0.233** | **0.220** | **0.232** | **0.237** | **0.167** | **0.210** | **0.216** | **0.222** | **0.241** | **0.263** | **0.238** | **0.171** | **0.155** | **0.187** | **0.228** | **0.183** |
| LT | 101 | 76 | 140 | 180 | 228 | 270 | 213 | 267 | 268 | 358 | 256 | 242 | 176 | 16 |  | **0.279** | **0.236** | **0.228** | **0.262** | **0.246** | **0.305** | **0.252** | **0.308** | **0.264** | **0.274** | **0.287** | **0.258** | **0.252** | **0.259** | **0.283** | **0.209** | **0.243** | **0.246** | **0.249** | **0.250** | **0.283** | **0.253** | **0.210** | **0.163** | **0.196** | **0.257** | **0.214** |
| GZ | 65 | 42 | 116 | 141 | 205 | 253 | 194 | 259 | 262 | 357 | 252 | 242 | 176 | 45 | 41 |  | **0.113** | **0.109** | **0.113** | **0.105** | **0.126** | **0.112** | **0.115** | **0.086** | **0.109** | **0.136** | **0.099** | **0.138** | **0.167** | **0.229** | **0.177** | **0.147** | **0.170** | **0.125** | **0.138** | **0.163** | **0.171** | **0.188** | **0.179** | **0.179** | **0.101** | **0.206** |
| DF | 120 | 104 | 233 | 232 | 155 | 179 | 133 | 160 | 160 | 247 | 146 | 130 | 287 | 128 | 112 | 118 |  | **0.037** | **0.098** | **0.085** | **0.141** | **0.121** | **0.126** | **0.106** | **0.126** | **0.122** | **0.090** | **0.105** | **0.097** | **0.168** | **0.122** | **0.098** | **0.070** | **0.052** | **0.067** | **0.086** | **0.102** | **0.137** | **0.120** | **0.124** | **0.055** | **0.146** |
| DB | 141 | 127 | 257 | 253 | 155 | 172 | 132 | 145 | 143 | 226 | 128 | 110 | 310 | 150 | 134 | 142 | 24 |  | **0.087** | **0.076** | **0.137** | **0.112** | **0.107** | **0.100** | **0.112** | **0.098** | **0.079** | **0.090** | **0.111** | **0.163** | **0.121** | **0.094** | **0.048** | **0.048** | **0.074** | **0.092** | **0.103** | **0.125** | **0.110** | **0.115** | **0.062** | **0.144** |
| LH | 61 | 35 | 152 | 162 | 178 | 222 | 164 | 223 | 225 | 320 | 215 | 205 | 212 | 64 | 51 | 37 | 81 | 105 |  | **0.023** | **0.024** | **0.009** | **0.041** | **0.018** | **0.053** | **0.089** | **0.067** | **0.103** | **0.111** | **0.165** | **0.128** | **0.117** | **0.107** | **0.104** | **0.102** | **0.124** | **0.113** | **0.138** | **0.124** | **0.122** | **0.089** | **0.160** |
| AB | 200 | 196 | 333 | 305 | 124 | 107 | 103 | 55 | 51 | 134 | 33 | 20 | 398 | 241 | 225 | 224 | 114 | 95 | 186 |  | **0.042** | **0.044** | **0.069** | **0.029** | **0.075** | **0.096** | **0.060** | **0.089** | **0.102** | **0.158** | **0.103** | **0.095** | **0.088** | **0.090** | **0.101** | **0.101** | **0.094** | **0.110** | **0.100** | **0.097** | **0.078** | **0.130** |
| JZ | 110 | 107 | 243 | 216 | 95 | 124 | 73 | 120 | 123 | 221 | 115 | 110 | 315 | 165 | 149 | 139 | 60 | 63 | 103 | 91 |  | **0.017** | **0.049** | **0.029** | **0.071** | **0.140** | **0.112** | **0.144** | **0.165** | **0.212** | **0.175** | **0.164** | **0.168** | **0.143** | **0.162** | **0.167** | **0.153** | **0.188** | **0.180** | **0.182** | **0.126** | **0.204** |
| YS1 | 340 | 340 | 204 | 268 | 483 | 540 | 482 | 564 | 568 | 667 | 562 | 555 | 164 | 312 | 327 | 315 | 432 | 456 | 352 | 536 | 447 |  | **0.034** | **0.021** | **0.058** | **0.095** | **0.090** | **0.120** | **0.133** | **0.171** | **0.142** | **0.142** | **0.128** | **0.110** | **0.135** | **0.151** | **0.122** | **0.151** | **0.141** | **0.141** | **0.108** | **0.172** |
| YS2 | 359 | 358 | 222 | 288 | 503 | 559 | 501 | 582 | 587 | 685 | 580 | 573 | 176 | 327 | 342 | 331 | 449 | 472 | 369 | 554 | 465 | 20 |  | **0.024** | **0.076** | **0.100** | **0.109** | **0.142** | **0.145** | **0.206** | **0.158** | **0.157** | **0.146** | **0.124** | **0.133** | **0.163** | **0.134** | **0.173** | **0.156** | **0.156** | **0.107** | **0.186** |
| YS3 | 451 | 450 | 315 | 376 | 594 | 651 | 593 | 675 | 679 | 778 | 673 | 665 | 263 | 418 | 434 | 424 | 541 | 565 | 461 | 646 | 557 | 111 | 93 |  | **0.024** | **0.061** | **0.045** | **0.090** | **0.110** | **0.171** | **0.120** | **0.114** | **0.123** | **0.104** | **0.111** | **0.128** | **0.110** | **0.146** | **0.136** | **0.136** | **0.084** | **0.161** |
| NQ | 449 | 448 | 313 | 376 | 593 | 650 | 591 | 673 | 677 | 775 | 670 | 662 | 258 | 414 | 430 | 421 | 538 | 561 | 458 | 644 | 555 | 109 | 91 | 10 |  | **0.026** | **0.019** | **0.058** | **0.107** | **0.182** | **0.146** | **0.094** | **0.128** | **0.104** | **0.118** | **0.129** | **0.105** | **0.163** | **0.146** | **0.153** | **0.080** | **0.169** |
| YS4 | 473 | 472 | 337 | 398 | 616 | 673 | 615 | 697 | 701 | 800 | 695 | 687 | 283 | 439 | 455 | 445 | 563 | 586 | 483 | 668 | 579 | 133 | 115 | 22 | 25 |  | **0.023** | **0.040** | **0.085** | **0.187** | **0.147** | **0.069** | **0.111** | **0.102** | **0.097** | **0.105** | **0.080** | **0.161** | **0.141** | **0.145** | **0.080** | **0.172** |
| LWQ1a | 460 | 459 | 323 | 387 | 604 | 661 | 602 | 684 | 688 | 786 | 681 | 673 | 268 | 424 | 440 | 431 | 548 | 571 | 469 | 654 | 565 | 120 | 101 | 14 | 11 | 16 |  | 0.006 | **0.086** | **0.159** | **0.126** | **0.036** | **0.098** | **0.090** | **0.081** | **0.089** | **0.086** | **0.139** | **0.112** | **0.124** | **0.055** | **0.145** |
| LWQ2a | 463 | 461 | 326 | 390 | 606 | 663 | 605 | 686 | 690 | 788 | 683 | 675 | 269 | 426 | 442 | 433 | 550 | 574 | 471 | 656 | 568 | 123 | 104 | 18 | 14 | 16 | 4 |  | **0.080** | **0.177** | **0.142** | 0.003 | **0.083** | **0.095** | **0.079** | **0.067** | **0.063** | **0.145** | **0.122** | **0.129** | **0.060** | **0.164** |
| DQ | 575 | 574 | 438 | 497 | 717 | 774 | 716 | 799 | 803 | 901 | 796 | 788 | 382 | 539 | 555 | 547 | 664 | 687 | 584 | 769 | 681 | 234 | 216 | 124 | 126 | 102 | 116 | 113 |  | **0.181** | **0.147** | **0.068** | **0.093** | **0.089** | **0.030** | **0.044** | **0.033** | **0.157** | **0.131** | **0.137** | **0.050** | **0.159** |
| BQ | 673 | 673 | 537 | 593 | 814 | 872 | 814 | 897 | 901 | 1000 | 895 | 887 | 480 | 638 | 654 | 646 | 762 | 786 | 683 | 868 | 779 | 333 | 315 | 222 | 225 | 200 | 214 | 212 | 99 |  | **0.101** | **0.177** | **0.158** | **0.171** | **0.192** | **0.200** | **0.180** | **0.142** | **0.127** | **0.125** | **0.175** | **0.171** |
| CD | 342 | 340 | 206 | 277 | 488 | 544 | 486 | 565 | 569 | 667 | 562 | 553 | 152 | 305 | 321 | 312 | 428 | 452 | 349 | 535 | 446 | 28 | 26 | 113 | 109 | 134 | 120 | 122 | 235 | 334 |  | **0.135** | **0.119** | **0.129** | **0.145** | **0.160** | **0.148** | **0.034** | **0.033** | **0.038** | **0.129** | **0.069** |
| JD | 237 | 233 | 101 | 187 | 385 | 440 | 381 | 458 | 462 | 560 | 454 | 446 | 63 | 200 | 215 | 204 | 321 | 345 | 242 | 427 | 339 | 112 | 128 | 220 | 217 | 241 | 227 | 229 | 343 | 441 | 107 |  | **0.073** | **0.097** | **0.068** | **0.048** | **0.050** | **0.148** | **0.117** | **0.131** | **0.062** | **0.163** |
| DG | 156 | 150 | 36 | 134 | 307 | 360 | 301 | 376 | 379 | 476 | 371 | 362 | 73 | 121 | 135 | 121 | 238 | 262 | 158 | 344 | 256 | 194 | 211 | 303 | 300 | 325 | 310 | 313 | 426 | 525 | 191 | 84 |  | **0.052** | **0.071** | **0.082** | **0.074** | **0.122** | **0.106** | **0.110** | **0.076** | **0.149** |
| ZD | 554 | 554 | 418 | 476 | 695 | 753 | 695 | 778 | 783 | 881 | 776 | 769 | 365 | 521 | 537 | 527 | 645 | 668 | 565 | 750 | 661 | 214 | 196 | 104 | 107 | 82 | 97 | 95 | 23 | 119 | 216 | 323 | 407 |  | **0.079** | **0.094** | **0.092** | **0.128** | **0.110** | **0.115** | **0.049** | **0.151** |
| MZGKa | 925 | 925 | 789 | 843 | 1064 | 1122 | 1065 | 1149 | 1153 | 1252 | 1147 | 1139 | 730 | 889 | 905 | 898 | 1014 | 1037 | 935 | 1120 | 1031 | 585 | 567 | 474 | 477 | 452 | 466 | 464 | 351 | 252 | 586 | 693 | 777 | 371 |  | **0.040** | **0.054** | **0.153** | **0.121** | **0.128** | **0.033** | **0.160** |
| GBJDa | 900 | 900 | 764 | 818 | 1040 | 1098 | 1040 | 1124 | 1129 | 1227 | 1122 | 1115 | 706 | 864 | 880 | 873 | 989 | 1013 | 910 | 1096 | 1007 | 560 | 542 | 450 | 452 | 428 | 442 | 439 | 326 | 227 | 561 | 669 | 752 | 346 | 25 |  | **0.036** | **0.166** | **0.139** | **0.139** | **0.040** | **0.176** |
| WY | 363 | 365 | 500 | 453 | 231 | 174 | 225 | 140 | 136 | 54 | 147 | 163 | 574 | 419 | 403 | 398 | 292 | 273 | 362 | 178 | 259 | 703 | 722 | 814 | 812 | 836 | 823 | 826 | 938 | 1036 | 705 | 598 | 515 | 917 | 1288 | 1263 |  | **0.156** | **0.127** | **0.125** | **0.061** | **0.152** |
| JZG | 346 | 345 | 481 | 444 | 230 | 178 | 219 | 126 | 119 | 20 | 123 | 131 | 547 | 390 | 373 | 373 | 261 | 240 | 336 | 150 | 238 | 685 | 702 | 795 | 793 | 817 | 803 | 806 | 919 | 1017 | 684 | 577 | 493 | 899 | 1269 | 1245 | 54 |  | 0.020 | **0.030** | **0.141** | **0.042** |
| CZS | 343 | 340 | 477 | 443 | 233 | 182 | 220 | 127 | 119 | 27 | 120 | 126 | 541 | 383 | 366 | 368 | 254 | 233 | 330 | 144 | 234 | 680 | 698 | 790 | 788 | 812 | 798 | 801 | 914 | 1013 | 679 | 571 | 488 | 894 | 1265 | 1240 | 70 | 15 |  | -0.002 | **0.112** | **0.046** |
| XJ | 214 | 207 | 342 | 321 | 148 | 130 | 126 | 74 | 67 | 133 | 47 | 22 | 402 | 244 | 227 | 230 | 115 | 94 | 193 | 25 | 105 | 544 | 562 | 654 | 651 | 676 | 662 | 664 | 777 | 876 | 542 | 435 | 351 | 758 | 1128 | 1103 | 182 | 146 | 139 |  | **0.118** | **0.069** |
| RT | 88 | 78 | 214 | 199 | 125 | 159 | 106 | 155 | 157 | 252 | 147 | 138 | 281 | 129 | 113 | 105 | 40 | 56 | 69 | 119 | 36 | 417 | 434 | 527 | 524 | 549 | 535 | 537 | 650 | 749 | 415 | 308 | 225 | 631 | 1001 | 976 | 293 | 269 | 264 | 128 |  | **0.140** |
| BM | 43 | 24 | 158 | 152 | 154 | 200 | 141 | 207 | 210 | 307 | 202 | 194 | 229 | 88 | 75 | 53 | 80 | 103 | 25 | 175 | 87 | 361 | 379 | 471 | 469 | 493 | 479 | 482 | 595 | 694 | 360 | 253 | 170 | 575 | 946 | 921 | 346 | 324 | 319 | 184 | 56 |  |
