# Supplementary material for: Gene Flow Results in High Genetic Similarity between Sibiraea (Rosaceae) Species in the Qinghai-Tibetan Plateau
Source: Front Plant Sci. 2016 Oct 25;7:1596. doi: 10.3389/fpls.2016.01596 (PMC5078775; doi:10.3389/fpls.2016.01596)
Supplement: Supplementary file 3 [file Table3.DOCX]

Table S3. The individual's estimated proportion of membership in each cluster.

(a) K=2

| P. | Individual | A | B |
| --- | --- | --- | --- |
| P1 | MQb-1 | 0.8765 | 0.1235 |
|  | MQb-2 | 0.9741 | 0.0259 |
|  | MQb-3 | 0.9905 | 0.0095 |
|  | MQb-4 | 0.9934 | 0.0066 |
|  | MQb-5 | 0.9924 | 0.0076 |
|  | MQb-6 | 0.9923 | 0.0077 |
| P2 | DRb-1 | 0.9918 | 0.0082 |
|  | DRb-2 | 0.5602 | 0.4398 |
|  | DRb-3 | 0.8791 | 0.1209 |
|  | DRb-4 | 0.9933 | 0.0067 |
|  | DRb-5 | 0.9932 | 0.0068 |
|  | DRb-6 | 0.9924 | 0.0076 |
|  | DRb-7 | 0.984 | 0.016 |
|  | DRb-8 | 0.9805 | 0.0195 |
|  | DRb-9 | 0.9897 | 0.0103 |
|  | DRb-10 | 0.8127 | 0.1873 |
|  | DRb-11 | 0.9879 | 0.0121 |
|  | DRb-12 | 0.6795 | 0.3205 |
|  | DRb-13 | 0.558 | 0.442 |
|  | DRb-14 | 0.8708 | 0.1292 |
| P3 | QLb-1 | 0.9896 | 0.0104 |
|  | QLb-2 | 0.9943 | 0.0057 |
|  | QLb-3 | 0.9913 | 0.0087 |
|  | QLb-4 | 0.9923 | 0.0077 |
|  | QLb-5 | 0.9921 | 0.0079 |
|  | QLb-6 | 0.9886 | 0.0114 |
|  | QLb-7 | 0.9895 | 0.0105 |
|  | QLb-8 | 0.9906 | 0.0094 |
|  | QLb-9 | 0.9884 | 0.0116 |
|  | QLb-10 | 0.993 | 0.007 |
|  | QLb-11 | 0.994 | 0.006 |
|  | QLb-12 | 0.9943 | 0.0057 |
|  | QLb-13 | 0.9934 | 0.0066 |
|  | QLb-14 | 0.9914 | 0.0086 |
|  | QLb-15 | 0.9943 | 0.0057 |
|  | QLb-16 | 0.9943 | 0.0057 |
|  | QLb-17 | 0.9932 | 0.0068 |
|  | QLb-18 | 0.9933 | 0.0067 |
|  | QLb-19 | 0.9908 | 0.0092 |
| P4 | MYb-1 | 0.9896 | 0.0104 |
|  | MYb-2 | 0.9932 | 0.0068 |
|  | MYb-3 | 0.9904 | 0.0096 |
|  | MYb-4 | 0.9924 | 0.0076 |
|  | MYb-5 | 0.9924 | 0.0076 |
|  | MYb-6 | 0.9925 | 0.0075 |
|  | MYb-7 | 0.9934 | 0.0066 |
|  | MYb-8 | 0.9934 | 0.0066 |
|  | MYb-9 | 0.9942 | 0.0058 |
|  | MYb-10 | 0.9942 | 0.0058 |
|  | MYb-11 | 0.9933 | 0.0067 |
|  | MYb-12 | 0.9933 | 0.0067 |
|  | MYb-13 | 0.9913 | 0.0087 |
|  | MYb-14 | 0.9942 | 0.0058 |
|  | MYb-15 | 0.9895 | 0.0105 |
|  | MYb-16 | 0.9925 | 0.0075 |
|  | MYb-17 | 0.9905 | 0.0095 |
| P5 | HZb-1 | 0.9906 | 0.0094 |
|  | HZb-2 | 0.9924 | 0.0076 |
|  | HZb-3 | 0.9924 | 0.0076 |
|  | HZb-4 | 0.9898 | 0.0102 |
| P6 | PAb-1 | 0.9942 | 0.0058 |
|  | PAb-2 | 0.9867 | 0.0133 |
|  | PAb-3 | 0.993 | 0.007 |
|  | PAb-4 | 0.9856 | 0.0144 |
|  | PAb-5 | 0.9933 | 0.0067 |
|  | PAb-6 | 0.9734 | 0.0266 |
|  | PAb-7 | 0.9924 | 0.0076 |
|  | PAb-8 | 0.9933 | 0.0067 |
|  | PAb-9 | 0.9932 | 0.0068 |
|  | PAb-10 | 0.9933 | 0.0067 |
|  | PAb-11 | 0.9272 | 0.0728 |
|  | PAb-12 | 0.9935 | 0.0065 |
|  | PAb-13 | 0.9884 | 0.0116 |
|  | PAb-14 | 0.9933 | 0.0067 |
|  | PAb-15 | 0.9933 | 0.0067 |
|  | PAb-16 | 0.9856 | 0.0144 |
| P7 | XHb-1 | 0.9874 | 0.0126 |
|  | XHb-2 | 0.9893 | 0.0107 |
|  | XHb-3 | 0.9933 | 0.0067 |
|  | XHb-4 | 0.9893 | 0.0107 |
|  | XHb-5 | 0.9903 | 0.0097 |
|  | XHb-6 | 0.9905 | 0.0095 |
|  | XHb-7 | 0.9913 | 0.0087 |
|  | XHb-8 | 0.9942 | 0.0058 |
|  | XHb-9 | 0.9907 | 0.0093 |
|  | XHb-10 | 0.9625 | 0.0375 |
|  | XHb-11 | 0.9803 | 0.0197 |
|  | XHb-12 | 0.9942 | 0.0058 |
|  | XHb-13 | 0.9881 | 0.0119 |
|  | XHb-14 | 0.9943 | 0.0057 |
|  | XHb-15 | 0.9924 | 0.0076 |
|  | XHb-16 | 0.9546 | 0.0454 |
|  | XHb-17 | 0.9875 | 0.0125 |
| P8 | REG1b-1 | 0.9916 | 0.0084 |
|  | REG1b-2 | 0.99 | 0.01 |
|  | REG1b-3 | 0.7478 | 0.2522 |
|  | REG1b-4 | 0.9922 | 0.0078 |
|  | REG1b-5 | 0.9551 | 0.0449 |
|  | REG1b-6 | 0.9867 | 0.0133 |
|  | REG1b-7 | 0.9924 | 0.0076 |
|  | REG1b-8 | 0.9886 | 0.0114 |
|  | REG1b-9 | 0.9929 | 0.0071 |
|  | REG1b-10 | 0.9882 | 0.0118 |
|  | REG1b-11 | 0.9905 | 0.0095 |
|  | REG1b-12 | 0.9599 | 0.0401 |
| P9 | REG2b-1 | 0.9838 | 0.0162 |
|  | REG2b-2 | 0.7341 | 0.2659 |
|  | REG2b-3 | 0.8118 | 0.1882 |
|  | REG2b-4 | 0.9829 | 0.0171 |
| P10 | REG3b-1 | 0.9912 | 0.0088 |
|  | REG3b-2 | 0.9689 | 0.0311 |
|  | REG3b-3 | 0.9905 | 0.0095 |
|  | REG3b-4 | 0.9858 | 0.0142 |
|  | REG3b-5 | 0.7685 | 0.2315 |
|  | REG3b-6 | 0.9641 | 0.0359 |
|  | REG3b-7 | 0.6765 | 0.3235 |
|  | REG3b-8 | 0.5312 | 0.4688 |
| P11 | BMb-1 | 0.7637 | 0.2363 |
|  | BMb-2 | 0.7145 | 0.2855 |
|  | BMb-3 | 0.9493 | 0.0507 |
|  | BMb-4 | 0.9415 | 0.0585 |
| P12 | YSb-1 | 0.4774 | 0.5226 |
|  | YSb-2 | 0.1362 | 0.8638 |
|  | YSb-3 | 0.6829 | 0.3171 |
|  | YSb-4 | 0.7876 | 0.2124 |
|  | YSb-5 | 0.7047 | 0.2953 |
|  | YSb-6 | 0.5203 | 0.4797 |
|  | YSb-7 | 0.1619 | 0.8381 |
|  | YSb-8 | 0.4078 | 0.5922 |
|  | YSb-9 | 0.0917 | 0.9083 |
| S1 | MQa-1 | 0.7384 | 0.2616 |
|  | MQa-2 | 0.8793 | 0.1207 |
|  | MQa-3 | 0.8761 | 0.1239 |
|  | MQa-4 | 0.8935 | 0.1065 |
|  | MQa-5 | 0.5183 | 0.4817 |
|  | MQa-6 | 0.887 | 0.113 |
|  | MQa-7 | 0.8846 | 0.1154 |
|  | MQa-8 | 0.6756 | 0.3244 |
|  | MQa-9 | 0.7706 | 0.2294 |
|  | MQa-10 | 0.8908 | 0.1092 |
|  | MQa-11 | 0.8963 | 0.1037 |
|  | MQa-12 | 0.816 | 0.184 |
|  | MQa-13 | 0.695 | 0.305 |
|  | MQa-14 | 0.5894 | 0.4106 |
|  | MQa-15 | 0.8068 | 0.1932 |
| S2 | DR1a-1 | 0.0085 | 0.9915 |
|  | DR1a-2 | 0.2128 | 0.7872 |
|  | DR1a-3 | 0.0104 | 0.9896 |
|  | DR1a-4 | 0.0596 | 0.9404 |
|  | DR1a-5 | 0.0086 | 0.9914 |
|  | DR1a-6 | 0.0159 | 0.9841 |
|  | DR1a-7 | 0.0211 | 0.9789 |
|  | DR1a-8 | 0.0076 | 0.9924 |
|  | DR1a-9 | 0.0148 | 0.9852 |
| S3 | DR2a-1 | 0.5502 | 0.4498 |
|  | DR2a-2 | 0.7011 | 0.2989 |
|  | DR2a-3 | 0.5971 | 0.4029 |
|  | DR2a-4 | 0.3965 | 0.6035 |
|  | DR2a-5 | 0.1389 | 0.8611 |
|  | DR2a-6 | 0.8895 | 0.1105 |
|  | DR2a-7 | 0.8306 | 0.1694 |
|  | DR2a-8 | 0.4487 | 0.5513 |
|  | DR2a-9 | 0.9095 | 0.0905 |
|  | DR2a-10 | 0.3223 | 0.6777 |
|  | DR2a-11 | 0.7616 | 0.2384 |
|  | DR2a-12 | 0.8561 | 0.1439 |
| S4 | QLa-1 | 0.9256 | 0.0744 |
|  | QLa-2 | 0.8895 | 0.1105 |
|  | QLa-3 | 0.8925 | 0.1075 |
|  | QLa-4 | 0.8963 | 0.1037 |
|  | QLa-5 | 0.8904 | 0.1096 |
|  | QLa-6 | 0.8922 | 0.1078 |
|  | QLa-7 | 0.8997 | 0.1003 |
|  | QLa-8 | 0.8866 | 0.1134 |
|  | QLa-9 | 0.8938 | 0.1062 |
|  | QLa-10 | 0.8719 | 0.1281 |
|  | QLa-11 | 0.8965 | 0.1035 |
|  | QLa-12 | 0.8768 | 0.1232 |
|  | QLa-13 | 0.4824 | 0.5176 |
|  | QLa-14 | 0.8661 | 0.1339 |
|  | QLa-15 | 0.8916 | 0.1084 |
| S5 | MYa-1 | 0.8992 | 0.1008 |
|  | MYa-2 | 0.8857 | 0.1143 |
|  | MYa-3 | 0.8938 | 0.1062 |
|  | MYa-4 | 0.8906 | 0.1094 |
|  | MYa-5 | 0.8153 | 0.1847 |
|  | MYa-6 | 0.8968 | 0.1032 |
|  | MYa-7 | 0.8766 | 0.1234 |
|  | MYa-8 | 0.8734 | 0.1266 |
|  | MYa-9 | 0.89 | 0.11 |
|  | MYa-10 | 0.8757 | 0.1243 |
|  | MYa-11 | 0.8839 | 0.1161 |
|  | MYa-12 | 0.8926 | 0.1074 |
|  | MYa-13 | 0.8904 | 0.1096 |
|  | MYa-14 | 0.8857 | 0.1143 |
|  | MYa-15 | 0.832 | 0.168 |
|  | MYa-16 | 0.8884 | 0.1116 |
|  | MYa-17 | 0.8422 | 0.1578 |
|  | MYa-18 | 0.8461 | 0.1539 |
|  | MYa-19 | 0.8745 | 0.1255 |
|  | MYa-20 | 0.893 | 0.107 |
|  | MYa-21 | 0.893 | 0.107 |
|  | MYa-22 | 0.7505 | 0.2495 |
|  | MYa-23 | 0.8917 | 0.1083 |
|  | MYa-24 | 0.8793 | 0.1207 |
|  | MYa-25 | 0.8607 | 0.1393 |
| S6 | HZa-1 | 0.8799 | 0.1201 |
|  | HZa-2 | 0.8868 | 0.1132 |
|  | HZa-3 | 0.8896 | 0.1104 |
|  | HZa-4 | 0.8913 | 0.1087 |
|  | HZa-5 | 0.8974 | 0.1026 |
|  | HZa-6 | 0.8804 | 0.1196 |
|  | HZa-7 | 0.6968 | 0.3032 |
| S7 | PAa-1 | 0.8256 | 0.1744 |
|  | PAa-2 | 0.6905 | 0.3095 |
|  | PAa-3 | 0.7229 | 0.2771 |
|  | PAa-4 | 0.8732 | 0.1268 |
|  | PAa-5 | 0.8962 | 0.1038 |
|  | PAa-6 | 0.8786 | 0.1214 |
|  | PAa-7 | 0.4773 | 0.5227 |
|  | PAa-8 | 0.8848 | 0.1152 |
|  | PAa-9 | 0.8897 | 0.1103 |
|  | PAa-10 | 0.8831 | 0.1169 |
|  | PAa-11 | 0.8812 | 0.1188 |
|  | PAa-12 | 0.8936 | 0.1064 |
|  | PAa-13 | 0.8676 | 0.1324 |
|  | PAa-14 | 0.8184 | 0.1816 |
|  | PAa-15 | 0.8935 | 0.1065 |
|  | PAa-16 | 0.877 | 0.123 |
|  | PAa-17 | 0.8572 | 0.1428 |
|  | PAa-18 | 0.8851 | 0.1149 |
|  | PAa-19 | 0.8274 | 0.1726 |
|  | PAa-20 | 0.8979 | 0.1021 |
|  | PAa-21 | 0.9166 | 0.0834 |
| S8 | XHa-1 | 0.8717 | 0.1283 |
|  | XHa-2 | 0.9327 | 0.0673 |
|  | XHa-3 | 0.8638 | 0.1362 |
|  | XHa-4 | 0.745 | 0.255 |
|  | XHa-5 | 0.486 | 0.514 |
|  | XHa-6 | 0.8894 | 0.1106 |
|  | XHa-7 | 0.084 | 0.916 |
|  | XHa-8 | 0.8881 | 0.1119 |
|  | XHa-9 | 0.889 | 0.111 |
|  | XHa-10 | 0.8719 | 0.1281 |
|  | XHa-11 | 0.2751 | 0.7249 |
|  | XHa-12 | 0.3101 | 0.6899 |
|  | XHa-13 | 0.8059 | 0.1941 |
|  | XHa-14 | 0.8863 | 0.1137 |
|  | XHa-15 | 0.8264 | 0.1736 |
|  | XHa-16 | 0.8782 | 0.1218 |
|  | XHa-17 | 0.8386 | 0.1614 |
|  | XHa-18 | 0.9013 | 0.0987 |
| S9 | REG1a-1 | 0.1328 | 0.8672 |
|  | REG1a-2 | 0.0245 | 0.9755 |
|  | REG1a-3 | 0.8165 | 0.1835 |
|  | REG1a-4 | 0.3599 | 0.6401 |
|  | REG1a-5 | 0.0955 | 0.9045 |
|  | REG1a-6 | 0.6012 | 0.3988 |
|  | REG1a-7 | 0.8824 | 0.1176 |
|  | REG1a-8 | 0.8879 | 0.1121 |
|  | REG1a-9 | 0.8731 | 0.1269 |
| S10 | REG2a-2 | 0.0733 | 0.9267 |
|  | REG2a-3 | 0.7807 | 0.2193 |
|  | REG2a-4 | 0.0753 | 0.9247 |
|  | REG2a-5 | 0.4541 | 0.5459 |
|  | REG2a-6 | 0.7804 | 0.2196 |
|  | REG2a-7 | 0.7442 | 0.2558 |
|  | REG2a-8 | 0.3256 | 0.6744 |
|  | REG2a-9 | 0.158 | 0.842 |
|  | REG2a-10 | 0.0569 | 0.9431 |
|  | REG2a-11 | 0.183 | 0.817 |
|  | REG2a-12 | 0.4391 | 0.5609 |
| S11 | REG3a-3 | 0.1705 | 0.8295 |
|  | REG3a-4 | 0.2889 | 0.7111 |
|  | REG3a-5 | 0.0661 | 0.9339 |
|  | REG3a-6 | 0.1423 | 0.8577 |
|  | REG3a-7 | 0.1495 | 0.8505 |
|  | REG3a-8 | 0.115 | 0.885 |
|  | REG3a-9 | 0.0166 | 0.9834 |
|  | REG3a-10 | 0.0462 | 0.9538 |
|  | REG3a-11 | 0.147 | 0.853 |
|  | REG3a-12 | 0.0331 | 0.9669 |
|  | REG3a-13 | 0.1092 | 0.8908 |
|  | REG3a-14 | 0.013 | 0.987 |
|  | REG3a-15 | 0.0264 | 0.9736 |
|  | REG3a-16 | 0.0458 | 0.9542 |
|  | REG3a-17 | 0.0238 | 0.9762 |
| S12 | HYa-1 | 0.0265 | 0.9735 |
|  | HYa-2 | 0.0084 | 0.9916 |
|  | HYa-3 | 0.0573 | 0.9427 |
|  | HYa-4 | 0.0151 | 0.9849 |
|  | HYa-5 | 0.0528 | 0.9472 |
|  | HYa-6 | 0.0191 | 0.9809 |
|  | HYa-7 | 0.0124 | 0.9876 |
|  | HYa-8 | 0.0212 | 0.9788 |
|  | HYa-9 | 0.0085 | 0.9915 |
|  | HYa-10 | 0.015 | 0.985 |
|  | HYa-11 | 0.0296 | 0.9704 |
|  | HYa-12 | 0.0148 | 0.9852 |
|  | HYa-13 | 0.0109 | 0.9891 |
|  | HYa-14 | 0.15 | 0.85 |
| S13 | DQINa-1 | 0.0709 | 0.9291 |
|  | DQINa-2 | 0.0882 | 0.9118 |
|  | DQINa-3 | 0.0177 | 0.9823 |
|  | DQINa-4 | 0.0328 | 0.9672 |
|  | DQINa-5 | 0.0275 | 0.9725 |
|  | DQINa-6 | 0.0502 | 0.9498 |
|  | DQINa-7 | 0.0835 | 0.9165 |
|  | DQINa-8 | 0.0157 | 0.9843 |
|  | DQINa-9 | 0.0288 | 0.9712 |
|  | DQINa-10 | 0.1788 | 0.8212 |
|  | DQINa-11 | 0.2624 | 0.7376 |
|  | DQINa-12 | 0.074 | 0.926 |
|  | DQINa-13 | 0.0293 | 0.9707 |
|  | DQINa-14 | 0.0265 | 0.9735 |
|  | DQINa-15 | 0.1093 | 0.8907 |
|  | DQINa-16 | 0.044 | 0.956 |
|  | DQINa-17 | 0.0235 | 0.9765 |
|  | DQINa-18 | 0.0157 | 0.9843 |
|  | DQINa-19 | 0.1793 | 0.8207 |
|  | DQINa-20 | 0.0325 | 0.9675 |
|  | DQINa-21 | 0.049 | 0.951 |
|  | DQINa-22 | 0.0175 | 0.9825 |
|  | DQINa-23 | 0.016 | 0.984 |
|  | DQINa-24 | 0.033 | 0.967 |
|  | DQINa-25 | 0.0318 | 0.9682 |
| S14 | DCa-1 | 0.0539 | 0.9461 |
|  | DCa-2 | 0.0872 | 0.9128 |
|  | DCa-3 | 0.0145 | 0.9855 |
|  | DCa-4 | 0.0619 | 0.9381 |
|  | DCa-5 | 0.0094 | 0.9906 |
|  | DCa-6 | 0.027 | 0.973 |
|  | DCa-7 | 0.0093 | 0.9907 |
|  | DCa-8 | 0.0586 | 0.9414 |
|  | DCa-9 | 0.0813 | 0.9187 |
|  | DCa-10 | 0.0185 | 0.9815 |
|  | DCa-11 | 0.0788 | 0.9212 |
|  | DCa-12 | 0.0263 | 0.9737 |
|  | DCa-13 | 0.0529 | 0.9471 |
|  | DCa-14 | 0.0106 | 0.9894 |
|  | DCa-15 | 0.0887 | 0.9113 |
|  | DCa-16 | 0.0597 | 0.9403 |
|  | DCa-17 | 0.0104 | 0.9896 |
|  | DCa-18 | 0.0484 | 0.9516 |
|  | DCa-19 | 0.0262 | 0.9738 |
|  | DCa-20 | 0.4774 | 0.5226 |
|  | DCa-21 | 0.0225 | 0.9775 |
|  | DCa-22 | 0.0257 | 0.9743 |
|  | DCa-23 | 0.1672 | 0.8328 |
|  | DCa-24 | 0.0102 | 0.9898 |
|  | DCa-25 | 0.0221 | 0.9779 |
|  | DCa-26 | 0.0112 | 0.9888 |
|  | DCa-27 | 0.0118 | 0.9882 |
|  | DCa-28 | 0.0123 | 0.9877 |
|  | DCa-29 | 0.0054 | 0.9946 |
| S15 | LTa-1 | 0.0157 | 0.9843 |
|  | LTa-2 | 0.0144 | 0.9856 |
|  | LTa-3 | 0.014 | 0.986 |
|  | LTa-4 | 0.0067 | 0.9933 |
|  | LTa-5 | 0.0163 | 0.9837 |
|  | LTa-6 | 0.0118 | 0.9882 |
|  | LTa-7 | 0.0316 | 0.9684 |
|  | LTa-8 | 0.0259 | 0.9741 |
|  | LTa-9 | 0.0126 | 0.9874 |
|  | LTa-10 | 0.0164 | 0.9836 |
|  | LTa-11 | 0.012 | 0.988 |
|  | LTa-12 | 0.0257 | 0.9743 |
|  | LTa-13 | 0.0554 | 0.9446 |
|  | LTa-14 | 0.0456 | 0.9544 |
|  | LTa-15 | 0.0135 | 0.9865 |
|  | LTa-16 | 0.0169 | 0.9831 |
|  | LTa-17 | 0.0468 | 0.9532 |
|  | LTa-18 | 0.0156 | 0.9844 |
|  | LTa-19 | 0.0149 | 0.9851 |
|  | LTa-20 | 0.0266 | 0.9734 |
|  | LTa-21 | 0.1854 | 0.8146 |
|  | LTa-22 | 0.0115 | 0.9885 |
| S16 | GZa-1 | 0.0105 | 0.9895 |
|  | GZa-2 | 0.0094 | 0.9906 |
|  | GZa-3 | 0.0084 | 0.9916 |
|  | GZa-4 | 0.0186 | 0.9814 |
|  | GZa-5 | 0.0223 | 0.9777 |
|  | GZa-6 | 0.0265 | 0.9735 |
|  | GZa-7 | 0.2963 | 0.7037 |
|  | GZa-8 | 0.0376 | 0.9624 |
|  | GZa-9 | 0.0579 | 0.9421 |
|  | GZa-10 | 0.0809 | 0.9191 |
|  | GZa-11 | 0.0273 | 0.9727 |
|  | GZa-12 | 0.0179 | 0.9821 |
|  | GZa-13 | 0.1176 | 0.8824 |
|  | GZa-14 | 0.0969 | 0.9031 |
|  | GZa-15 | 0.0121 | 0.9879 |
|  | GZa-16 | 0.0076 | 0.9924 |
|  | GZa-17 | 0.0115 | 0.9885 |
|  | GZa-18 | 0.0541 | 0.9459 |
|  | GZa-19 | 0.0506 | 0.9494 |
|  | GZa-20 | 0.0332 | 0.9668 |
|  | GZa-21 | 0.0148 | 0.9852 |
|  | GZa-22 | 0.0262 | 0.9738 |
|  | GZa-23 | 0.0919 | 0.9081 |
|  | GZa-24 | 0.1385 | 0.8615 |
| S17 | DFa-1 | 0.0199 | 0.9801 |
|  | DFa-2 | 0.0561 | 0.9439 |
|  | DFa-3 | 0.0134 | 0.9866 |
|  | DFa-4 | 0.0087 | 0.9913 |
|  | DFa-5 | 0.0301 | 0.9699 |
|  | DFa-6 | 0.0207 | 0.9793 |
|  | DFa-7 | 0.0077 | 0.9923 |
|  | DFa-8 | 0.0149 | 0.9851 |
|  | DFa-9 | 0.0113 | 0.9887 |
|  | DFa-10 | 0.0123 | 0.9877 |
|  | DFa-11 | 0.1335 | 0.8665 |
|  | DFa-12 | 0.1428 | 0.8572 |
|  | DFa-13 | 0.0112 | 0.9888 |
|  | DFa-14 | 0.0327 | 0.9673 |
|  | DFa-15 | 0.0451 | 0.9549 |
|  | DFa-16 | 0.0252 | 0.9748 |
|  | DFa-17 | 0.0113 | 0.9887 |
|  | DFa-18 | 0.0093 | 0.9907 |
|  | DFa-19 | 0.0135 | 0.9865 |
| S18 | DBa-1 | 0.0094 | 0.9906 |
|  | DBa-2 | 0.0147 | 0.9853 |
|  | DBa-3 | 0.0274 | 0.9726 |
|  | DBa-4 | 0.0528 | 0.9472 |
|  | DBa-5 | 0.0142 | 0.9858 |
|  | DBa-6 | 0.0075 | 0.9925 |
|  | DBa-7 | 0.0255 | 0.9745 |
|  | DBa-8 | 0.0804 | 0.9196 |
|  | DBa-9 | 0.0149 | 0.9851 |
|  | DBa-10 | 0.0171 | 0.9829 |
|  | DBa-11 | 0.4886 | 0.5114 |
|  | DBa-12 | 0.0198 | 0.9802 |
|  | DBa-13 | 0.014 | 0.986 |
|  | DBa-14 | 0.0113 | 0.9887 |
|  | DBa-15 | 0.028 | 0.972 |
|  | DBa-16 | 0.0603 | 0.9397 |
| S19 | LHa-1 | 0.0097 | 0.9903 |
|  | LHa-2 | 0.0154 | 0.9846 |
|  | LHa-3 | 0.0133 | 0.9867 |
|  | LHa-4 | 0.0057 | 0.9943 |
|  | LHa-5 | 0.0066 | 0.9934 |
|  | LHa-6 | 0.0072 | 0.9928 |
|  | LHa-7 | 0.0204 | 0.9796 |
|  | LHa-8 | 0.0187 | 0.9813 |
|  | LHa-9 | 0.0076 | 0.9924 |
|  | LHa-10 | 0.0102 | 0.9898 |
|  | LHa-11 | 0.0095 | 0.9905 |
|  | LHa-12 | 0.0123 | 0.9877 |
|  | LHa-13 | 0.0122 | 0.9878 |
|  | LHa-14 | 0.0066 | 0.9934 |
| S20 | ABa-1 | 0.0115 | 0.9885 |
|  | ABa-2 | 0.0254 | 0.9746 |
|  | ABa-3 | 0.0127 | 0.9873 |
|  | ABa-4 | 0.009 | 0.991 |
|  | ABa-5 | 0.0162 | 0.9838 |
|  | ABa-6 | 0.0077 | 0.9923 |
|  | ABa-7 | 0.0423 | 0.9577 |
|  | ABa-8 | 0.0236 | 0.9764 |
|  | ABa-9 | 0.0223 | 0.9777 |
|  | ABa-10 | 0.0273 | 0.9727 |
|  | ABa-11 | 0.0278 | 0.9722 |
|  | ABa-12 | 0.0048 | 0.9952 |
| S21 | JZa-1 | 0.0164 | 0.9836 |
|  | JZa-2 | 0.014 | 0.986 |
|  | JZa-3 | 0.0541 | 0.9459 |
|  | JZa-4 | 0.0911 | 0.9089 |
|  | JZa-5 | 0.1253 | 0.8747 |
|  | JZa-6 | 0.025 | 0.975 |
|  | JZa-7 | 0.014 | 0.986 |
|  | JZa-8 | 0.0639 | 0.9361 |
|  | JZa-9 | 0.0289 | 0.9711 |
|  | JZa-10 | 0.0114 | 0.9886 |
|  | JZa-11 | 0.0168 | 0.9832 |
|  | JZa-12 | 0.0149 | 0.9851 |
|  | JZa-13 | 0.0201 | 0.9799 |
|  | JZa-14 | 0.0261 | 0.9739 |
|  | JZa-15 | 0.0113 | 0.9887 |
|  | JZa-16 | 0.0744 | 0.9256 |
| S22 | YS1a-1 | 0.0216 | 0.9784 |
|  | YS1a-2 | 0.0077 | 0.9923 |
|  | YS1a-3 | 0.043 | 0.957 |
|  | YS1a-4 | 0.0076 | 0.9924 |
|  | YS1a-5 | 0.0112 | 0.9888 |
|  | YS1a-6 | 0.0132 | 0.9868 |
|  | YS1a-7 | 0.0077 | 0.9923 |
|  | YS1a-8 | 0.0271 | 0.9729 |
|  | YS1a-9 | 0.0096 | 0.9904 |
|  | YS1a-10 | 0.7038 | 0.2962 |
|  | YS1a-11 | 0.0225 | 0.9775 |
|  | YS1a-12 | 0.0181 | 0.9819 |
|  | YS1a-13 | 0.0075 | 0.9925 |
|  | YS1a-14 | 0.0057 | 0.9943 |
|  | YS1a-15 | 0.0145 | 0.9855 |
|  | YS1a-16 | 0.0152 | 0.9848 |
|  | YS1a-17 | 0.0175 | 0.9825 |
|  | YS1a-18 | 0.0085 | 0.9915 |
| S23 | YS2a-1 | 0.0066 | 0.9934 |
|  | YS2a-2 | 0.0275 | 0.9725 |
|  | YS2a-3 | 0.0108 | 0.9892 |
|  | YS2a-4 | 0.0406 | 0.9594 |
|  | YS2a-5 | 0.0167 | 0.9833 |
|  | YS2a-6 | 0.0108 | 0.9892 |
|  | YS2a-7 | 0.0085 | 0.9915 |
|  | YS2a-8 | 0.0146 | 0.9854 |
|  | YS2a-9 | 0.0259 | 0.9741 |
|  | YS2a-10 | 0.0123 | 0.9877 |
|  | YS2a-11 | 0.2976 | 0.7024 |
|  | YS2a-12 | 0.0598 | 0.9402 |
|  | YS2a-13 | 0.0247 | 0.9753 |
|  | YS2a-14 | 0.0152 | 0.9848 |
|  | YS2a-15 | 0.0164 | 0.9836 |
|  | YS2a-16 | 0.0173 | 0.9827 |
|  | YS2a-17 | 0.0115 | 0.9885 |
| S24 | YS3a-1 | 0.0067 | 0.9933 |
|  | YS3a-2 | 0.0069 | 0.9931 |
|  | YS3a-3 | 0.0076 | 0.9924 |
|  | YS3a-4 | 0.0076 | 0.9924 |
|  | YS3a-5 | 0.0114 | 0.9886 |
|  | YS3a-6 | 0.0073 | 0.9927 |
|  | YS3a-7 | 0.0121 | 0.9879 |
|  | YS3a-8 | 0.0115 | 0.9885 |
|  | YS3a-9 | 0.0107 | 0.9893 |
|  | YS3a-10 | 0.4241 | 0.5759 |
| S25 | NQa-1 | 0.0118 | 0.9882 |
|  | NQa-2 | 0.0146 | 0.9854 |
|  | NQa-3 | 0.0104 | 0.9896 |
|  | NQa-4 | 0.0159 | 0.9841 |
|  | NQa-5 | 0.0113 | 0.9887 |
|  | NQa-6 | 0.0119 | 0.9881 |
|  | NQa-7 | 0.0101 | 0.9899 |
|  | NQa-8 | 0.0066 | 0.9934 |
|  | NQa-9 | 0.0092 | 0.9908 |
|  | NQa-10 | 0.0273 | 0.9727 |
|  | NQa-11 | 0.0186 | 0.9814 |
|  | NQa-12 | 0.0149 | 0.9851 |
|  | NQa-13 | 0.0131 | 0.9869 |
|  | NQa-14 | 0.0122 | 0.9878 |
|  | NQa-15 | 0.0067 | 0.9933 |
|  | NQa-16 | 0.0106 | 0.9894 |
|  | NQa-17 | 0.0086 | 0.9914 |
|  | NQa-18 | 0.038 | 0.962 |
|  | NQa-19 | 0.0118 | 0.9882 |
| S26 | YS4a-1 | 0.0147 | 0.9853 |
|  | YS4a-2 | 0.1014 | 0.8986 |
|  | YS4a-3 | 0.0095 | 0.9905 |
|  | YS4a-4 | 0.0131 | 0.9869 |
|  | YS4a-5 | 0.0223 | 0.9777 |
|  | YS4a-6 | 0.015 | 0.985 |
|  | YS4a-7 | 0.0057 | 0.9943 |
|  | YS4a-8 | 0.0121 | 0.9879 |
|  | YS4a-9 | 0.0115 | 0.9885 |
|  | YS4a-10 | 0.0143 | 0.9857 |
|  | YS4a-11 | 0.0131 | 0.9869 |
|  | YS4a-12 | 0.0084 | 0.9916 |
| S27 | LWQ1a-1 | 0.0095 | 0.9905 |
|  | LWQ1a-2 | 0.0125 | 0.9875 |
|  | LWQ1a-3 | 0.0087 | 0.9913 |
|  | LWQ1a-4 | 0.0144 | 0.9856 |
|  | LWQ1a-5 | 0.0821 | 0.9179 |
|  | LWQ1a-6 | 0.0108 | 0.9892 |
|  | LWQ1a-7 | 0.0078 | 0.9922 |
|  | LWQ1a-8 | 0.0154 | 0.9846 |
|  | LWQ1a-9 | 0.0067 | 0.9933 |
|  | LWQ1a-10 | 0.0105 | 0.9895 |
|  | LWQ1a-11 | 0.0099 | 0.9901 |
|  | LWQ1a-12 | 0.0086 | 0.9914 |
|  | LWQ1a-13 | 0.0094 | 0.9906 |
|  | LWQ1a-14 | 0.0203 | 0.9797 |
|  | LWQ1a-15 | 0.0074 | 0.9926 |
|  | LWQ1a-16 | 0.0057 | 0.9943 |
| S28 | LWQ2a-1 | 0.0282 | 0.9718 |
|  | LWQ2a-2 | 0.0128 | 0.9872 |
|  | LWQ2a-3 | 0.0132 | 0.9868 |
|  | LWQ2a-4 | 0.0259 | 0.9741 |
|  | LWQ2a-5 | 0.029 | 0.971 |
|  | LWQ2a-6 | 0.0115 | 0.9885 |
|  | LWQ2a-7 | 0.0233 | 0.9767 |
|  | LWQ2a-8 | 0.0103 | 0.9897 |
|  | LWQ2a-9 | 0.0076 | 0.9924 |
|  | LWQ2a-10 | 0.0086 | 0.9914 |
|  | LWQ2a-11 | 0.0107 | 0.9893 |
|  | LWQ2a-12 | 0.0145 | 0.9855 |
|  | LWQ2a-13 | 0.0911 | 0.9089 |
|  | LWQ2a-14 | 0.0104 | 0.9896 |
|  | LWQ2a-15 | 0.0399 | 0.9601 |
|  | LWQ2a-16 | 0.0091 | 0.9909 |
|  | LWQ2a-17 | 0.0094 | 0.9906 |
|  | LWQ2a-18 | 0.019 | 0.981 |
|  | LWQ2a-19 | 0.0201 | 0.9799 |
|  | LWQ2a-20 | 0.0223 | 0.9777 |
| S29 | DQa-1 | 0.013 | 0.987 |
|  | DQa-2 | 0.0156 | 0.9844 |
|  | DQa-3 | 0.0131 | 0.9869 |
|  | DQa-4 | 0.0196 | 0.9804 |
|  | DQa-5 | 0.0131 | 0.9869 |
|  | DQa-6 | 0.0272 | 0.9728 |
|  | DQa-7 | 0.0154 | 0.9846 |
|  | DQa-8 | 0.1184 | 0.8816 |
|  | DQa-9 | 0.0066 | 0.9934 |
|  | DQa-10 | 0.0239 | 0.9761 |
|  | DQa-11 | 0.0075 | 0.9925 |
|  | DQa-12 | 0.0727 | 0.9273 |
|  | DQa-13 | 0.0616 | 0.9384 |
|  | DQa-14 | 0.0194 | 0.9806 |
|  | DQa-15 | 0.0432 | 0.9568 |
| S30 | BQa-1 | 0.0376 | 0.9624 |
|  | BQa-2 | 0.0096 | 0.9904 |
|  | BQa-3 | 0.0259 | 0.9741 |
|  | BQa-4 | 0.013 | 0.987 |
|  | BQa-5 | 0.0201 | 0.9799 |
|  | BQa-6 | 0.0451 | 0.9549 |
|  | BQa-7 | 0.0727 | 0.9273 |
|  | BQa-8 | 0.0096 | 0.9904 |
|  | BQa-9 | 0.0088 | 0.9912 |
|  | BQa-10 | 0.0117 | 0.9883 |
|  | BQa-11 | 0.0287 | 0.9713 |
|  | BQa-12 | 0.0149 | 0.9851 |
|  | BQa-13 | 0.2583 | 0.7417 |
|  | BQa-14 | 0.0088 | 0.9912 |
|  | BQa-15 | 0.0219 | 0.9781 |
|  | BQa-16 | 0.0077 | 0.9923 |
|  | BQa-17 | 0.0128 | 0.9872 |
|  | BQa-18 | 0.0142 | 0.9858 |
|  | BQa-19 | 0.0076 | 0.9924 |
|  | BQa-20 | 0.0088 | 0.9912 |
|  | BQa-21 | 0.0261 | 0.9739 |
|  | BQa-22 | 0.0077 | 0.9923 |
|  | BQa-23 | 0.0571 | 0.9429 |
|  | BQa-24 | 0.0183 | 0.9817 |
| S31 | CDa-1 | 0.0164 | 0.9836 |
|  | CDa-2 | 0.0161 | 0.9839 |
|  | CDa-3 | 0.0195 | 0.9805 |
|  | CDa-4 | 0.0058 | 0.9942 |
|  | CDa-5 | 0.0144 | 0.9856 |
|  | CDa-6 | 0.0066 | 0.9934 |
|  | CDa-7 | 0.0366 | 0.9634 |
|  | CDa-8 | 0.0219 | 0.9781 |
|  | CDa-9 | 0.0067 | 0.9933 |
|  | CDa-10 | 0.0058 | 0.9942 |
|  | CDa-11 | 0.0057 | 0.9943 |
|  | CDa-12 | 0.0103 | 0.9897 |
|  | CDa-13 | 0.0414 | 0.9586 |
|  | CDa-14 | 0.0081 | 0.9919 |
|  | CDa-15 | 0.0115 | 0.9885 |
|  | CDa-16 | 0.0095 | 0.9905 |
|  | CDa-17 | 0.0144 | 0.9856 |
|  | CDa-18 | 0.0106 | 0.9894 |
|  | CDa-19 | 0.0076 | 0.9924 |
|  | CDa-20 | 0.0094 | 0.9906 |
|  | CDa-21 | 0.0534 | 0.9466 |
|  | CDa-22 | 0.0221 | 0.9779 |
|  | CDa-23 | 0.0865 | 0.9135 |
|  | CDa-24 | 0.0135 | 0.9865 |
| S32 | JDa-1 | 0.0447 | 0.9553 |
|  | JDa-2 | 0.0161 | 0.9839 |
|  | JDa-3 | 0.0127 | 0.9873 |
|  | JDa-4 | 0.0068 | 0.9932 |
|  | JDa-5 | 0.0066 | 0.9934 |
|  | JDa-6 | 0.0092 | 0.9908 |
|  | JDa-7 | 0.0101 | 0.9899 |
|  | JDa-8 | 0.0078 | 0.9922 |
|  | JDa-9 | 0.0383 | 0.9617 |
|  | JDa-10 | 0.0067 | 0.9933 |
|  | JDa-11 | 0.0156 | 0.9844 |
|  | JDa-12 | 0.0142 | 0.9858 |
|  | JDa-13 | 0.0097 | 0.9903 |
|  | JDa-14 | 0.0103 | 0.9897 |
| S33 | DGa-1 | 0.0066 | 0.9934 |
|  | DGa-2 | 0.0077 | 0.9923 |
|  | DGa-3 | 0.0077 | 0.9923 |
|  | DGa-4 | 0.0196 | 0.9804 |
|  | DGa-5 | 0.024 | 0.976 |
|  | DGa-6 | 0.0076 | 0.9924 |
|  | DGa-7 | 0.0309 | 0.9691 |
|  | DGa-8 | 0.0119 | 0.9881 |
|  | DGa-9 | 0.0088 | 0.9912 |
|  | DGa-10 | 0.0159 | 0.9841 |
|  | DGa-11 | 0.0093 | 0.9907 |
|  | DGa-12 | 0.1106 | 0.8894 |
|  | DGa-13 | 0.017 | 0.983 |
|  | DGa-14 | 0.0085 | 0.9915 |

(b) K=7

| P. | Individual | A | B | C | D | E | F | G |
| --- | --- | --- | --- | --- | --- | --- | --- | --- |
| P1 | MQb-1 | 0.0296 | 0.7764 | 0.0265 | 0.0191 | 0.0047 | 0.1384 | 0.0053 |
|  | MQb-2 | 0.0066 | 0.7363 | 0.1588 | 0.007 | 0.0044 | 0.0809 | 0.006 |
|  | MQb-3 | 0.0049 | 0.9654 | 0.008 | 0.005 | 0.005 | 0.0051 | 0.0066 |
|  | MQb-4 | 0.004 | 0.9715 | 0.0085 | 0.003 | 0.004 | 0.004 | 0.005 |
|  | MQb-5 | 0.0044 | 0.9712 | 0.0052 | 0.0042 | 0.004 | 0.005 | 0.006 |
|  | MQb-6 | 0.0072 | 0.9628 | 0.0076 | 0.005 | 0.0038 | 0.005 | 0.0086 |
| P2 | DRb-1 | 0.0096 | 0.905 | 0.0168 | 0.0069 | 0.005 | 0.0106 | 0.0461 |
|  | DRb-2 | 0.2579 | 0.4926 | 0.0837 | 0.0106 | 0.0047 | 0.1432 | 0.0073 |
|  | DRb-3 | 0.084 | 0.495 | 0.2862 | 0.014 | 0.019 | 0.0777 | 0.0242 |
|  | DRb-4 | 0.0046 | 0.9645 | 0.0076 | 0.004 | 0.004 | 0.004 | 0.0113 |
|  | DRb-5 | 0.004 | 0.9554 | 0.007 | 0.003 | 0.005 | 0.0086 | 0.017 |
|  | DRb-6 | 0.0047 | 0.8598 | 0.1061 | 0.007 | 0.0049 | 0.006 | 0.0115 |
|  | DRb-7 | 0.0084 | 0.7686 | 0.1612 | 0.005 | 0.0069 | 0.0439 | 0.006 |
|  | DRb-8 | 0.009 | 0.9522 | 0.0074 | 0.0054 | 0.0139 | 0.0061 | 0.006 |
|  | DRb-9 | 0.004 | 0.9689 | 0.0059 | 0.006 | 0.006 | 0.004 | 0.0052 |
|  | DRb-10 | 0.0169 | 0.498 | 0.4416 | 0.0189 | 0.0075 | 0.0112 | 0.006 |
|  | DRb-11 | 0.0052 | 0.9175 | 0.0463 | 0.0068 | 0.012 | 0.0056 | 0.0066 |
|  | DRb-12 | 0.0988 | 0.5752 | 0.0681 | 0.0252 | 0.1444 | 0.0804 | 0.008 |
|  | DRb-13 | 0.0241 | 0.2163 | 0.4569 | 0.0233 | 0.209 | 0.0307 | 0.0397 |
|  | DRb-14 | 0.0168 | 0.7668 | 0.027 | 0.1369 | 0.0234 | 0.0231 | 0.006 |
| P3 | QLb-1 | 0.0147 | 0.9475 | 0.0139 | 0.0058 | 0.0068 | 0.0046 | 0.0067 |
|  | QLb-2 | 0.003 | 0.9758 | 0.0072 | 0.003 | 0.003 | 0.003 | 0.005 |
|  | QLb-3 | 0.0058 | 0.9676 | 0.0058 | 0.003 | 0.0077 | 0.0031 | 0.007 |
|  | QLb-4 | 0.0043 | 0.956 | 0.0088 | 0.0056 | 0.0045 | 0.0108 | 0.01 |
|  | QLb-5 | 0.0046 | 0.93 | 0.0391 | 0.0058 | 0.0063 | 0.0046 | 0.0096 |
|  | QLb-6 | 0.008 | 0.9606 | 0.0076 | 0.004 | 0.008 | 0.004 | 0.0078 |
|  | QLb-7 | 0.0062 | 0.9672 | 0.0044 | 0.004 | 0.0084 | 0.004 | 0.0058 |
|  | QLb-8 | 0.0098 | 0.8912 | 0.076 | 0.004 | 0.0042 | 0.0098 | 0.005 |
|  | QLb-9 | 0.004 | 0.9153 | 0.0269 | 0.006 | 0.0066 | 0.0157 | 0.0255 |
|  | QLb-10 | 0.0035 | 0.9746 | 0.0048 | 0.004 | 0.0031 | 0.004 | 0.006 |
|  | QLb-11 | 0.0037 | 0.9541 | 0.0187 | 0.004 | 0.004 | 0.0037 | 0.0118 |
|  | QLb-12 | 0.0035 | 0.9795 | 0.004 | 0.003 | 0.003 | 0.003 | 0.004 |
|  | QLb-13 | 0.0036 | 0.979 | 0.004 | 0.003 | 0.003 | 0.003 | 0.0044 |
|  | QLb-14 | 0.0036 | 0.9694 | 0.0058 | 0.005 | 0.0059 | 0.004 | 0.0063 |
|  | QLb-15 | 0.0035 | 0.9779 | 0.0046 | 0.003 | 0.003 | 0.003 | 0.005 |
|  | QLb-16 | 0.0036 | 0.9585 | 0.0143 | 0.004 | 0.0042 | 0.0059 | 0.0095 |
|  | QLb-17 | 0.0086 | 0.9632 | 0.0066 | 0.003 | 0.004 | 0.0044 | 0.0102 |
|  | QLb-18 | 0.0036 | 0.9649 | 0.01 | 0.0036 | 0.004 | 0.0046 | 0.0093 |
|  | QLb-19 | 0.004 | 0.9653 | 0.0082 | 0.005 | 0.005 | 0.0074 | 0.0051 |
| P4 | MYb-1 | 0.006 | 0.9602 | 0.0076 | 0.004 | 0.004 | 0.0116 | 0.0066 |
|  | MYb-2 | 0.0038 | 0.974 | 0.0058 | 0.0036 | 0.004 | 0.004 | 0.0048 |
|  | MYb-3 | 0.0063 | 0.9558 | 0.0104 | 0.0062 | 0.0046 | 0.0084 | 0.0083 |
|  | MYb-4 | 0.004 | 0.9715 | 0.0052 | 0.005 | 0.005 | 0.0042 | 0.0051 |
|  | MYb-5 | 0.0033 | 0.9737 | 0.0046 | 0.004 | 0.004 | 0.0054 | 0.005 |
|  | MYb-6 | 0.004 | 0.9747 | 0.0053 | 0.004 | 0.003 | 0.005 | 0.004 |
|  | MYb-7 | 0.0046 | 0.9555 | 0.0129 | 0.0036 | 0.0036 | 0.004 | 0.0158 |
|  | MYb-8 | 0.0031 | 0.9789 | 0.004 | 0.003 | 0.004 | 0.003 | 0.004 |
|  | MYb-9 | 0.003 | 0.9765 | 0.0046 | 0.0033 | 0.003 | 0.004 | 0.0056 |
|  | MYb-10 | 0.004 | 0.9704 | 0.0076 | 0.003 | 0.003 | 0.004 | 0.008 |
|  | MYb-11 | 0.0046 | 0.9704 | 0.005 | 0.0039 | 0.0034 | 0.0032 | 0.0095 |
|  | MYb-12 | 0.004 | 0.9629 | 0.0102 | 0.0037 | 0.004 | 0.0066 | 0.0086 |
|  | MYb-13 | 0.0064 | 0.9561 | 0.0083 | 0.004 | 0.0066 | 0.0086 | 0.01 |
|  | MYb-14 | 0.0036 | 0.974 | 0.0064 | 0.003 | 0.003 | 0.004 | 0.006 |
|  | MYb-15 | 0.0188 | 0.9274 | 0.02 | 0.0052 | 0.0036 | 0.0126 | 0.0124 |
|  | MYb-16 | 0.0037 | 0.9614 | 0.0161 | 0.0046 | 0.004 | 0.0046 | 0.0056 |
|  | MYb-17 | 0.0116 | 0.9624 | 0.0054 | 0.003 | 0.0056 | 0.007 | 0.005 |
| P5 | HZb-1 | 0.004 | 0.9709 | 0.0065 | 0.005 | 0.004 | 0.0056 | 0.004 |
|  | HZb-2 | 0.0046 | 0.9711 | 0.0059 | 0.004 | 0.0044 | 0.004 | 0.006 |
|  | HZb-3 | 0.0037 | 0.9777 | 0.0046 | 0.003 | 0.004 | 0.004 | 0.003 |
|  | HZb-4 | 0.0062 | 0.9042 | 0.0196 | 0.005 | 0.007 | 0.018 | 0.04 |
| P6 | PAb-1 | 0.005 | 0.9714 | 0.0046 | 0.003 | 0.003 | 0.005 | 0.008 |
|  | PAb-2 | 0.0073 | 0.9509 | 0.0088 | 0.008 | 0.0119 | 0.005 | 0.0081 |
|  | PAb-3 | 0.0053 | 0.975 | 0.0056 | 0.0034 | 0.003 | 0.0037 | 0.004 |
|  | PAb-4 | 0.0082 | 0.932 | 0.025 | 0.0097 | 0.0111 | 0.0094 | 0.0046 |
|  | PAb-5 | 0.004 | 0.9664 | 0.0106 | 0.004 | 0.003 | 0.004 | 0.008 |
|  | PAb-6 | 0.005 | 0.9272 | 0.016 | 0.0156 | 0.0183 | 0.0139 | 0.004 |
|  | PAb-7 | 0.004 | 0.9707 | 0.0064 | 0.0046 | 0.005 | 0.0046 | 0.0047 |
|  | PAb-8 | 0.004 | 0.9686 | 0.0064 | 0.004 | 0.0036 | 0.005 | 0.0084 |
|  | PAb-9 | 0.0046 | 0.9655 | 0.0058 | 0.003 | 0.0044 | 0.0049 | 0.0118 |
|  | PAb-10 | 0.006 | 0.9709 | 0.0064 | 0.003 | 0.0039 | 0.0036 | 0.0062 |
|  | PAb-11 | 0.007 | 0.8277 | 0.0259 | 0.0295 | 0.0767 | 0.0291 | 0.004 |
|  | PAb-12 | 0.003 | 0.9788 | 0.004 | 0.003 | 0.004 | 0.003 | 0.0042 |
|  | PAb-13 | 0.0201 | 0.8931 | 0.0194 | 0.0076 | 0.0072 | 0.0137 | 0.0389 |
|  | PAb-14 | 0.0032 | 0.9755 | 0.0046 | 0.004 | 0.004 | 0.0037 | 0.005 |
|  | PAb-15 | 0.0034 | 0.9716 | 0.0064 | 0.003 | 0.004 | 0.003 | 0.0086 |
|  | PAb-16 | 0.0088 | 0.933 | 0.0063 | 0.006 | 0.008 | 0.0286 | 0.0094 |
| P7 | XHb-1 | 0.005 | 0.9448 | 0.009 | 0.0202 | 0.006 | 0.0055 | 0.0096 |
|  | XHb-2 | 0.0055 | 0.8996 | 0.0221 | 0.0085 | 0.005 | 0.034 | 0.0254 |
|  | XHb-3 | 0.004 | 0.9727 | 0.0056 | 0.004 | 0.0034 | 0.0044 | 0.0059 |
|  | XHb-4 | 0.0083 | 0.9667 | 0.005 | 0.006 | 0.005 | 0.004 | 0.005 |
|  | XHb-5 | 0.0062 | 0.951 | 0.01 | 0.0058 | 0.004 | 0.0148 | 0.0082 |
|  | XHb-6 | 0.004 | 0.9673 | 0.0064 | 0.0076 | 0.005 | 0.0047 | 0.005 |
|  | XHb-7 | 0.004 | 0.858 | 0.1022 | 0.0056 | 0.0086 | 0.0102 | 0.0114 |
|  | XHb-8 | 0.003 | 0.9744 | 0.0042 | 0.003 | 0.0031 | 0.004 | 0.0083 |
|  | XHb-9 | 0.0054 | 0.8747 | 0.063 | 0.0087 | 0.0055 | 0.0304 | 0.0123 |
|  | XHb-10 | 0.0188 | 0.7628 | 0.1189 | 0.02 | 0.005 | 0.0684 | 0.0061 |
|  | XHb-11 | 0.0092 | 0.8952 | 0.0166 | 0.038 | 0.0088 | 0.02 | 0.0122 |
|  | XHb-12 | 0.003 | 0.9752 | 0.0048 | 0.0034 | 0.003 | 0.004 | 0.0066 |
|  | XHb-13 | 0.0103 | 0.9586 | 0.0086 | 0.0065 | 0.0056 | 0.0051 | 0.0053 |
|  | XHb-14 | 0.0039 | 0.9735 | 0.0063 | 0.0033 | 0.003 | 0.003 | 0.007 |
|  | XHb-15 | 0.004 | 0.9738 | 0.005 | 0.004 | 0.0043 | 0.004 | 0.0049 |
|  | XHb-16 | 0.0115 | 0.7871 | 0.0139 | 0.0823 | 0.0082 | 0.025 | 0.072 |
|  | XHb-17 | 0.0055 | 0.9543 | 0.0066 | 0.0121 | 0.0064 | 0.006 | 0.0091 |
| P8 | REG1b-1 | 0.0156 | 0.8968 | 0.0153 | 0.004 | 0.005 | 0.0182 | 0.0451 |
|  | REG1b-2 | 0.0046 | 0.8923 | 0.0514 | 0.0069 | 0.0082 | 0.0105 | 0.0261 |
|  | REG1b-3 | 0.0077 | 0.2906 | 0.5767 | 0.0809 | 0.016 | 0.0195 | 0.0086 |
|  | REG1b-4 | 0.0056 | 0.3968 | 0.4966 | 0.0064 | 0.0034 | 0.004 | 0.0873 |
|  | REG1b-5 | 0.0068 | 0.3762 | 0.5742 | 0.0076 | 0.0136 | 0.007 | 0.0146 |
|  | REG1b-6 | 0.0044 | 0.5093 | 0.4612 | 0.006 | 0.0066 | 0.007 | 0.0055 |
|  | REG1b-7 | 0.0041 | 0.4256 | 0.5422 | 0.0034 | 0.004 | 0.004 | 0.0167 |
|  | REG1b-8 | 0.006 | 0.3739 | 0.5707 | 0.0048 | 0.0038 | 0.004 | 0.0368 |
|  | REG1b-9 | 0.004 | 0.355 | 0.5119 | 0.0034 | 0.004 | 0.0034 | 0.1183 |
|  | REG1b-10 | 0.0074 | 0.3699 | 0.5807 | 0.0066 | 0.0091 | 0.0074 | 0.0189 |
|  | REG1b-11 | 0.0074 | 0.4636 | 0.4939 | 0.004 | 0.005 | 0.0045 | 0.0216 |
|  | REG1b-12 | 0.0074 | 0.4847 | 0.4543 | 0.0166 | 0.0089 | 0.0116 | 0.0165 |
| P9 | REG2b-1 | 0.0042 | 0.3842 | 0.5834 | 0.0055 | 0.0044 | 0.0099 | 0.0084 |
|  | REG2b-2 | 0.1954 | 0.3553 | 0.3726 | 0.0068 | 0.0125 | 0.0487 | 0.0086 |
|  | REG2b-3 | 0.0064 | 0.3007 | 0.5869 | 0.0432 | 0.02 | 0.0343 | 0.0085 |
|  | REG2b-4 | 0.0078 | 0.3601 | 0.5503 | 0.0082 | 0.0093 | 0.0114 | 0.0529 |
| P10 | REG3b-1 | 0.005 | 0.5983 | 0.3452 | 0.0053 | 0.0056 | 0.007 | 0.0336 |
|  | REG3b-2 | 0.006 | 0.6264 | 0.3201 | 0.0185 | 0.0124 | 0.0074 | 0.0092 |
|  | REG3b-3 | 0.008 | 0.4935 | 0.4462 | 0.003 | 0.0052 | 0.0056 | 0.0385 |
|  | REG3b-4 | 0.0094 | 0.391 | 0.5664 | 0.005 | 0.004 | 0.006 | 0.0182 |
|  | REG3b-5 | 0.0056 | 0.2651 | 0.6689 | 0.019 | 0.0131 | 0.0241 | 0.0042 |
|  | REG3b-6 | 0.0106 | 0.4395 | 0.5147 | 0.0113 | 0.0073 | 0.0094 | 0.0072 |
|  | REG3b-7 | 0.0406 | 0.3226 | 0.5428 | 0.0106 | 0.0257 | 0.0462 | 0.0114 |
|  | REG3b-8 | 0.0068 | 0.1321 | 0.7666 | 0.0419 | 0.0116 | 0.0364 | 0.0046 |
| P11 | BMb-1 | 0.0116 | 0.2246 | 0.7238 | 0.0098 | 0.0051 | 0.0211 | 0.004 |
|  | BMb-2 | 0.011 | 0.3053 | 0.62 | 0.0222 | 0.0165 | 0.0186 | 0.0064 |
|  | BMb-3 | 0.0047 | 0.3689 | 0.5856 | 0.0152 | 0.0078 | 0.01 | 0.0078 |
|  | BMb-4 | 0.0064 | 0.3829 | 0.566 | 0.0108 | 0.013 | 0.0159 | 0.005 |
| P12 | YSb-1 | 0.0314 | 0.0261 | 0.7652 | 0.0218 | 0.0145 | 0.0732 | 0.0678 |
|  | YSb-2 | 0.0326 | 0.0804 | 0.8217 | 0.0431 | 0.0098 | 0.0088 | 0.0035 |
|  | YSb-3 | 0.0233 | 0.1638 | 0.7752 | 0.016 | 0.0076 | 0.01 | 0.004 |
|  | YSb-4 | 0.0097 | 0.1861 | 0.769 | 0.0096 | 0.0064 | 0.01 | 0.0091 |
|  | YSb-5 | 0.0226 | 0.1536 | 0.7827 | 0.0112 | 0.0072 | 0.0115 | 0.0112 |
|  | YSb-6 | 0.0126 | 0.2141 | 0.5478 | 0.1011 | 0.0226 | 0.0934 | 0.0084 |
|  | YSb-7 | 0.0177 | 0.1358 | 0.7559 | 0.046 | 0.0087 | 0.0311 | 0.0048 |
|  | YSb-8 | 0.0378 | 0.0658 | 0.6324 | 0.0402 | 0.0125 | 0.1617 | 0.0496 |
|  | YSb-9 | 0.0088 | 0.007 | 0.2313 | 0.2326 | 0.0085 | 0.4189 | 0.0929 |
| S1 | MQa-1 | 0.0054 | 0.0074 | 0.0165 | 0.2187 | 0.021 | 0.013 | 0.718 |
|  | MQa-2 | 0.0054 | 0.0046 | 0.0162 | 0.008 | 0.0056 | 0.0264 | 0.9337 |
|  | MQa-3 | 0.0444 | 0.0068 | 0.0177 | 0.0122 | 0.01 | 0.0124 | 0.8965 |
|  | MQa-4 | 0.006 | 0.0126 | 0.0078 | 0.007 | 0.004 | 0.005 | 0.9576 |
|  | MQa-5 | 0.1816 | 0.0044 | 0.0235 | 0.0218 | 0.128 | 0.0403 | 0.6003 |
|  | MQa-6 | 0.0043 | 0.0068 | 0.0064 | 0.005 | 0.0128 | 0.005 | 0.9597 |
|  | MQa-7 | 0.0179 | 0.0076 | 0.0363 | 0.008 | 0.0091 | 0.0085 | 0.9126 |
|  | MQa-8 | 0.0572 | 0.0095 | 0.0127 | 0.0153 | 0.0655 | 0.1233 | 0.7164 |
|  | MQa-9 | 0.0522 | 0.0151 | 0.0252 | 0.0126 | 0.0613 | 0.0341 | 0.7995 |
|  | MQa-10 | 0.004 | 0.0076 | 0.0055 | 0.007 | 0.004 | 0.0057 | 0.9662 |
|  | MQa-11 | 0.0044 | 0.0064 | 0.0058 | 0.005 | 0.004 | 0.006 | 0.9684 |
|  | MQa-12 | 0.0109 | 0.0219 | 0.0074 | 0.0373 | 0.0226 | 0.014 | 0.8859 |
|  | MQa-13 | 0.0102 | 0.0247 | 0.0082 | 0.0593 | 0.0459 | 0.0403 | 0.8113 |
|  | MQa-14 | 0.0554 | 0.0177 | 0.0339 | 0.0382 | 0.1721 | 0.0538 | 0.6289 |
|  | MQa-15 | 0.0436 | 0.0057 | 0.0232 | 0.0136 | 0.0196 | 0.0648 | 0.8295 |
| S2 | DR1a-1 | 0.1966 | 0.0058 | 0.0123 | 0.0186 | 0.7211 | 0.0392 | 0.0064 |
|  | DR1a-2 | 0.0185 | 0.005 | 0.005 | 0.0256 | 0.5988 | 0.0088 | 0.3383 |
|  | DR1a-3 | 0.0436 | 0.0044 | 0.008 | 0.0131 | 0.829 | 0.0865 | 0.0154 |
|  | DR1a-4 | 0.0128 | 0.0275 | 0.0209 | 0.0151 | 0.7514 | 0.1607 | 0.0116 |
|  | DR1a-5 | 0.0267 | 0.005 | 0.0061 | 0.4301 | 0.5086 | 0.0163 | 0.0072 |
|  | DR1a-6 | 0.1285 | 0.015 | 0.0177 | 0.0248 | 0.7939 | 0.0117 | 0.0084 |
|  | DR1a-7 | 0.0176 | 0.004 | 0.0076 | 0.0915 | 0.6133 | 0.2141 | 0.0519 |
|  | DR1a-8 | 0.0182 | 0.0039 | 0.007 | 0.1603 | 0.7809 | 0.0221 | 0.0076 |
|  | DR1a-9 | 0.2587 | 0.0072 | 0.0129 | 0.0567 | 0.4778 | 0.1611 | 0.0256 |
| S3 | DR2a-1 | 0.0699 | 0.0132 | 0.0763 | 0.04 | 0.0251 | 0.1778 | 0.5978 |
|  | DR2a-2 | 0.0074 | 0.0193 | 0.008 | 0.035 | 0.0324 | 0.1322 | 0.7657 |
|  | DR2a-3 | 0.0042 | 0.005 | 0.0068 | 0.019 | 0.1202 | 0.0441 | 0.8007 |
|  | DR2a-4 | 0.085 | 0.005 | 0.0103 | 0.0145 | 0.2697 | 0.0783 | 0.5372 |
|  | DR2a-5 | 0.05 | 0.0091 | 0.0152 | 0.0228 | 0.3191 | 0.0938 | 0.4899 |
|  | DR2a-6 | 0.0468 | 0.1102 | 0.1091 | 0.007 | 0.0691 | 0.005 | 0.6528 |
|  | DR2a-7 | 0.0156 | 0.0054 | 0.0136 | 0.0239 | 0.0206 | 0.0128 | 0.9081 |
|  | DR2a-8 | 0.055 | 0.008 | 0.0074 | 0.0054 | 0.457 | 0.0085 | 0.4587 |
|  | DR2a-9 | 0.085 | 0.181 | 0.0107 | 0.006 | 0.0622 | 0.0152 | 0.6399 |
|  | DR2a-10 | 0.0164 | 0.006 | 0.0869 | 0.0266 | 0.0757 | 0.0116 | 0.7768 |
|  | DR2a-11 | 0.0293 | 0.1504 | 0.0289 | 0.0518 | 0.0184 | 0.0709 | 0.6502 |
|  | DR2a-12 | 0.0106 | 0.0066 | 0.0087 | 0.0165 | 0.0087 | 0.0074 | 0.9415 |
| S4 | QLa-1 | 0.0056 | 0.1652 | 0.0084 | 0.005 | 0.004 | 0.0074 | 0.8044 |
|  | QLa-2 | 0.0062 | 0.0067 | 0.0062 | 0.005 | 0.004 | 0.01 | 0.9619 |
|  | QLa-3 | 0.0064 | 0.015 | 0.0173 | 0.0062 | 0.0044 | 0.0054 | 0.9453 |
|  | QLa-4 | 0.005 | 0.0168 | 0.0092 | 0.005 | 0.005 | 0.006 | 0.953 |
|  | QLa-5 | 0.0046 | 0.0066 | 0.0044 | 0.005 | 0.005 | 0.005 | 0.9694 |
|  | QLa-6 | 0.0068 | 0.0118 | 0.0092 | 0.008 | 0.0064 | 0.006 | 0.9518 |
|  | QLa-7 | 0.004 | 0.0046 | 0.0036 | 0.003 | 0.003 | 0.0031 | 0.9787 |
|  | QLa-8 | 0.0046 | 0.0052 | 0.006 | 0.0079 | 0.0071 | 0.005 | 0.9642 |
|  | QLa-9 | 0.0046 | 0.012 | 0.006 | 0.005 | 0.0037 | 0.0124 | 0.9563 |
|  | QLa-10 | 0.0055 | 0.006 | 0.007 | 0.0135 | 0.0043 | 0.008 | 0.9557 |
|  | QLa-11 | 0.004 | 0.0054 | 0.0058 | 0.005 | 0.003 | 0.004 | 0.9728 |
|  | QLa-12 | 0.0049 | 0.004 | 0.0046 | 0.007 | 0.004 | 0.011 | 0.9645 |
|  | QLa-13 | 0.0086 | 0.0179 | 0.0129 | 0.0248 | 0.0062 | 0.7202 | 0.2094 |
|  | QLa-14 | 0.0046 | 0.004 | 0.0044 | 0.0081 | 0.003 | 0.0131 | 0.9628 |
|  | QLa-15 | 0.0044 | 0.004 | 0.0052 | 0.005 | 0.0046 | 0.0051 | 0.9717 |
| S5 | MYa-1 | 0.0052 | 0.0417 | 0.0098 | 0.0064 | 0.0094 | 0.006 | 0.9215 |
|  | MYa-2 | 0.0106 | 0.0411 | 0.0111 | 0.007 | 0.0318 | 0.0174 | 0.881 |
|  | MYa-3 | 0.005 | 0.0086 | 0.0051 | 0.0047 | 0.0051 | 0.008 | 0.9635 |
|  | MYa-4 | 0.008 | 0.0487 | 0.0079 | 0.008 | 0.0336 | 0.0151 | 0.8787 |
|  | MYa-5 | 0.0109 | 0.0054 | 0.0118 | 0.0187 | 0.0086 | 0.0226 | 0.922 |
|  | MYa-6 | 0.0057 | 0.0086 | 0.0053 | 0.004 | 0.004 | 0.007 | 0.9654 |
|  | MYa-7 | 0.0054 | 0.0062 | 0.0129 | 0.0123 | 0.0074 | 0.0108 | 0.945 |
|  | MYa-8 | 0.0058 | 0.0077 | 0.006 | 0.008 | 0.0058 | 0.007 | 0.9597 |
|  | MYa-9 | 0.0046 | 0.005 | 0.005 | 0.004 | 0.0036 | 0.006 | 0.9718 |
|  | MYa-10 | 0.0094 | 0.021 | 0.0084 | 0.012 | 0.0066 | 0.0207 | 0.9219 |
|  | MYa-11 | 0.0056 | 0.004 | 0.0052 | 0.004 | 0.004 | 0.008 | 0.9692 |
|  | MYa-12 | 0.0052 | 0.005 | 0.0058 | 0.003 | 0.004 | 0.0044 | 0.9726 |
|  | MYa-13 | 0.0054 | 0.006 | 0.0062 | 0.004 | 0.0072 | 0.005 | 0.9662 |
|  | MYa-14 | 0.0052 | 0.0056 | 0.005 | 0.0057 | 0.005 | 0.0056 | 0.9679 |
|  | MYa-15 | 0.0135 | 0.0064 | 0.0122 | 0.0153 | 0.0094 | 0.01 | 0.9332 |
|  | MYa-16 | 0.0087 | 0.0062 | 0.024 | 0.0074 | 0.0043 | 0.0081 | 0.9413 |
|  | MYa-17 | 0.0058 | 0.0044 | 0.0137 | 0.0247 | 0.005 | 0.0349 | 0.9115 |
|  | MYa-18 | 0.0067 | 0.0144 | 0.0142 | 0.0329 | 0.022 | 0.0488 | 0.861 |
|  | MYa-19 | 0.0056 | 0.008 | 0.0073 | 0.0076 | 0.0128 | 0.0171 | 0.9416 |
|  | MYa-20 | 0.004 | 0.0076 | 0.0046 | 0.007 | 0.005 | 0.006 | 0.9658 |
|  | MYa-21 | 0.004 | 0.005 | 0.0056 | 0.004 | 0.004 | 0.005 | 0.9724 |
|  | MYa-22 | 0.0284 | 0.0074 | 0.015 | 0.1112 | 0.0104 | 0.0176 | 0.81 |
|  | MYa-23 | 0.0103 | 0.024 | 0.0064 | 0.005 | 0.004 | 0.007 | 0.9433 |
|  | MYa-24 | 0.0058 | 0.004 | 0.0052 | 0.0054 | 0.0074 | 0.0056 | 0.9666 |
|  | MYa-25 | 0.0071 | 0.004 | 0.0056 | 0.007 | 0.005 | 0.0098 | 0.9615 |
| S6 | HZa-1 | 0.006 | 0.0056 | 0.0048 | 0.007 | 0.007 | 0.005 | 0.9646 |
|  | HZa-2 | 0.0246 | 0.0724 | 0.0307 | 0.0098 | 0.0289 | 0.0122 | 0.8214 |
|  | HZa-3 | 0.0058 | 0.004 | 0.004 | 0.004 | 0.004 | 0.004 | 0.9742 |
|  | HZa-4 | 0.0103 | 0.0284 | 0.0105 | 0.0076 | 0.0046 | 0.0128 | 0.9258 |
|  | HZa-5 | 0.0052 | 0.0118 | 0.0369 | 0.006 | 0.005 | 0.0104 | 0.9247 |
|  | HZa-6 | 0.005 | 0.005 | 0.0046 | 0.007 | 0.0036 | 0.0139 | 0.9609 |
|  | HZa-7 | 0.0169 | 0.0054 | 0.0258 | 0.0224 | 0.0122 | 0.0203 | 0.897 |
| S7 | PAa-1 | 0.0151 | 0.0094 | 0.0109 | 0.0094 | 0.0062 | 0.3011 | 0.6479 |
|  | PAa-2 | 0.0072 | 0.006 | 0.0079 | 0.009 | 0.0051 | 0.191 | 0.7738 |
|  | PAa-3 | 0.0062 | 0.0084 | 0.0274 | 0.0674 | 0.0094 | 0.0492 | 0.832 |
|  | PAa-4 | 0.0062 | 0.0068 | 0.018 | 0.007 | 0.004 | 0.0953 | 0.8627 |
|  | PAa-5 | 0.0046 | 0.0089 | 0.0126 | 0.0045 | 0.0047 | 0.006 | 0.9587 |
|  | PAa-6 | 0.005 | 0.0092 | 0.0144 | 0.0103 | 0.006 | 0.0146 | 0.9405 |
|  | PAa-7 | 0.004 | 0.0037 | 0.0109 | 0.005 | 0.1666 | 0.0156 | 0.7942 |
|  | PAa-8 | 0.0068 | 0.005 | 0.0105 | 0.0046 | 0.0035 | 0.0052 | 0.9644 |
|  | PAa-9 | 0.0087 | 0.0062 | 0.0078 | 0.004 | 0.004 | 0.0065 | 0.9628 |
|  | PAa-10 | 0.0046 | 0.006 | 0.0083 | 0.0077 | 0.005 | 0.0088 | 0.9596 |
|  | PAa-11 | 0.0052 | 0.0057 | 0.0059 | 0.0077 | 0.005 | 0.008 | 0.9625 |
|  | PAa-12 | 0.0058 | 0.008 | 0.006 | 0.0045 | 0.005 | 0.0077 | 0.963 |
|  | PAa-13 | 0.0136 | 0.0082 | 0.0214 | 0.0095 | 0.0436 | 0.0136 | 0.8901 |
|  | PAa-14 | 0.0312 | 0.005 | 0.007 | 0.0096 | 0.0377 | 0.0064 | 0.903 |
|  | PAa-15 | 0.0046 | 0.014 | 0.0052 | 0.004 | 0.0056 | 0.005 | 0.9616 |
|  | PAa-16 | 0.0121 | 0.0071 | 0.0303 | 0.0065 | 0.0097 | 0.0335 | 0.9008 |
|  | PAa-17 | 0.0142 | 0.0054 | 0.0765 | 0.0188 | 0.0075 | 0.008 | 0.8695 |
|  | PAa-18 | 0.0137 | 0.0128 | 0.0185 | 0.0105 | 0.0074 | 0.007 | 0.9301 |
|  | PAa-19 | 0.0151 | 0.0058 | 0.0646 | 0.0353 | 0.006 | 0.0193 | 0.8539 |
|  | PAa-20 | 0.0068 | 0.015 | 0.0279 | 0.0054 | 0.004 | 0.012 | 0.9289 |
|  | PAa-21 | 0.006 | 0.0379 | 0.0099 | 0.0076 | 0.0036 | 0.0056 | 0.9294 |
| S8 | XHa-1 | 0.0056 | 0.01 | 0.0915 | 0.006 | 0.004 | 0.6779 | 0.205 |
|  | XHa-2 | 0.003 | 0.014 | 0.0058 | 0.004 | 0.0041 | 0.0041 | 0.965 |
|  | XHa-3 | 0.0055 | 0.0203 | 0.0082 | 0.004 | 0.0072 | 0.0815 | 0.8732 |
|  | XHa-4 | 0.1356 | 0.0671 | 0.2803 | 0.0068 | 0.0036 | 0.0448 | 0.4618 |
|  | XHa-5 | 0.0059 | 0.006 | 0.0143 | 0.3832 | 0.005 | 0.044 | 0.5416 |
|  | XHa-6 | 0.015 | 0.0117 | 0.0066 | 0.004 | 0.0434 | 0.0064 | 0.9129 |
|  | XHa-7 | 0.0062 | 0.007 | 0.0181 | 0.0228 | 0.004 | 0.9199 | 0.022 |
|  | XHa-8 | 0.0076 | 0.0099 | 0.0083 | 0.006 | 0.004 | 0.006 | 0.9582 |
|  | XHa-9 | 0.0086 | 0.0216 | 0.0272 | 0.0086 | 0.005 | 0.0318 | 0.8972 |
|  | XHa-10 | 0.008 | 0.0065 | 0.0107 | 0.0111 | 0.004 | 0.045 | 0.9147 |
|  | XHa-11 | 0.0085 | 0.0112 | 0.0104 | 0.0354 | 0.0072 | 0.8032 | 0.1241 |
|  | XHa-12 | 0.0057 | 0.0044 | 0.0943 | 0.0459 | 0.005 | 0.2677 | 0.577 |
|  | XHa-13 | 0.0066 | 0.0086 | 0.0306 | 0.01 | 0.0168 | 0.1505 | 0.7769 |
|  | XHa-14 | 0.0064 | 0.0873 | 0.0344 | 0.0066 | 0.006 | 0.0623 | 0.7969 |
|  | XHa-15 | 0.0205 | 0.0074 | 0.0237 | 0.004 | 0.0046 | 0.5514 | 0.3884 |
|  | XHa-16 | 0.01 | 0.015 | 0.0195 | 0.005 | 0.0032 | 0.0416 | 0.9057 |
|  | XHa-17 | 0.0113 | 0.0201 | 0.0439 | 0.0169 | 0.0214 | 0.3063 | 0.5802 |
|  | XHa-18 | 0.004 | 0.0118 | 0.015 | 0.004 | 0.0054 | 0.0076 | 0.9522 |
| S9 | REG1a-1 | 0.0052 | 0.005 | 0.008 | 0.0181 | 0.0181 | 0.9268 | 0.0188 |
|  | REG1a-2 | 0.1649 | 0.005 | 0.1976 | 0.0474 | 0.0084 | 0.4929 | 0.0839 |
|  | REG1a-3 | 0.0217 | 0.012 | 0.0663 | 0.0175 | 0.005 | 0.0413 | 0.8362 |
|  | REG1a-4 | 0.0534 | 0.006 | 0.0936 | 0.0139 | 0.0066 | 0.1991 | 0.6274 |
|  | REG1a-5 | 0.0307 | 0.0054 | 0.0474 | 0.0358 | 0.004 | 0.8562 | 0.0205 |
|  | REG1a-6 | 0.1328 | 0.1794 | 0.1699 | 0.006 | 0.0086 | 0.2942 | 0.2091 |
|  | REG1a-7 | 0.0273 | 0.0171 | 0.0379 | 0.0094 | 0.0068 | 0.047 | 0.8545 |
|  | REG1a-8 | 0.0066 | 0.0079 | 0.0258 | 0.005 | 0.0032 | 0.0788 | 0.8727 |
|  | REG1a-9 | 0.013 | 0.1085 | 0.1405 | 0.0486 | 0.0116 | 0.1865 | 0.4914 |
| S10 | REG2a-2 | 0.0146 | 0.0097 | 0.0233 | 0.009 | 0.0065 | 0.9146 | 0.0223 |
|  | REG2a-3 | 0.012 | 0.005 | 0.0281 | 0.0128 | 0.0061 | 0.1537 | 0.7823 |
|  | REG2a-4 | 0.0178 | 0.0091 | 0.0545 | 0.0546 | 0.0191 | 0.7826 | 0.0624 |
|  | REG2a-5 | 0.0258 | 0.0363 | 0.0198 | 0.0266 | 0.0133 | 0.6264 | 0.2518 |
|  | REG2a-6 | 0.0662 | 0.0083 | 0.1241 | 0.0072 | 0.0036 | 0.0415 | 0.749 |
|  | REG2a-7 | 0.0431 | 0.054 | 0.038 | 0.0086 | 0.0056 | 0.3122 | 0.5385 |
|  | REG2a-8 | 0.041 | 0.006 | 0.056 | 0.0044 | 0.004 | 0.7288 | 0.1599 |
|  | REG2a-9 | 0.0509 | 0.007 | 0.0593 | 0.0391 | 0.0055 | 0.6804 | 0.1577 |
|  | REG2a-10 | 0.0072 | 0.0046 | 0.0086 | 0.0196 | 0.0061 | 0.9417 | 0.0122 |
|  | REG2a-11 | 0.0293 | 0.0133 | 0.0311 | 0.006 | 0.008 | 0.8725 | 0.0398 |
|  | REG2a-12 | 0.034 | 0.013 | 0.1429 | 0.0223 | 0.0385 | 0.1245 | 0.6247 |
| S11 | REG3a-3 | 0.011 | 0.006 | 0.0108 | 0.0069 | 0.0069 | 0.8933 | 0.0652 |
|  | REG3a-4 | 0.0091 | 0.005 | 0.0141 | 0.006 | 0.005 | 0.9129 | 0.0479 |
|  | REG3a-5 | 0.0311 | 0.0097 | 0.0787 | 0.0128 | 0.0517 | 0.7493 | 0.0667 |
|  | REG3a-6 | 0.0483 | 0.0054 | 0.0413 | 0.0195 | 0.0405 | 0.7178 | 0.1271 |
|  | REG3a-7 | 0.0064 | 0.0123 | 0.006 | 0.0126 | 0.0363 | 0.9103 | 0.0162 |
|  | REG3a-8 | 0.0137 | 0.0049 | 0.0985 | 0.0314 | 0.0135 | 0.1545 | 0.6835 |
|  | REG3a-9 | 0.0072 | 0.0064 | 0.0621 | 0.1047 | 0.0084 | 0.8013 | 0.0099 |
|  | REG3a-10 | 0.0255 | 0.0074 | 0.137 | 0.0444 | 0.014 | 0.7398 | 0.0319 |
|  | REG3a-11 | 0.0114 | 0.01 | 0.009 | 0.0113 | 0.0162 | 0.8892 | 0.0528 |
|  | REG3a-12 | 0.0051 | 0.005 | 0.0124 | 0.01 | 0.0097 | 0.9516 | 0.0062 |
|  | REG3a-13 | 0.005 | 0.0085 | 0.0067 | 0.0217 | 0.007 | 0.8973 | 0.0538 |
|  | REG3a-14 | 0.011 | 0.0047 | 0.0092 | 0.4696 | 0.0166 | 0.4765 | 0.0125 |
|  | REG3a-15 | 0.0216 | 0.0062 | 0.0101 | 0.006 | 0.0086 | 0.9334 | 0.0141 |
|  | REG3a-16 | 0.0082 | 0.0055 | 0.0178 | 0.005 | 0.0064 | 0.948 | 0.0091 |
|  | REG3a-17 | 0.0066 | 0.0064 | 0.0147 | 0.0112 | 0.005 | 0.9427 | 0.0134 |
| S12 | HYa-1 | 0.0111 | 0.0054 | 0.0177 | 0.0229 | 0.0115 | 0.9083 | 0.0231 |
|  | HYa-2 | 0.1313 | 0.003 | 0.0302 | 0.0383 | 0.3067 | 0.482 | 0.0084 |
|  | HYa-3 | 0.0066 | 0.0204 | 0.0095 | 0.0638 | 0.1201 | 0.7491 | 0.0305 |
|  | HYa-4 | 0.0135 | 0.0082 | 0.0448 | 0.5478 | 0.2245 | 0.1463 | 0.0149 |
|  | HYa-5 | 0.0177 | 0.0089 | 0.0184 | 0.0284 | 0.0143 | 0.7079 | 0.2044 |
|  | HYa-6 | 0.0088 | 0.0058 | 0.0138 | 0.0174 | 0.0082 | 0.9272 | 0.0188 |
|  | HYa-7 | 0.0066 | 0.0079 | 0.0137 | 0.2827 | 0.1151 | 0.5658 | 0.0082 |
|  | HYa-8 | 0.0222 | 0.0064 | 0.1002 | 0.6662 | 0.0194 | 0.1051 | 0.0805 |
|  | HYa-9 | 0.0504 | 0.004 | 0.0127 | 0.1804 | 0.0949 | 0.6508 | 0.0067 |
|  | HYa-10 | 0.0094 | 0.0054 | 0.015 | 0.0115 | 0.0087 | 0.9436 | 0.0064 |
|  | HYa-11 | 0.0459 | 0.0217 | 0.0094 | 0.0145 | 0.0187 | 0.8802 | 0.0096 |
|  | HYa-12 | 0.0723 | 0.004 | 0.151 | 0.0081 | 0.0111 | 0.7414 | 0.0121 |
|  | HYa-13 | 0.0327 | 0.0044 | 0.1674 | 0.0272 | 0.0129 | 0.749 | 0.0065 |
|  | HYa-14 | 0.0157 | 0.0153 | 0.0491 | 0.0285 | 0.0163 | 0.8165 | 0.0586 |
| S13 | DQINa-1 | 0.5527 | 0.005 | 0.3812 | 0.0064 | 0.0074 | 0.0287 | 0.0186 |
|  | DQINa-2 | 0.5617 | 0.0064 | 0.391 | 0.0134 | 0.0058 | 0.0086 | 0.0131 |
|  | DQINa-3 | 0.5785 | 0.0055 | 0.3865 | 0.0062 | 0.0046 | 0.0117 | 0.007 |
|  | DQINa-4 | 0.5459 | 0.0078 | 0.3883 | 0.0134 | 0.014 | 0.0111 | 0.0195 |
|  | DQINa-5 | 0.5783 | 0.004 | 0.3858 | 0.0105 | 0.0084 | 0.0056 | 0.0074 |
|  | DQINa-6 | 0.6128 | 0.0106 | 0.2995 | 0.0309 | 0.0189 | 0.014 | 0.0133 |
|  | DQINa-7 | 0.568 | 0.0054 | 0.3834 | 0.005 | 0.004 | 0.0105 | 0.0237 |
|  | DQINa-8 | 0.5795 | 0.004 | 0.3914 | 0.0052 | 0.0052 | 0.009 | 0.0057 |
|  | DQINa-9 | 0.5855 | 0.004 | 0.3921 | 0.003 | 0.0046 | 0.0052 | 0.0056 |
|  | DQINa-10 | 0.4417 | 0.0085 | 0.3846 | 0.0046 | 0.0102 | 0.0755 | 0.0748 |
|  | DQINa-11 | 0.4649 | 0.0136 | 0.4508 | 0.0062 | 0.004 | 0.0169 | 0.0436 |
|  | DQINa-12 | 0.4926 | 0.0276 | 0.43 | 0.007 | 0.0161 | 0.0139 | 0.0128 |
|  | DQINa-13 | 0.4912 | 0.0056 | 0.3979 | 0.0174 | 0.0076 | 0.0595 | 0.0207 |
|  | DQINa-14 | 0.5781 | 0.0036 | 0.3818 | 0.0047 | 0.0036 | 0.0156 | 0.0126 |
|  | DQINa-15 | 0.4862 | 0.0704 | 0.3593 | 0.0099 | 0.0235 | 0.0289 | 0.0218 |
|  | DQINa-16 | 0.5616 | 0.0076 | 0.3825 | 0.0062 | 0.0122 | 0.0061 | 0.0238 |
|  | DQINa-17 | 0.5827 | 0.003 | 0.3906 | 0.0056 | 0.0036 | 0.0062 | 0.0083 |
|  | DQINa-18 | 0.5852 | 0.003 | 0.392 | 0.0059 | 0.0041 | 0.0046 | 0.0052 |
|  | DQINa-19 | 0.5063 | 0.013 | 0.3735 | 0.0182 | 0.0088 | 0.0245 | 0.0558 |
|  | DQINa-20 | 0.5626 | 0.0094 | 0.3854 | 0.0076 | 0.0045 | 0.015 | 0.0155 |
|  | DQINa-21 | 0.5565 | 0.0185 | 0.3007 | 0.0398 | 0.0059 | 0.067 | 0.0116 |
|  | DQINa-22 | 0.351 | 0.0068 | 0.3743 | 0.0283 | 0.01 | 0.2184 | 0.0112 |
|  | DQINa-23 | 0.5302 | 0.0052 | 0.3784 | 0.0182 | 0.0052 | 0.0522 | 0.0106 |
|  | DQINa-24 | 0.6216 | 0.0058 | 0.3151 | 0.0046 | 0.0235 | 0.0094 | 0.02 |
|  | DQINa-25 | 0.66 | 0.006 | 0.1951 | 0.0521 | 0.0185 | 0.0064 | 0.0618 |
| S14 | DCa-1 | 0.9123 | 0.0098 | 0.005 | 0.029 | 0.0118 | 0.0178 | 0.0142 |
|  | DCa-2 | 0.9488 | 0.0115 | 0.0072 | 0.004 | 0.0056 | 0.007 | 0.0159 |
|  | DCa-3 | 0.9573 | 0.0066 | 0.012 | 0.0072 | 0.0058 | 0.0064 | 0.0047 |
|  | DCa-4 | 0.8609 | 0.0198 | 0.0347 | 0.011 | 0.05 | 0.0162 | 0.0074 |
|  | DCa-5 | 0.9626 | 0.004 | 0.0072 | 0.0092 | 0.0056 | 0.0064 | 0.005 |
|  | DCa-6 | 0.9121 | 0.0193 | 0.0218 | 0.012 | 0.0069 | 0.0229 | 0.005 |
|  | DCa-7 | 0.9392 | 0.003 | 0.0061 | 0.0108 | 0.0111 | 0.0221 | 0.0076 |
|  | DCa-8 | 0.8307 | 0.0197 | 0.0072 | 0.0075 | 0.105 | 0.0189 | 0.0109 |
|  | DCa-9 | 0.5225 | 0.069 | 0.0192 | 0.1699 | 0.1968 | 0.0076 | 0.015 |
|  | DCa-10 | 0.8952 | 0.0044 | 0.0408 | 0.0275 | 0.0054 | 0.0156 | 0.0112 |
|  | DCa-11 | 0.9552 | 0.0156 | 0.0058 | 0.0046 | 0.0084 | 0.0064 | 0.004 |
|  | DCa-12 | 0.8008 | 0.0089 | 0.0128 | 0.0258 | 0.1192 | 0.0206 | 0.012 |
|  | DCa-13 | 0.894 | 0.0052 | 0.0473 | 0.0041 | 0.004 | 0.0192 | 0.0262 |
|  | DCa-14 | 0.9126 | 0.0078 | 0.0119 | 0.0362 | 0.0199 | 0.0054 | 0.0062 |
|  | DCa-15 | 0.9555 | 0.0086 | 0.0075 | 0.0049 | 0.0056 | 0.0049 | 0.013 |
|  | DCa-16 | 0.8831 | 0.0158 | 0.0269 | 0.0104 | 0.0072 | 0.0386 | 0.018 |
|  | DCa-17 | 0.9435 | 0.005 | 0.0105 | 0.0103 | 0.0081 | 0.017 | 0.0056 |
|  | DCa-18 | 0.9522 | 0.0092 | 0.0112 | 0.004 | 0.01 | 0.0054 | 0.008 |
|  | DCa-19 | 0.9253 | 0.0064 | 0.0114 | 0.0108 | 0.0368 | 0.005 | 0.0043 |
|  | DCa-20 | 0.6046 | 0.2106 | 0.0097 | 0.0098 | 0.1117 | 0.0188 | 0.0348 |
|  | DCa-21 | 0.8452 | 0.0067 | 0.0158 | 0.01 | 0.0295 | 0.0348 | 0.058 |
|  | DCa-22 | 0.8979 | 0.0114 | 0.0147 | 0.0342 | 0.007 | 0.029 | 0.0058 |
|  | DCa-23 | 0.7639 | 0.0358 | 0.032 | 0.0149 | 0.0076 | 0.008 | 0.1378 |
|  | DCa-24 | 0.9277 | 0.004 | 0.0118 | 0.0096 | 0.0227 | 0.0086 | 0.0156 |
|  | DCa-25 | 0.9549 | 0.0076 | 0.0058 | 0.0065 | 0.0094 | 0.0106 | 0.0052 |
|  | DCa-26 | 0.9511 | 0.0046 | 0.0052 | 0.0094 | 0.0068 | 0.0103 | 0.0126 |
|  | DCa-27 | 0.9074 | 0.0053 | 0.062 | 0.0106 | 0.0037 | 0.006 | 0.005 |
|  | DCa-28 | 0.9592 | 0.0058 | 0.0081 | 0.0052 | 0.0082 | 0.0074 | 0.0061 |
|  | DCa-29 | 0.9702 | 0.003 | 0.0051 | 0.0055 | 0.0074 | 0.0058 | 0.003 |
| S15 | LTa-1 | 0.955 | 0.0042 | 0.0154 | 0.007 | 0.0076 | 0.004 | 0.0068 |
|  | LTa-2 | 0.9615 | 0.005 | 0.0082 | 0.0072 | 0.005 | 0.0097 | 0.0034 |
|  | LTa-3 | 0.9542 | 0.0044 | 0.0146 | 0.0064 | 0.0082 | 0.005 | 0.0072 |
|  | LTa-4 | 0.9069 | 0.004 | 0.0301 | 0.0064 | 0.0066 | 0.042 | 0.004 |
|  | LTa-5 | 0.8107 | 0.0053 | 0.1188 | 0.004 | 0.0114 | 0.0385 | 0.0113 |
|  | LTa-6 | 0.916 | 0.0056 | 0.0076 | 0.0142 | 0.0304 | 0.0217 | 0.0045 |
|  | LTa-7 | 0.5977 | 0.0083 | 0.2288 | 0.0069 | 0.0521 | 0.0945 | 0.0117 |
|  | LTa-8 | 0.9528 | 0.0064 | 0.0114 | 0.004 | 0.0104 | 0.0064 | 0.0086 |
|  | LTa-9 | 0.9657 | 0.004 | 0.0052 | 0.004 | 0.0071 | 0.007 | 0.007 |
|  | LTa-10 | 0.6878 | 0.015 | 0.0275 | 0.0577 | 0.0062 | 0.1949 | 0.0108 |
|  | LTa-11 | 0.9456 | 0.004 | 0.0062 | 0.0086 | 0.0244 | 0.0062 | 0.005 |
|  | LTa-12 | 0.9215 | 0.0123 | 0.0222 | 0.0116 | 0.0072 | 0.014 | 0.0112 |
|  | LTa-13 | 0.9244 | 0.0236 | 0.028 | 0.0066 | 0.008 | 0.0056 | 0.0038 |
|  | LTa-14 | 0.9244 | 0.0157 | 0.0155 | 0.0108 | 0.017 | 0.0094 | 0.0072 |
|  | LTa-15 | 0.9168 | 0.004 | 0.0074 | 0.0172 | 0.015 | 0.0256 | 0.014 |
|  | LTa-16 | 0.6898 | 0.0118 | 0.172 | 0.0285 | 0.0752 | 0.0148 | 0.0079 |
|  | LTa-17 | 0.3937 | 0.0201 | 0.3811 | 0.0393 | 0.0328 | 0.0813 | 0.0517 |
|  | LTa-18 | 0.89 | 0.0086 | 0.0106 | 0.0447 | 0.024 | 0.013 | 0.0091 |
|  | LTa-19 | 0.936 | 0.0052 | 0.0266 | 0.0118 | 0.011 | 0.0053 | 0.0041 |
|  | LTa-20 | 0.8978 | 0.0132 | 0.0109 | 0.0097 | 0.019 | 0.0164 | 0.033 |
|  | LTa-21 | 0.824 | 0.038 | 0.0837 | 0.005 | 0.0046 | 0.0084 | 0.0364 |
|  | LTa-22 | 0.9566 | 0.0052 | 0.0047 | 0.0095 | 0.0125 | 0.0069 | 0.0046 |
| S16 | GZa-1 | 0.4524 | 0.006 | 0.0179 | 0.0237 | 0.4739 | 0.0205 | 0.0056 |
|  | GZa-2 | 0.0245 | 0.0039 | 0.0055 | 0.0064 | 0.9407 | 0.01 | 0.009 |
|  | GZa-3 | 0.9494 | 0.003 | 0.0046 | 0.0046 | 0.025 | 0.0072 | 0.0062 |
|  | GZa-4 | 0.0157 | 0.0092 | 0.0152 | 0.0203 | 0.9168 | 0.0096 | 0.0132 |
|  | GZa-5 | 0.5007 | 0.0103 | 0.0176 | 0.1315 | 0.132 | 0.1693 | 0.0386 |
|  | GZa-6 | 0.6754 | 0.0076 | 0.0848 | 0.0417 | 0.061 | 0.0891 | 0.0404 |
|  | GZa-7 | 0.146 | 0.0192 | 0.0837 | 0.0169 | 0.1457 | 0.1136 | 0.4749 |
|  | GZa-8 | 0.1172 | 0.0126 | 0.0206 | 0.0265 | 0.7517 | 0.0463 | 0.0252 |
|  | GZa-9 | 0.7273 | 0.0226 | 0.1219 | 0.0187 | 0.067 | 0.0265 | 0.016 |
|  | GZa-10 | 0.5404 | 0.0713 | 0.0087 | 0.0071 | 0.3309 | 0.0166 | 0.025 |
|  | GZa-11 | 0.4164 | 0.0092 | 0.019 | 0.0074 | 0.5197 | 0.0164 | 0.012 |
|  | GZa-12 | 0.5031 | 0.009 | 0.0135 | 0.0103 | 0.4304 | 0.0249 | 0.0088 |
|  | GZa-13 | 0.0925 | 0.0888 | 0.014 | 0.018 | 0.7588 | 0.0118 | 0.0161 |
|  | GZa-14 | 0.5294 | 0.0108 | 0.17 | 0.0099 | 0.234 | 0.0193 | 0.0265 |
|  | GZa-15 | 0.2899 | 0.0044 | 0.0082 | 0.0079 | 0.6621 | 0.0138 | 0.0137 |
|  | GZa-16 | 0.6038 | 0.005 | 0.0078 | 0.0543 | 0.295 | 0.0267 | 0.0073 |
|  | GZa-17 | 0.2006 | 0.0054 | 0.0136 | 0.0136 | 0.6727 | 0.0868 | 0.0073 |
|  | GZa-18 | 0.0582 | 0.0064 | 0.0086 | 0.0279 | 0.0287 | 0.773 | 0.0971 |
|  | GZa-19 | 0.4923 | 0.006 | 0.0144 | 0.02 | 0.1326 | 0.151 | 0.1837 |
|  | GZa-20 | 0.7211 | 0.0116 | 0.0096 | 0.0305 | 0.1695 | 0.017 | 0.0407 |
|  | GZa-21 | 0.0686 | 0.0093 | 0.0178 | 0.0622 | 0.7119 | 0.1228 | 0.0074 |
|  | GZa-22 | 0.042 | 0.0093 | 0.0142 | 0.0564 | 0.7939 | 0.0405 | 0.0438 |
|  | GZa-23 | 0.1332 | 0.057 | 0.0162 | 0.0157 | 0.7435 | 0.0132 | 0.0212 |
|  | GZa-24 | 0.1493 | 0.0112 | 0.2525 | 0.0301 | 0.3852 | 0.0282 | 0.1434 |
| S17 | DFa-1 | 0.5677 | 0.0114 | 0.014 | 0.0089 | 0.3616 | 0.0235 | 0.0129 |
|  | DFa-2 | 0.615 | 0.025 | 0.0245 | 0.0088 | 0.3042 | 0.0163 | 0.0062 |
|  | DFa-3 | 0.5781 | 0.0094 | 0.0162 | 0.1779 | 0.1903 | 0.0219 | 0.0062 |
|  | DFa-4 | 0.0484 | 0.0054 | 0.0078 | 0.0343 | 0.8868 | 0.0133 | 0.0041 |
|  | DFa-5 | 0.3775 | 0.009 | 0.0089 | 0.0671 | 0.2769 | 0.2272 | 0.0333 |
|  | DFa-6 | 0.9008 | 0.0064 | 0.0573 | 0.0094 | 0.0071 | 0.0094 | 0.0096 |
|  | DFa-7 | 0.1041 | 0.004 | 0.0807 | 0.0795 | 0.1327 | 0.5874 | 0.0117 |
|  | DFa-8 | 0.8414 | 0.0054 | 0.009 | 0.0092 | 0.1218 | 0.0056 | 0.0076 |
|  | DFa-9 | 0.5249 | 0.0052 | 0.0212 | 0.015 | 0.3992 | 0.0169 | 0.0176 |
|  | DFa-10 | 0.8892 | 0.0118 | 0.0234 | 0.0084 | 0.0281 | 0.032 | 0.0071 |
|  | DFa-11 | 0.4784 | 0.0144 | 0.0321 | 0.0087 | 0.0739 | 0.2527 | 0.1398 |
|  | DFa-12 | 0.4038 | 0.0283 | 0.0138 | 0.0076 | 0.3257 | 0.0488 | 0.172 |
|  | DFa-13 | 0.962 | 0.0034 | 0.0092 | 0.0046 | 0.0118 | 0.005 | 0.004 |
|  | DFa-14 | 0.1013 | 0.0184 | 0.1167 | 0.0117 | 0.7238 | 0.0114 | 0.0167 |
|  | DFa-15 | 0.8403 | 0.0612 | 0.0372 | 0.0102 | 0.0271 | 0.0099 | 0.0141 |
|  | DFa-16 | 0.5457 | 0.0165 | 0.0108 | 0.0189 | 0.3907 | 0.009 | 0.0084 |
|  | DFa-17 | 0.7021 | 0.005 | 0.0193 | 0.2292 | 0.0075 | 0.0299 | 0.007 |
|  | DFa-18 | 0.4166 | 0.0049 | 0.1724 | 0.0516 | 0.016 | 0.3307 | 0.0079 |
|  | DFa-19 | 0.9121 | 0.0054 | 0.0403 | 0.0113 | 0.0183 | 0.0066 | 0.006 |
| S18 | DBa-1 | 0.1931 | 0.0044 | 0.1454 | 0.0175 | 0.007 | 0.6257 | 0.007 |
|  | DBa-2 | 0.1106 | 0.0074 | 0.2955 | 0.382 | 0.0257 | 0.151 | 0.0277 |
|  | DBa-3 | 0.7754 | 0.008 | 0.0233 | 0.0861 | 0.0092 | 0.0682 | 0.0298 |
|  | DBa-4 | 0.1066 | 0.006 | 0.2876 | 0.2309 | 0.0908 | 0.0792 | 0.1989 |
|  | DBa-5 | 0.07 | 0.0072 | 0.1916 | 0.6097 | 0.0257 | 0.0811 | 0.0146 |
|  | DBa-6 | 0.1719 | 0.003 | 0.1634 | 0.5299 | 0.0149 | 0.1061 | 0.0108 |
|  | DBa-7 | 0.0095 | 0.0231 | 0.1971 | 0.6255 | 0.0183 | 0.1021 | 0.0244 |
|  | DBa-8 | 0.0766 | 0.0912 | 0.1362 | 0.4006 | 0.0397 | 0.2237 | 0.0321 |
|  | DBa-9 | 0.0398 | 0.0217 | 0.0614 | 0.8468 | 0.0132 | 0.0121 | 0.005 |
|  | DBa-10 | 0.8009 | 0.0052 | 0.0633 | 0.01 | 0.0354 | 0.0777 | 0.0076 |
|  | DBa-11 | 0.1559 | 0.2064 | 0.1112 | 0.0122 | 0.0221 | 0.4812 | 0.011 |
|  | DBa-12 | 0.0239 | 0.0199 | 0.0777 | 0.033 | 0.0334 | 0.8061 | 0.006 |
|  | DBa-13 | 0.0675 | 0.006 | 0.3466 | 0.2088 | 0.046 | 0.2973 | 0.0279 |
|  | DBa-14 | 0.2214 | 0.0046 | 0.3656 | 0.1974 | 0.0167 | 0.1867 | 0.0075 |
|  | DBa-15 | 0.1994 | 0.007 | 0.3264 | 0.4353 | 0.005 | 0.0178 | 0.0091 |
|  | DBa-16 | 0.2903 | 0.0056 | 0.0644 | 0.0502 | 0.0245 | 0.1114 | 0.4535 |
| S19 | LHa-1 | 0.012 | 0.0058 | 0.0607 | 0.8861 | 0.0101 | 0.0203 | 0.005 |
|  | LHa-2 | 0.0056 | 0.0102 | 0.0056 | 0.9494 | 0.0151 | 0.0101 | 0.004 |
|  | LHa-3 | 0.0176 | 0.0098 | 0.0115 | 0.9314 | 0.0111 | 0.0096 | 0.009 |
|  | LHa-4 | 0.0052 | 0.003 | 0.0052 | 0.8948 | 0.0459 | 0.0419 | 0.004 |
|  | LHa-5 | 0.2748 | 0.0036 | 0.2803 | 0.2664 | 0.0229 | 0.1475 | 0.0046 |
|  | LHa-6 | 0.0082 | 0.005 | 0.009 | 0.5054 | 0.316 | 0.1505 | 0.006 |
|  | LHa-7 | 0.7883 | 0.0054 | 0.1695 | 0.0074 | 0.004 | 0.0116 | 0.0138 |
|  | LHa-8 | 0.0152 | 0.006 | 0.0074 | 0.0059 | 0.0052 | 0.9509 | 0.0094 |
|  | LHa-9 | 0.0578 | 0.0038 | 0.0294 | 0.0216 | 0.0077 | 0.8757 | 0.004 |
|  | LHa-10 | 0.0496 | 0.0091 | 0.0776 | 0.6691 | 0.0325 | 0.1523 | 0.0099 |
|  | LHa-11 | 0.0639 | 0.0054 | 0.0184 | 0.8008 | 0.0531 | 0.0519 | 0.0066 |
|  | LHa-12 | 0.0054 | 0.006 | 0.0077 | 0.9471 | 0.0208 | 0.006 | 0.007 |
|  | LHa-13 | 0.0741 | 0.0047 | 0.1166 | 0.7211 | 0.0092 | 0.0555 | 0.0188 |
|  | LHa-14 | 0.0143 | 0.0037 | 0.0088 | 0.58 | 0.2462 | 0.1386 | 0.0085 |
| S20 | ABa-1 | 0.0065 | 0.0074 | 0.01 | 0.9436 | 0.0095 | 0.017 | 0.006 |
|  | ABa-2 | 0.013 | 0.0176 | 0.0416 | 0.7755 | 0.0069 | 0.1394 | 0.006 |
|  | ABa-3 | 0.0573 | 0.0132 | 0.1439 | 0.0725 | 0.0232 | 0.685 | 0.005 |
|  | ABa-4 | 0.0496 | 0.0044 | 0.0892 | 0.049 | 0.005 | 0.7973 | 0.0054 |
|  | ABa-5 | 0.0058 | 0.0054 | 0.0075 | 0.01 | 0.0044 | 0.9612 | 0.0057 |
|  | ABa-6 | 0.1958 | 0.008 | 0.1262 | 0.0173 | 0.0089 | 0.6394 | 0.0044 |
|  | ABa-7 | 0.0086 | 0.0076 | 0.0166 | 0.0325 | 0.019 | 0.8826 | 0.0331 |
|  | ABa-8 | 0.0122 | 0.0216 | 0.0177 | 0.8517 | 0.0429 | 0.0438 | 0.0101 |
|  | ABa-9 | 0.0074 | 0.0277 | 0.0129 | 0.7798 | 0.0562 | 0.11 | 0.006 |
|  | ABa-10 | 0.1032 | 0.0078 | 0.0192 | 0.7202 | 0.0808 | 0.0378 | 0.031 |
|  | ABa-11 | 0.0055 | 0.006 | 0.0066 | 0.9415 | 0.006 | 0.0086 | 0.0258 |
|  | ABa-12 | 0.0268 | 0.003 | 0.0097 | 0.8781 | 0.007 | 0.0718 | 0.0036 |
| S21 | JZa-1 | 0.0279 | 0.0174 | 0.0324 | 0.7947 | 0.0852 | 0.0324 | 0.01 |
|  | JZa-2 | 0.0064 | 0.005 | 0.007 | 0.93 | 0.0124 | 0.0257 | 0.0135 |
|  | JZa-3 | 0.0054 | 0.0044 | 0.0105 | 0.0489 | 0.0051 | 0.913 | 0.0127 |
|  | JZa-4 | 0.0175 | 0.0066 | 0.0104 | 0.1714 | 0.0173 | 0.6999 | 0.0768 |
|  | JZa-5 | 0.0076 | 0.01 | 0.0055 | 0.0094 | 0.005 | 0.9427 | 0.0198 |
|  | JZa-6 | 0.0561 | 0.01 | 0.0155 | 0.6897 | 0.013 | 0.1474 | 0.0682 |
|  | JZa-7 | 0.0071 | 0.005 | 0.0122 | 0.3509 | 0.5468 | 0.0583 | 0.0196 |
|  | JZa-8 | 0.0138 | 0.0206 | 0.032 | 0.0448 | 0.164 | 0.6845 | 0.0403 |
|  | JZa-9 | 0.0216 | 0.006 | 0.0062 | 0.2156 | 0.0662 | 0.6476 | 0.0367 |
|  | JZa-10 | 0.052 | 0.0054 | 0.0324 | 0.705 | 0.0246 | 0.171 | 0.0096 |
|  | JZa-11 | 0.0086 | 0.0084 | 0.0078 | 0.8959 | 0.0418 | 0.0155 | 0.022 |
|  | JZa-12 | 0.0091 | 0.005 | 0.011 | 0.7753 | 0.0756 | 0.0867 | 0.0373 |
|  | JZa-13 | 0.0183 | 0.0108 | 0.0111 | 0.9092 | 0.0076 | 0.0213 | 0.0217 |
|  | JZa-14 | 0.0261 | 0.0336 | 0.0149 | 0.231 | 0.0643 | 0.6226 | 0.0075 |
|  | JZa-15 | 0.004 | 0.0036 | 0.0042 | 0.006 | 0.0076 | 0.9696 | 0.005 |
|  | JZa-16 | 0.0148 | 0.0074 | 0.0182 | 0.0312 | 0.0146 | 0.8936 | 0.0202 |
| S22 | YS1a-1 | 0.007 | 0.003 | 0.0129 | 0.7128 | 0.004 | 0.1534 | 0.1069 |
|  | YS1a-2 | 0.005 | 0.0069 | 0.0063 | 0.9471 | 0.0152 | 0.0145 | 0.005 |
|  | YS1a-3 | 0.0046 | 0.0066 | 0.0052 | 0.8151 | 0.0219 | 0.033 | 0.1136 |
|  | YS1a-4 | 0.0062 | 0.003 | 0.0134 | 0.9447 | 0.0193 | 0.0084 | 0.005 |
|  | YS1a-5 | 0.006 | 0.004 | 0.0073 | 0.9568 | 0.0049 | 0.0098 | 0.0112 |
|  | YS1a-6 | 0.005 | 0.0034 | 0.0086 | 0.9466 | 0.0061 | 0.0185 | 0.0118 |
|  | YS1a-7 | 0.0054 | 0.004 | 0.0061 | 0.9654 | 0.0094 | 0.0057 | 0.004 |
|  | YS1a-8 | 0.0918 | 0.0323 | 0.2811 | 0.4685 | 0.021 | 0.0851 | 0.0202 |
|  | YS1a-9 | 0.005 | 0.006 | 0.0054 | 0.0107 | 0.0134 | 0.9555 | 0.004 |
|  | YS1a-10 | 0.012 | 0.0339 | 0.0254 | 0.003 | 0.007 | 0.8874 | 0.0313 |
|  | YS1a-11 | 0.008 | 0.0218 | 0.0152 | 0.2184 | 0.0534 | 0.6611 | 0.0221 |
|  | YS1a-12 | 0.011 | 0.0034 | 0.0191 | 0.161 | 0.0638 | 0.7306 | 0.0111 |
|  | YS1a-13 | 0.0051 | 0.003 | 0.0058 | 0.9463 | 0.0054 | 0.0284 | 0.006 |
|  | YS1a-14 | 0.004 | 0.003 | 0.0052 | 0.9556 | 0.013 | 0.0145 | 0.0047 |
|  | YS1a-15 | 0.007 | 0.0066 | 0.0315 | 0.9119 | 0.022 | 0.0125 | 0.0085 |
|  | YS1a-16 | 0.0044 | 0.0041 | 0.0074 | 0.9501 | 0.0092 | 0.0128 | 0.012 |
|  | YS1a-17 | 0.0078 | 0.0042 | 0.0068 | 0.7408 | 0.0728 | 0.0623 | 0.1052 |
|  | YS1a-18 | 0.0078 | 0.003 | 0.0095 | 0.9518 | 0.0056 | 0.0162 | 0.0061 |
| S23 | YS2a-1 | 0.0096 | 0.003 | 0.0049 | 0.9576 | 0.0062 | 0.0127 | 0.006 |
|  | YS2a-2 | 0.0046 | 0.0102 | 0.0095 | 0.5778 | 0.0064 | 0.3811 | 0.0104 |
|  | YS2a-3 | 0.0066 | 0.004 | 0.0062 | 0.0092 | 0.0036 | 0.9656 | 0.0048 |
|  | YS2a-4 | 0.0151 | 0.006 | 0.0132 | 0.0558 | 0.0069 | 0.8451 | 0.0579 |
|  | YS2a-5 | 0.0702 | 0.0044 | 0.1554 | 0.0383 | 0.0056 | 0.7176 | 0.0085 |
|  | YS2a-6 | 0.006 | 0.0056 | 0.0056 | 0.9373 | 0.0266 | 0.0149 | 0.004 |
|  | YS2a-7 | 0.0051 | 0.004 | 0.0066 | 0.9348 | 0.0186 | 0.0249 | 0.006 |
|  | YS2a-8 | 0.0064 | 0.0074 | 0.0198 | 0.9394 | 0.0103 | 0.012 | 0.0047 |
|  | YS2a-9 | 0.004 | 0.003 | 0.005 | 0.8703 | 0.005 | 0.0475 | 0.0651 |
|  | YS2a-10 | 0.005 | 0.0058 | 0.0072 | 0.4538 | 0.0081 | 0.5141 | 0.006 |
|  | YS2a-11 | 0.007 | 0.0093 | 0.0116 | 0.2822 | 0.0069 | 0.5107 | 0.1723 |
|  | YS2a-12 | 0.004 | 0.0201 | 0.007 | 0.9364 | 0.004 | 0.0155 | 0.013 |
|  | YS2a-13 | 0.005 | 0.0134 | 0.0091 | 0.1016 | 0.0295 | 0.8342 | 0.0073 |
|  | YS2a-14 | 0.3246 | 0.0104 | 0.0242 | 0.1617 | 0.2921 | 0.179 | 0.008 |
|  | YS2a-15 | 0.011 | 0.0044 | 0.0168 | 0.0324 | 0.0259 | 0.9032 | 0.0063 |
|  | YS2a-16 | 0.0136 | 0.0084 | 0.0409 | 0.8955 | 0.005 | 0.0286 | 0.008 |
|  | YS2a-17 | 0.005 | 0.005 | 0.0125 | 0.3207 | 0.046 | 0.6062 | 0.0046 |
| S24 | YS3a-1 | 0.003 | 0.003 | 0.0046 | 0.9756 | 0.005 | 0.0058 | 0.003 |
|  | YS3a-2 | 0.0044 | 0.004 | 0.0041 | 0.9719 | 0.0066 | 0.006 | 0.003 |
|  | YS3a-3 | 0.004 | 0.0031 | 0.0045 | 0.972 | 0.0064 | 0.006 | 0.004 |
|  | YS3a-4 | 0.004 | 0.003 | 0.005 | 0.973 | 0.005 | 0.006 | 0.004 |
|  | YS3a-5 | 0.0114 | 0.0074 | 0.0078 | 0.868 | 0.0323 | 0.0681 | 0.005 |
|  | YS3a-6 | 0.0103 | 0.004 | 0.0065 | 0.6586 | 0.3096 | 0.007 | 0.004 |
|  | YS3a-7 | 0.0101 | 0.0094 | 0.0086 | 0.9236 | 0.0254 | 0.0112 | 0.0117 |
|  | YS3a-8 | 0.1649 | 0.006 | 0.053 | 0.7209 | 0.0056 | 0.0431 | 0.0065 |
|  | YS3a-9 | 0.0299 | 0.007 | 0.0302 | 0.0286 | 0.0052 | 0.8945 | 0.0046 |
|  | YS3a-10 | 0.0056 | 0.0544 | 0.0054 | 0.0117 | 0.004 | 0.9091 | 0.0098 |
| S25 | NQa-1 | 0.0215 | 0.0046 | 0.1161 | 0.0179 | 0.0206 | 0.8156 | 0.0037 |
|  | NQa-2 | 0.0058 | 0.008 | 0.0117 | 0.9411 | 0.0106 | 0.0138 | 0.009 |
|  | NQa-3 | 0.0049 | 0.004 | 0.0068 | 0.9576 | 0.0124 | 0.0094 | 0.0049 |
|  | NQa-4 | 0.0096 | 0.0054 | 0.0094 | 0.936 | 0.0086 | 0.0144 | 0.0166 |
|  | NQa-5 | 0.0162 | 0.0056 | 0.0048 | 0.9432 | 0.0192 | 0.005 | 0.006 |
|  | NQa-6 | 0.007 | 0.0091 | 0.0054 | 0.946 | 0.023 | 0.006 | 0.0035 |
|  | NQa-7 | 0.0048 | 0.0082 | 0.0063 | 0.956 | 0.0079 | 0.0108 | 0.006 |
|  | NQa-8 | 0.004 | 0.003 | 0.004 | 0.9693 | 0.0071 | 0.0086 | 0.004 |
|  | NQa-9 | 0.0092 | 0.0057 | 0.006 | 0.9602 | 0.0059 | 0.009 | 0.004 |
|  | NQa-10 | 0.0159 | 0.011 | 0.1869 | 0.2304 | 0.041 | 0.499 | 0.0159 |
|  | NQa-11 | 0.0084 | 0.0064 | 0.0168 | 0.0883 | 0.0054 | 0.8573 | 0.0174 |
|  | NQa-12 | 0.0081 | 0.006 | 0.0105 | 0.0358 | 0.0117 | 0.905 | 0.0229 |
|  | NQa-13 | 0.009 | 0.007 | 0.008 | 0.9436 | 0.005 | 0.0154 | 0.012 |
|  | NQa-14 | 0.0157 | 0.007 | 0.0113 | 0.9357 | 0.004 | 0.0145 | 0.0118 |
|  | NQa-15 | 0.0056 | 0.0036 | 0.0052 | 0.9709 | 0.005 | 0.0067 | 0.003 |
|  | NQa-16 | 0.2046 | 0.0058 | 0.0878 | 0.6683 | 0.009 | 0.0195 | 0.005 |
|  | NQa-17 | 0.0049 | 0.0044 | 0.0107 | 0.8874 | 0.007 | 0.0802 | 0.0054 |
|  | NQa-18 | 0.0062 | 0.017 | 0.0156 | 0.8599 | 0.0287 | 0.0096 | 0.0631 |
|  | NQa-19 | 0.008 | 0.0044 | 0.007 | 0.9437 | 0.006 | 0.021 | 0.0099 |
| S26 | YS4a-1 | 0.0074 | 0.0053 | 0.0134 | 0.9477 | 0.0074 | 0.0128 | 0.006 |
|  | YS4a-2 | 0.0068 | 0.0324 | 0.0626 | 0.614 | 0.0165 | 0.1931 | 0.0746 |
|  | YS4a-3 | 0.0046 | 0.0064 | 0.0105 | 0.8158 | 0.0149 | 0.1404 | 0.0074 |
|  | YS4a-4 | 0.008 | 0.0043 | 0.0056 | 0.0268 | 0.004 | 0.9419 | 0.0094 |
|  | YS4a-5 | 0.0084 | 0.0076 | 0.0118 | 0.0463 | 0.009 | 0.9128 | 0.004 |
|  | YS4a-6 | 0.0116 | 0.0044 | 0.0688 | 0.0042 | 0.005 | 0.9013 | 0.0047 |
|  | YS4a-7 | 0.0138 | 0.003 | 0.0105 | 0.926 | 0.0054 | 0.0373 | 0.004 |
|  | YS4a-8 | 0.01 | 0.0059 | 0.0062 | 0.9546 | 0.007 | 0.006 | 0.0103 |
|  | YS4a-9 | 0.007 | 0.0047 | 0.008 | 0.9619 | 0.0074 | 0.004 | 0.007 |
|  | YS4a-10 | 0.0058 | 0.0099 | 0.0096 | 0.9477 | 0.0096 | 0.0114 | 0.006 |
|  | YS4a-11 | 0.0066 | 0.012 | 0.008 | 0.9288 | 0.0268 | 0.0112 | 0.0066 |
|  | YS4a-12 | 0.0056 | 0.0044 | 0.0172 | 0.9424 | 0.0086 | 0.0158 | 0.006 |
| S27 | LWQ1a-1 | 0.0066 | 0.0058 | 0.0168 | 0.9103 | 0.0334 | 0.0187 | 0.0084 |
|  | LWQ1a-2 | 0.004 | 0.0068 | 0.0046 | 0.9652 | 0.0078 | 0.006 | 0.0056 |
|  | LWQ1a-3 | 0.0159 | 0.0036 | 0.0361 | 0.5301 | 0.008 | 0.4021 | 0.0043 |
|  | LWQ1a-4 | 0.006 | 0.0048 | 0.0058 | 0.007 | 0.0031 | 0.9663 | 0.007 |
|  | LWQ1a-5 | 0.0725 | 0.0279 | 0.1483 | 0.0298 | 0.0046 | 0.7098 | 0.0071 |
|  | LWQ1a-6 | 0.0139 | 0.0088 | 0.1221 | 0.1577 | 0.005 | 0.6875 | 0.005 |
|  | LWQ1a-7 | 0.006 | 0.0048 | 0.0102 | 0.7425 | 0.005 | 0.2265 | 0.005 |
|  | LWQ1a-8 | 0.0153 | 0.0111 | 0.018 | 0.9212 | 0.0095 | 0.0124 | 0.0125 |
|  | LWQ1a-9 | 0.005 | 0.004 | 0.0044 | 0.9708 | 0.0081 | 0.004 | 0.0037 |
|  | LWQ1a-10 | 0.0044 | 0.006 | 0.008 | 0.9569 | 0.0116 | 0.0076 | 0.0055 |
|  | LWQ1a-11 | 0.009 | 0.004 | 0.0066 | 0.9584 | 0.005 | 0.01 | 0.007 |
|  | LWQ1a-12 | 0.0058 | 0.0044 | 0.0171 | 0.9529 | 0.0074 | 0.0079 | 0.0045 |
|  | LWQ1a-13 | 0.004 | 0.0036 | 0.0048 | 0.9611 | 0.0076 | 0.0133 | 0.0056 |
|  | LWQ1a-14 | 0.0086 | 0.0133 | 0.012 | 0.3587 | 0.0389 | 0.5574 | 0.0111 |
|  | LWQ1a-15 | 0.0069 | 0.0034 | 0.0066 | 0.8973 | 0.0089 | 0.0719 | 0.005 |
|  | LWQ1a-16 | 0.0158 | 0.003 | 0.0053 | 0.0084 | 0.0036 | 0.9609 | 0.003 |
| S28 | LWQ2a-1 | 0.1231 | 0.0159 | 0.1056 | 0.2733 | 0.016 | 0.4524 | 0.0137 |
|  | LWQ2a-2 | 0.0056 | 0.004 | 0.0092 | 0.1125 | 0.0061 | 0.8515 | 0.0111 |
|  | LWQ2a-3 | 0.0088 | 0.0084 | 0.0125 | 0.9475 | 0.0054 | 0.009 | 0.0084 |
|  | LWQ2a-4 | 0.0107 | 0.0092 | 0.0101 | 0.9216 | 0.0072 | 0.0128 | 0.0284 |
|  | LWQ2a-5 | 0.0126 | 0.035 | 0.0098 | 0.9126 | 0.0083 | 0.0126 | 0.0091 |
|  | LWQ2a-6 | 0.004 | 0.0054 | 0.0121 | 0.9422 | 0.005 | 0.0273 | 0.004 |
|  | LWQ2a-7 | 0.004 | 0.0122 | 0.0159 | 0.953 | 0.006 | 0.0059 | 0.003 |
|  | LWQ2a-8 | 0.0076 | 0.004 | 0.0091 | 0.9586 | 0.004 | 0.0097 | 0.007 |
|  | LWQ2a-9 | 0.006 | 0.004 | 0.0064 | 0.9561 | 0.0164 | 0.0061 | 0.005 |
|  | LWQ2a-10 | 0.0046 | 0.0044 | 0.009 | 0.9574 | 0.0084 | 0.01 | 0.0062 |
|  | LWQ2a-11 | 0.0197 | 0.0045 | 0.0687 | 0.2464 | 0.0074 | 0.6477 | 0.0056 |
|  | LWQ2a-12 | 0.0204 | 0.0066 | 0.0247 | 0.3457 | 0.0046 | 0.5894 | 0.0086 |
|  | LWQ2a-13 | 0.033 | 0.0335 | 0.2754 | 0.2558 | 0.0169 | 0.3762 | 0.0092 |
|  | LWQ2a-14 | 0.0132 | 0.0082 | 0.0087 | 0.8758 | 0.0239 | 0.061 | 0.0091 |
|  | LWQ2a-15 | 0.0108 | 0.048 | 0.0416 | 0.8735 | 0.006 | 0.0095 | 0.0106 |
|  | LWQ2a-16 | 0.0041 | 0.005 | 0.0066 | 0.8886 | 0.0692 | 0.0179 | 0.0086 |
|  | LWQ2a-17 | 0.0082 | 0.005 | 0.0065 | 0.759 | 0.0093 | 0.2044 | 0.0076 |
|  | LWQ2a-18 | 0.0156 | 0.0148 | 0.0273 | 0.8047 | 0.0366 | 0.0947 | 0.0063 |
|  | LWQ2a-19 | 0.0088 | 0.0127 | 0.0088 | 0.9304 | 0.0107 | 0.0174 | 0.0112 |
|  | LWQ2a-20 | 0.0066 | 0.0203 | 0.006 | 0.9226 | 0.023 | 0.0095 | 0.012 |
| S29 | DQa-1 | 0.0403 | 0.0058 | 0.053 | 0.6164 | 0.2369 | 0.0342 | 0.0133 |
|  | DQa-2 | 0.004 | 0.006 | 0.005 | 0.968 | 0.006 | 0.006 | 0.005 |
|  | DQa-3 | 0.0087 | 0.0054 | 0.0104 | 0.3193 | 0.0111 | 0.6338 | 0.0113 |
|  | DQa-4 | 0.0109 | 0.0068 | 0.1181 | 0.0993 | 0.004 | 0.7494 | 0.0114 |
|  | DQa-5 | 0.064 | 0.0055 | 0.0224 | 0.2945 | 0.0109 | 0.58 | 0.0227 |
|  | DQa-6 | 0.2036 | 0.0081 | 0.0137 | 0.1425 | 0.0086 | 0.5639 | 0.0596 |
|  | DQa-7 | 0.0251 | 0.008 | 0.0157 | 0.251 | 0.0146 | 0.666 | 0.0196 |
|  | DQa-8 | 0.0466 | 0.0495 | 0.1076 | 0.181 | 0.0357 | 0.5386 | 0.041 |
|  | DQa-9 | 0.0289 | 0.003 | 0.0044 | 0.9383 | 0.0141 | 0.0072 | 0.004 |
|  | DQa-10 | 0.0098 | 0.0278 | 0.0094 | 0.882 | 0.0103 | 0.026 | 0.0347 |
|  | DQa-11 | 0.0108 | 0.004 | 0.0176 | 0.933 | 0.0114 | 0.0142 | 0.009 |
|  | DQa-12 | 0.0794 | 0.0351 | 0.0754 | 0.6458 | 0.0222 | 0.0628 | 0.0794 |
|  | DQa-13 | 0.0157 | 0.0618 | 0.0261 | 0.7011 | 0.1144 | 0.0555 | 0.0255 |
|  | DQa-14 | 0.0464 | 0.0256 | 0.0581 | 0.7468 | 0.1008 | 0.008 | 0.0143 |
|  | DQa-15 | 0.0222 | 0.0138 | 0.035 | 0.8114 | 0.009 | 0.0508 | 0.0579 |
| S30 | BQa-1 | 0.1571 | 0.0221 | 0.3214 | 0.3888 | 0.0046 | 0.0963 | 0.0098 |
|  | BQa-2 | 0.3523 | 0.005 | 0.3302 | 0.1959 | 0.0158 | 0.0962 | 0.0046 |
|  | BQa-3 | 0.0082 | 0.0185 | 0.0157 | 0.0919 | 0.7446 | 0.079 | 0.0422 |
|  | BQa-4 | 0.0349 | 0.0072 | 0.011 | 0.0158 | 0.9147 | 0.0088 | 0.0076 |
|  | BQa-5 | 0.0066 | 0.0084 | 0.019 | 0.0092 | 0.9441 | 0.0081 | 0.0046 |
|  | BQa-6 | 0.0273 | 0.0566 | 0.0549 | 0.025 | 0.8068 | 0.0213 | 0.0082 |
|  | BQa-7 | 0.0058 | 0.0241 | 0.029 | 0.0173 | 0.9132 | 0.004 | 0.0066 |
|  | BQa-8 | 0.0194 | 0.0116 | 0.0257 | 0.0704 | 0.8243 | 0.0338 | 0.0148 |
|  | BQa-9 | 0.006 | 0.0066 | 0.0093 | 0.0179 | 0.9247 | 0.0299 | 0.0056 |
|  | BQa-10 | 0.0327 | 0.0121 | 0.0387 | 0.0235 | 0.8624 | 0.0226 | 0.008 |
|  | BQa-11 | 0.013 | 0.0185 | 0.0397 | 0.8884 | 0.0227 | 0.0061 | 0.0116 |
|  | BQa-12 | 0.0076 | 0.005 | 0.0087 | 0.0773 | 0.0104 | 0.8854 | 0.0056 |
|  | BQa-13 | 0.0623 | 0.1057 | 0.2801 | 0.439 | 0.0074 | 0.0978 | 0.0078 |
|  | BQa-14 | 0.0052 | 0.005 | 0.0079 | 0.2694 | 0.3042 | 0.4036 | 0.0047 |
|  | BQa-15 | 0.0212 | 0.0123 | 0.0537 | 0.1363 | 0.7055 | 0.0497 | 0.0213 |
|  | BQa-16 | 0.0071 | 0.004 | 0.0092 | 0.038 | 0.9187 | 0.0175 | 0.0056 |
|  | BQa-17 | 0.0046 | 0.0081 | 0.0066 | 0.0191 | 0.9464 | 0.0106 | 0.0046 |
|  | BQa-18 | 0.006 | 0.0062 | 0.0467 | 0.0136 | 0.8947 | 0.0232 | 0.0096 |
|  | BQa-19 | 0.1345 | 0.004 | 0.1022 | 0.0063 | 0.7339 | 0.015 | 0.0041 |
|  | BQa-20 | 0.344 | 0.006 | 0.2194 | 0.0853 | 0.2966 | 0.0437 | 0.005 |
|  | BQa-21 | 0.008 | 0.0413 | 0.012 | 0.0116 | 0.9169 | 0.0062 | 0.004 |
|  | BQa-22 | 0.0087 | 0.004 | 0.014 | 0.0216 | 0.934 | 0.0114 | 0.0063 |
|  | BQa-23 | 0.015 | 0.0485 | 0.0462 | 0.3709 | 0.0231 | 0.4809 | 0.0154 |
|  | BQa-24 | 0.0381 | 0.0063 | 0.0146 | 0.0797 | 0.2554 | 0.5912 | 0.0147 |
| S31 | CDa-1 | 0.0093 | 0.004 | 0.0114 | 0.0634 | 0.0079 | 0.8964 | 0.0076 |
|  | CDa-2 | 0.4878 | 0.0096 | 0.2467 | 0.0209 | 0.0565 | 0.1728 | 0.0056 |
|  | CDa-3 | 0.0041 | 0.006 | 0.0052 | 0.004 | 0.9697 | 0.006 | 0.005 |
|  | CDa-4 | 0.0045 | 0.003 | 0.0046 | 0.0066 | 0.9737 | 0.0046 | 0.003 |
|  | CDa-5 | 0.0072 | 0.0056 | 0.004 | 0.005 | 0.9697 | 0.0045 | 0.004 |
|  | CDa-6 | 0.005 | 0.003 | 0.0036 | 0.0056 | 0.9752 | 0.004 | 0.0036 |
|  | CDa-7 | 0.0044 | 0.0179 | 0.0073 | 0.0101 | 0.9397 | 0.005 | 0.0156 |
|  | CDa-8 | 0.0188 | 0.0082 | 0.0188 | 0.0056 | 0.9236 | 0.02 | 0.005 |
|  | CDa-9 | 0.0098 | 0.004 | 0.0238 | 0.081 | 0.8686 | 0.0088 | 0.004 |
|  | CDa-10 | 0.0054 | 0.0034 | 0.0456 | 0.0533 | 0.8801 | 0.009 | 0.0032 |
|  | CDa-11 | 0.0172 | 0.0034 | 0.0132 | 0.6601 | 0.2889 | 0.0132 | 0.004 |
|  | CDa-12 | 0.0491 | 0.004 | 0.1584 | 0.005 | 0.208 | 0.57 | 0.0056 |
|  | CDa-13 | 0.0766 | 0.0078 | 0.1094 | 0.006 | 0.1847 | 0.5187 | 0.0967 |
|  | CDa-14 | 0.0218 | 0.005 | 0.0077 | 0.0549 | 0.7931 | 0.1101 | 0.0074 |
|  | CDa-15 | 0.004 | 0.004 | 0.003 | 0.005 | 0.976 | 0.004 | 0.004 |
|  | CDa-16 | 0.0177 | 0.0092 | 0.0124 | 0.0447 | 0.894 | 0.0061 | 0.0159 |
|  | CDa-17 | 0.0078 | 0.0275 | 0.0181 | 0.0408 | 0.8764 | 0.0214 | 0.008 |
|  | CDa-18 | 0.0044 | 0.0046 | 0.003 | 0.005 | 0.9758 | 0.004 | 0.0032 |
|  | CDa-19 | 0.004 | 0.003 | 0.003 | 0.0047 | 0.9793 | 0.003 | 0.003 |
|  | CDa-20 | 0.0048 | 0.0036 | 0.0036 | 0.005 | 0.9705 | 0.0068 | 0.0057 |
|  | CDa-21 | 0.009 | 0.0908 | 0.0468 | 0.0165 | 0.7996 | 0.0218 | 0.0155 |
|  | CDa-22 | 0.004 | 0.005 | 0.0046 | 0.004 | 0.9734 | 0.004 | 0.005 |
|  | CDa-23 | 0.027 | 0.0495 | 0.0212 | 0.0699 | 0.8141 | 0.0129 | 0.0054 |
|  | CDa-24 | 0.0067 | 0.0052 | 0.0069 | 0.008 | 0.0071 | 0.9561 | 0.01 |
| S32 | JDa-1 | 0.0959 | 0.0305 | 0.0269 | 0.7212 | 0.0076 | 0.0889 | 0.0289 |
|  | JDa-2 | 0.0064 | 0.0074 | 0.0157 | 0.5749 | 0.3105 | 0.0747 | 0.0104 |
|  | JDa-3 | 0.006 | 0.004 | 0.0058 | 0.0207 | 0.9395 | 0.0116 | 0.0124 |
|  | JDa-4 | 0.004 | 0.003 | 0.0062 | 0.0192 | 0.96 | 0.0046 | 0.003 |
|  | JDa-5 | 0.0054 | 0.003 | 0.003 | 0.0112 | 0.9702 | 0.0032 | 0.004 |
|  | JDa-6 | 0.1241 | 0.0033 | 0.0185 | 0.0098 | 0.8226 | 0.0151 | 0.0066 |
|  | JDa-7 | 0.0229 | 0.0046 | 0.0153 | 0.005 | 0.8976 | 0.0457 | 0.009 |
|  | JDa-8 | 0.0043 | 0.0036 | 0.0055 | 0.0076 | 0.9722 | 0.0038 | 0.003 |
|  | JDa-9 | 0.0078 | 0.0183 | 0.005 | 0.0087 | 0.9487 | 0.006 | 0.0055 |
|  | JDa-10 | 0.0094 | 0.003 | 0.005 | 0.006 | 0.9676 | 0.005 | 0.004 |
|  | JDa-11 | 0.011 | 0.0062 | 0.0483 | 0.7849 | 0.0934 | 0.0424 | 0.0138 |
|  | JDa-12 | 0.013 | 0.0033 | 0.0143 | 0.0118 | 0.011 | 0.939 | 0.0076 |
|  | JDa-13 | 0.006 | 0.003 | 0.0063 | 0.0082 | 0.005 | 0.9665 | 0.005 |
|  | JDa-14 | 0.0076 | 0.0054 | 0.0119 | 0.2959 | 0.0419 | 0.6281 | 0.0092 |
| S33 | DGa-1 | 0.0072 | 0.0033 | 0.0046 | 0.0309 | 0.9338 | 0.0152 | 0.005 |
|  | DGa-2 | 0.0417 | 0.0056 | 0.0204 | 0.0122 | 0.9007 | 0.0144 | 0.005 |
|  | DGa-3 | 0.0043 | 0.003 | 0.0036 | 0.004 | 0.9785 | 0.0036 | 0.003 |
|  | DGa-4 | 0.0094 | 0.0064 | 0.0176 | 0.005 | 0.9343 | 0.009 | 0.0183 |
|  | DGa-5 | 0.0104 | 0.0089 | 0.0106 | 0.005 | 0.9332 | 0.0056 | 0.0263 |
|  | DGa-6 | 0.0182 | 0.003 | 0.0056 | 0.009 | 0.9501 | 0.0084 | 0.0057 |
|  | DGa-7 | 0.0048 | 0.0131 | 0.0076 | 0.0064 | 0.9537 | 0.0051 | 0.0093 |
|  | DGa-8 | 0.004 | 0.0084 | 0.008 | 0.0113 | 0.9564 | 0.0064 | 0.0055 |
|  | DGa-9 | 0.0243 | 0.006 | 0.0187 | 0.1698 | 0.7218 | 0.0534 | 0.006 |
|  | DGa-10 | 0.1642 | 0.0048 | 0.0758 | 0.0782 | 0.4117 | 0.1832 | 0.0819 |
|  | DGa-11 | 0.2572 | 0.0034 | 0.2145 | 0.0301 | 0.4556 | 0.0304 | 0.0089 |
|  | DGa-12 | 0.0768 | 0.0109 | 0.0965 | 0.2029 | 0.1456 | 0.1856 | 0.2818 |
|  | DGa-13 | 0.0088 | 0.0054 | 0.012 | 0.0511 | 0.898 | 0.0094 | 0.0153 |
|  | DGa-14 | 0.0094 | 0.0038 | 0.0154 | 0.0842 | 0.8674 | 0.0108 | 0.009 |
