# Supplementary material for: Gene Flow Results in High Genetic Similarity between Sibiraea (Rosaceae) Species in the Qinghai-Tibetan Plateau
Source: Front Plant Sci. 2016 Oct 25;7:1596. doi: 10.3389/fpls.2016.01596 (PMC5078775; doi:10.3389/fpls.2016.01596)
Supplement: Supplementary file 4 [file Table4.docx]

Table S4. Parameter estimation of approximate Bayesian computation analysis in software DIYABC.

| Parameter | mean | median | mode | q025 | q050 | q950 | q975 |
| --- | --- | --- | --- | --- | --- | --- | --- |
| N1 | 1.58E+04 | 1.62E+04 | 1.79E+04 | 9.16E+03 | 9.89E+03 | 1.96E+04 | 1.98E+04 |
| N2 | 8.36E+03 | 7.68E+03 | 6.47E+03 | 2.73E+03 | 3.21E+03 | 1.64E+04 | 1.81E+04 |
| N3 | 1.02E+04 | 1.02E+04 | 1.15E+04 | 4.29E+03 | 4.92E+03 | 1.62E+04 | 1.75E+04 |
| t1 | 6.17E+03 | 4.60E+03 | 3.13E+03 | 1.34E+03 | 1.69E+03 | 1.52E+04 | 2.03E+04 |
| r | 5.81E-01 | 6.10E-01 | 8.43E-01 | 7.64E-02 | 1.26E-01 | 9.42E-01 | 9.70E-01 |
| t2 | 3.92E+04 | 3.42E+04 | 2.50E+04 | 1.08E+04 | 1.27E+04 | 8.02E+04 | 8.75E+04 |
| NA | 1.14E+04 | 1.20E+04 | 1.68E+04 | 1.07E+03 | 2.09E+03 | 1.91E+04 | 1.95E+04 |
| Mμmic_A | 5.35E-04 | 5.09E-04 | 4.60E-04 | 3.39E-04 | 3.63E-04 | 7.85E-04 | 8.56E-04 |
| N1μmic_A | 8.17E+00 | 8.04E+00 | 7.45E+00 | 5.52E+00 | 5.94E+00 | 1.08E+01 | 1.14E+01 |
| N2μmic_A | 4.17E+00 | 3.93E+00 | 3.34E+00 | 1.83E+00 | 2.05E+00 | 7.13E+00 | 8.02E+00 |
| N3μmic_A | 5.16E+00 | 5.07E+00 | 4.57E+00 | 3.00E+00 | 3.26E+00 | 7.46E+00 | 8.19E+00 |
| t1μmic_A | 3.13E+00 | 2.34E+00 | 1.51E+00 | 7.70E-01 | 9.10E-01 | 7.52E+00 | 9.28E+00 |
| t2μmic_A | 1.99E+01 | 1.80E+01 | 1.44E+01 | 6.16E+00 | 7.28E+00 | 3.98E+01 | 4.34E+01 |
| NAμmic_A | 5.98E+00 | 5.90E+00 | 5.43E+00 | 5.90E-01 | 1.13E+00 | 1.12E+01 | 1.22E+01 |
