# Supplementary material for: Gene Flow Results in High Genetic Similarity between Sibiraea (Rosaceae) Species in the Qinghai-Tibetan Plateau
Source: Front Plant Sci. 2016 Oct 25;7:1596. doi: 10.3389/fpls.2016.01596 (PMC5078775; doi:10.3389/fpls.2016.01596)
Supplement: Supplementary file 5 [file Table5.docx]

Table S5. Factor 2 statistic of approximate Bayesian computation analysis in software DIYABC.

|  |  |  | Factor 2 | Factor 2 | Factor 2 |
| --- | --- | --- | --- | --- | --- |
| Parameter | 50% Coverage | 95% Coverage | Mean | Median | Mode |
| N1 | 0.480 | 0.968 | 0.960 | 0.964 | 0.960 |
| N2 | 0.500 | 0.954 | 0.954 | 0.958 | 0.952 |
| N3 | 0.514 | 0.954 | 0.958 | 0.962 | 0.944 |
| t1 | 0.522 | 0.956 | 0.830 | 0.794 | 0.656 |
| r | 0.506 | 0.958 | 0.748 | 0.726 | 0.592 |
| t2 | 0.512 | 0.946 | 0.942 | 0.930 | 0.886 |
| NA | 0.488 | 0.936 | 0.744 | 0.702 | 0.478 |
| Mμmic_A | 0.424 | 0.892 | 0.976 | 0.976 | 0.962 |
| N1μmic_A | 0.548 | 0.976 | 1.000 | 1.000 | 0.998 |
| N2μmic_A | 0.580 | 0.970 | 0.990 | 0.992 | 0.992 |
| N3μmic_A | 0.564 | 0.974 | 0.998 | 0.998 | 1.000 |
| t1μmic_A | 0.514 | 0.956 | 0.824 | 0.822 | 0.694 |
| t2μmic_A | 0.528 | 0.940 | 0.912 | 0.928 | 0.896 |
| NAμmic_A | 0.508 | 0.954 | 0.698 | 0.688 | 0.498 |
