# Supplementary material for: Gene Flow Results in High Genetic Similarity between Sibiraea (Rosaceae) Species in the Qinghai-Tibetan Plateau
Source: Front Plant Sci. 2016 Oct 25;7:1596. doi: 10.3389/fpls.2016.01596 (PMC5078775; doi:10.3389/fpls.2016.01596)
Supplement: Supplementary file 6 [file Table6.DOCX]

Table S6. One-tailed P-values for the Wilcoxon test for heterozygosity excess under TPM model.

| Population | Wilcoxon Test (TPM ) | |  | Population | Wilcoxon Test (TPM ) | |
| --- | --- | --- | --- | --- | --- | --- |
|  | Deficiency | Excess |  |  | Deficiency | Excess |
| P1 | 0.6289 | 0.4219 |  | S11 | 0.19141 | 0.84375 |
| P2 | 0.8438 | 0.1914 |  | S12 | 0.37109 | 0.67969 |
| P3 | 0.8438 | 0.1914 |  | S13 | 0.02734* | 0.98047 |
| P4 | 0.7266 | 0.3203 |  | S14 | 0.67969 | 0.37109 |
| P5 | - | - |  | S15 | 0.57813 | 0.47266 |
| P6 | 0.6797 | 0.3711 |  | S16 | 0.67969 | 0.37109 |
| P7 | 0.3711 | 0.6797 |  | S17 | 0.23047 | 0.80859 |
| P8 | 0.3711 | 0.6797 |  | S18 | 0.47266 | 0.57813 |
| P9 | 1.0000 | 0.0020* |  | S19 | 0.19141 | 0.84375 |
| P10 | 0.6289 | 0.4219 |  | S20 | 0.96289 | 0.09766 |
| P11 | 0.9902 | 0.0137* |  | S21 | 0.37109 | 0.67969 |
| P12 | 0.1250 | 0.9023 |  | S22 | 0.03711* | 0.97266 |
|  |  |  |  | S23 | 0.84375 | 0.19141 |
| S1 | 0.80859 | 0.23047 |  | S24 | 0.62891 | 0.42188 |
| S2 | 0.62891 | 0.42188 |  | S25 | 0.80859 | 0.23047 |
| S3 | 0.37109 | 0.67969 |  | S26 | 0.90234 | 0.125 |
| S4 | 0.19141 | 0.84375 |  | S27 | 0.72656 | 0.32031 |
| S5 | 0.76953 | 0.27344 |  | S28 | 0.37109 | 0.67969 |
| S6 | 0.15625 | 0.875 |  | S29 | 0.32031 | 0.72656 |
| S7 | 0.37109 | 0.67969 |  | S30 | 0.62891 | 0.42188 |
| S8 | 0.84375 | 0.19141 |  | S31 | 0.125 | 0.90234 |
| S9 | 0.76953 | 0.27344 |  | S32 | 0.99023 | 0.01367* |
| S10 | 0.42188 | 0.62891 |  | S33 | 0.27344 | 0.76953 |
