# Supplementary material for: Gene Flow Results in High Genetic Similarity between Sibiraea (Rosaceae) Species in the Qinghai-Tibetan Plateau
Source: Front Plant Sci. 2016 Oct 25;7:1596. doi: 10.3389/fpls.2016.01596 (PMC5078775; doi:10.3389/fpls.2016.01596)
Supplement: Supplementary file 7 [file Table7.DOCX]

Table S7. The first-generation immigrants among *Sibiraea* populations from the frequency-based method analysis in GENEGLASS.

|  | Individual | Migrated from | *P* value |
| --- | --- | --- | --- |
| *S.laevigata* | |  |  |
|  | MQb no.4 | QLb | 0.005 |
|  | DRb no.5 | PAb | 0.005 |
|  | MYb no.13 | PAb | 0.004 |
|  | HZb no.1 | PAb | 0 |
|  | PAb no.10 | MYb | 0 |
|  | XHb no.1 | MYb | 0.007 |
|  | REG1b no.1 | XHb | 0 |
|  | REG3b no.8 | YSb | 0.001 |
|  | BMb no.3 | REG3b | 0.001 |
|  | YSb no.6 | BMb | 0.001 |
| *S.angustata* | |  |  |
|  | Mqa no.2 | PAa | 0.007 |
|  | DR1a no.4 | JDa | 0.003 |
|  | QLa no.13 | DR2a | 0.005 |
|  | MYa no.14 | QLa | 0.001 |
|  | MYa no.17 | QLa | 0.003 |
|  | HZa no.6 | QLa | 0.006 |
|  | PAa no.2 | MYa | 0.002 |
|  | PAa no.11 | QLa | 0.005 |
|  | PAa no.12 | DR2a | 0.002 |
|  | REG3a no.6 | REG2a | 0.004 |
|  | REG3a no.12 | JZa | 0.001 |
|  | HYa no.7 | ABa | 0.001 |
|  | DQINa no.13 | DBa | 0.003 |
|  | DQINa no.25 | LTa | 0.001 |
|  | DCa no.17 | LTa | 0.001 |
|  | LTa no.8 | DFa | 0.005 |
|  | LTa no.17 | DBa | 0.008 |
|  | LTa no.22 | DCa | 0.007 |
|  | DFa no.7 | DBa | 0.005 |
|  | DFa no.18 | DBa | 0.003 |
|  | DBa no.16 | DFa | 0.004 |
|  | LHa no.8 | ABa | 0.002 |
|  | ABa no.12 | LWQ1a | 0.004 |
|  | YS1a no.2 | LHa | 0.003 |
|  | YS1a no.8 | YS3a | 0.007 |
|  | YS1a no.10 | REG3a | 0 |
|  | YS2a no.16 | YS4a | 0.004 |
|  | YS3a no.6 | JDa | 0.002 |
|  | YS3a no.10 | LWQ1a | 0.002 |
|  | NQa no.13 | JZa | 0 |
|  | YS4a no.5 | YS3a | 0 |
|  | YS4a no.6 | REG3a | 0.003 |
|  | LWQ2a no.17 | YS2a | 0 |
|  | BQa no.1 | DQa | 0.002 |
|  | BQa no.2 | DBa | 0.006 |
|  | BQa no.12 | HYa | 0.007 |
|  | BQa no.13 | LWQ2a | 0.006 |
|  | BQa no.24 | JDa | 0.003 |
|  | CDa no.1 | YS2a | 0 |
|  | CDa no.24 | JZa | 0.004 |
|  | JDa no.1 | NQa | 0 |
