# Supplementary material for: Gene Flow Results in High Genetic Similarity between Sibiraea (Rosaceae) Species in the Qinghai-Tibetan Plateau
Source: Front Plant Sci. 2016 Oct 25;7:1596. doi: 10.3389/fpls.2016.01596 (PMC5078775; doi:10.3389/fpls.2016.01596)
Supplement: Supplementary file 8 [file Image1.PDF]

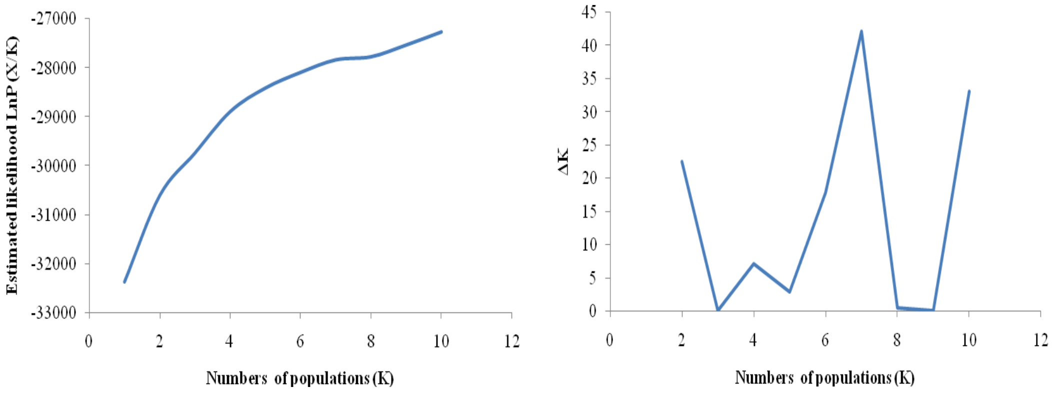

Fig. S1 Mean values for the log probability and *ΔK* (calculated according to Evanno *et al*, 2005) of the *Sibiraea* microsatellite data against the assumed number of clusters (K) calculated using STRUCTURE assuming the two species together.
